# Isolation of Elusive Fluoflavine Radicals in Two Differing Oxidation States

**DOI:** 10.1021/jacs.4c05267

**Published:** 2024-09-12

**Authors:** Florian Benner, Selvan Demir

**Affiliations:** Department of Chemistry, Michigan State University, 578 South Shaw Lane, East Lansing, Michigan 48824, United States

## Abstract

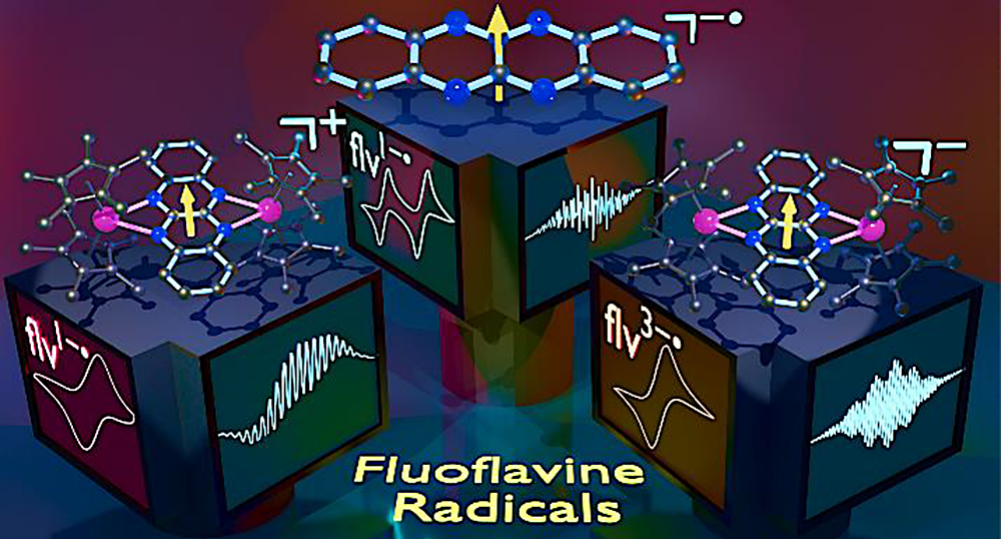

Facile access and switchability between multiple oxidation states are key properties of many catalytic applications and spintronic devices yet poorly understood due to inherent complications arising from isolating a redox system in multiple oxidation states without drastic structural changes. Here, we present the first isolable, free fluoflavine (flv) radical flv(^1–•^) as a bottleable potassium compound, [K(crypt-222)](flv^•^), **1**, and a new series of organometallic rare earth complexes [(Cp*_2_Y)_2_(μ-flv^z^)]X, (where Cp* = pentamethylcyclopentadienyl, X = [Al(OC{CF_3_}_3_)_4_]^−^ (z = −1), **2**; X = 0 (z = −2), **3**; [K(crypt-222)]^+^ (z = −3), **4**) comprising the flv ligand in three different oxidation states, two of which are paramagnetic flv^1–•^ and flv^3–•^. Excitingly, **1**, **2**, and **4** constitute the first isolable flv^1–•^ and flv^3–•^ radical complexes and, to date, the only isolated flv radicals of any oxidation state. All compounds are accessible in good crystalline yields and were unambiguously characterized via single-crystal X-ray diffraction analysis, cyclic voltammetry, IR-, UV–vis, and variable-temperature EPR spectroscopy. Remarkably, the EPR spectra for **1**, **2**, and **4** are distinct and a testament to stronger spin delocalization onto the metal centers as a function of higher charge on the flv radical. In-depth analysis of the electron- and spin density via density functional theory (DFT) calculations utilizing NLMO, QTAIM, and spin density topology analysis confirmed the fundamental interplay of metal coordination, ligand oxidation state, aromaticity, covalency, and spin density transfer, which may serve as blueprints for the development of future spintronic devices, single-molecule magnets, and quantum information science at large.

## Introduction

Multiredox reactivity in molecular compounds, specifically systems that can reversibly undergo stepwise multiple redox reactions, has attracted widespread attention for their broad range of applications such as in high energy density batteries, sensors, conductive materials, and (photoredox-)catalysis.^1−5^ Whereas multiple redox processes in the heavy main block,^6−10^ transition,^11−13^ lanthanide,^14−18^ and actinide^19−22^ metal complexes are nowadays well-established, organic molecules innate to such tunability are far less known. In fact, tripak — a variant of the archetypical pseudo-[6]oxocarbon — constitutes the first purely organic multiredox molecule with six accessible oxidation states.^23^ Importantly, organic molecules that have the ability to bridge metal ions and allow access to multiple paramagnetic oxidations are scarce.^24,25^

**Figure 1:**
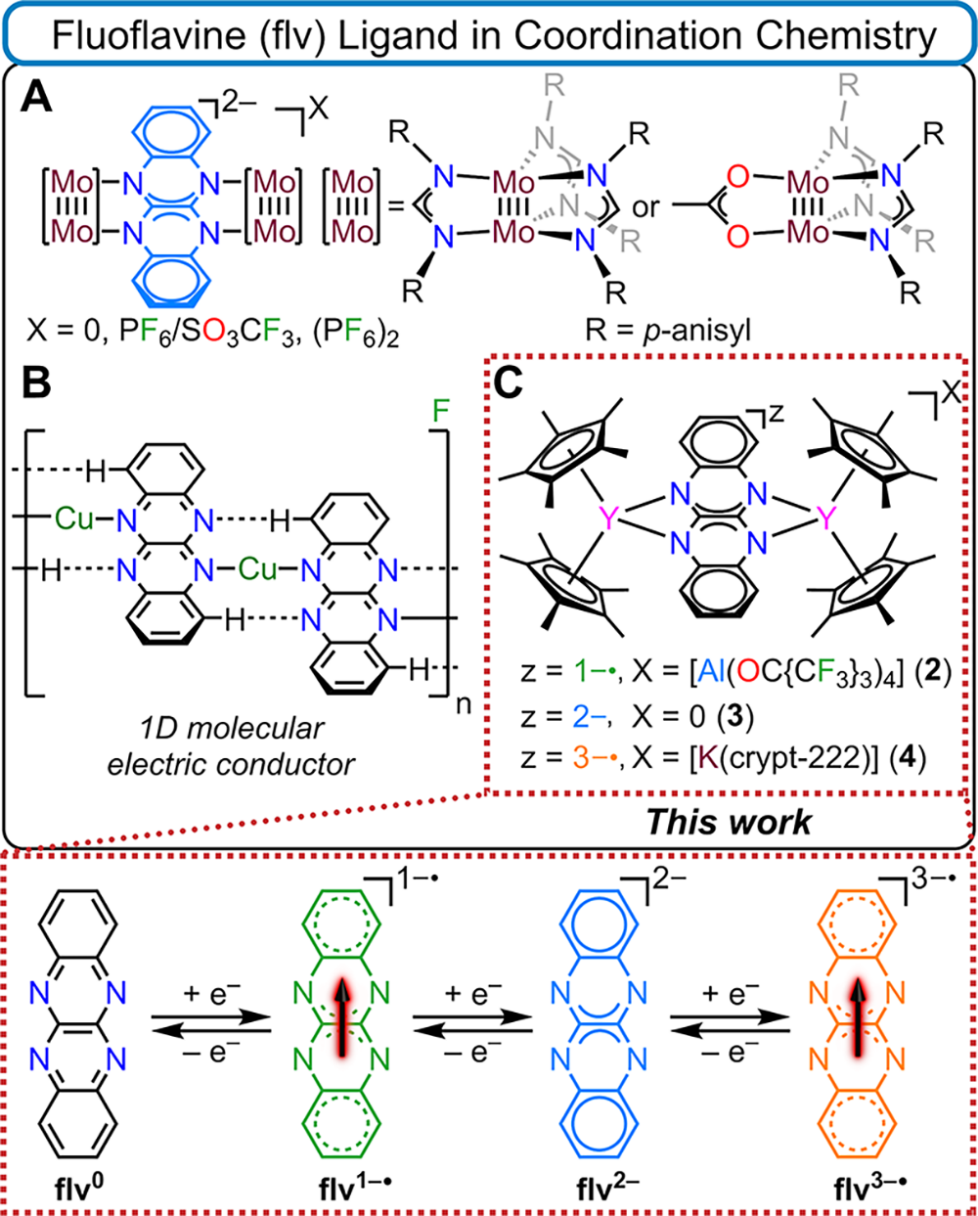
Top: (**A**) [Mo_2_(DAniF)_3_]_2_(μ-flv) (DAniF = N,N′-di-*p*-anisylformamidinate) bearing a flv^2–^ ligand, (**B**) [{Cu(flv)}F_0.5_]_n_ bearing flv^1–•/0^ ligands, elusive in crystalline form, and (**C**) isolated [(Cp*_2_Y)_2_(μ-flv^z^)]X (**2**, **3**, and **4**) complexes, reported herein, bearing flv^1–•/2–/3–•^. Bottom: Schematic representation of possible flv oxidation states where paramagnetic states are marked by red arrows.

The synthesis of strongly electron-accepting organic molecules is stimulated by the growing need for cheap, efficient, and green electronic devices.^26^ Polycyclic aromatic compounds (PACs), alongside their heteroaromatic, nitrogen-substituted polyaza analogs (NPACs), have found entry into the field of organic electronics.^26,27^ Their large π-aromatic systems give rise to properties desired for organic field effect transistors (OFETs) constituting the active materials in light-emitting diodes, conductive, and semiconductive materials.^28−30^ Here, the reorganization energy in combination with a high electron affinity and low reduction potentials are prime attributes that engender high electron conductivity.^31^ Of particular interest are linear NPACs, as their extensive tunability enables precise control over properties such as reduction potential, charge-carrier mobility, and band gap size, rendering them to be Swiss knife tools within the area of organic electronics.^31^ One of the simplest molecules within this class of compounds, 5,6,11,12-tetraazanaphthacene, TATC, and its doubly protonated version, fluoflavine, H_2_flv, are valued for their ease of accessibility and facile redox switchability.^32,33^ The coordinative ability of flv^2–^ has only been explored in two types of compounds, both involving transition metals (TMs).^34−36^ First, a series of molybdenum complexes [Mo_2_(DAniF)_3_]_2_(μ-flv) (*N*,*N*′-di-*p*-anisylformamidinate) were isolated featuring Mo–Mo-bonded units where each metal is binding to a distinct nitrogen of the fluoflavinate, flv^2–^ (Figure 1).^34^ The electronic and magnetic properties may be modulated through chemical reactivity as was demonstrated by isolating the one- and two-electron-oxidized analogs, where the oxidation occurred of the Mo centers which enabled strong magnetic coupling. Second, a flv-bridged copper 1D polymer [{Cu(flv)}F_0.5_]_n_ was isolated which exhibits semiconductor properties at room temperature via a variable-range hopping mechanism.^35,36^ The nonconductive chloride, bromide, and iodide equivalents intriguingly revealed signals in electron paramagnetic resonance (EPR) spectroscopy, presumably due to electron transfer from the Cu^I^ highest occupied to flv^0^ lowest unoccupied orbital via electron transfer at the terminal/defect moieties.^37^ Notably, these rare but all examples highlight the enormous tunability of the flv ligand and the wealth of physicochemical properties that can arise especially with respect to metal–organic hybrid materials.^38^ Importantly, to date, unambiguous structural evidence of flv radicals is lacking.

Inspired by the heavily underexplored flv ligand, we set out to place it in the vicinity of rare earth (RE) elements, which would allow access to unparalleled compounds with peerless characteristics. The reactivity and properties of the RE metals differ vastly from those observed for TMs, which is mainly attributed to the lack of *d*- and *s*-valence electrons culminating in the most stable oxidation state of +3.^39^ Especially for the lanthanides, where the valence 4*f* orbitals are substantially contracted and thus, not participating in chemical bonding, (in)organic radicals with their diffuse spin orbitals can strengthen the degree of magnetic exchange coupling between paramagnetic lanthanide ions and open shell ligands.^40−45^ Achieving strong coupling is exciting in the context of multinuclear single-molecule magnets (SMMs) as that suppresses undesirable fast relaxation pathways such as quantum tunneling of the magnetization and thus, results in real magnetic memory below a certain temperature.^41,46−51^ Their potential applications in high-density information storage and molecular spintronics,^52−56^ require slow magnetic relaxation to occur at high temperatures, close to 300 K, which can be envisioned through the synthetic design of a giant spin originating from strong coupling of multiple lanthanide ions through radical ligands. Furthermore, radical ligands can also function as building blocks for hybrid materials such as conductive magnets.^57^

The development of synthetic routes to new reactive, paramagnetic oxidation states of a ligand is challenging but pivotal for its isolation and thorough study of its electronic structure. Recognizing the potential of the flv ligand scaffold for organic electronics and SMMs, we envisioned that the fusion of both concepts would pave the way for the discovery of new functional materials relevant to future information storage and processing. Thus, we devised synthetic protocols to isolate the first flv radicals in the form of a salt and by placing them next to diamagnetic metal ions to allow complete characterization. Note, the free flv radical was probed via comproportionation reaction of flv^0^ and H_2_flv under oxygen-free conditions in a DMSO/^t^BuOH mixture, yet never isolated or unambiguously proven.^32,58^ By contrast, the redox activity of the flv^2–^ ligand was exclusively explored through oxidation reactions in nonaqueous media, where both the chemical and electrochemical results hinted at an accessible flv^1–•^ radical oxidation state, albeit no complexes were ever chemically isolated.^32,34^ With this in mind, we set out to probe the accessibility of bottleable, free flv radicals, accompanied by their full analysis. Then, we targeted the synthesis, clean isolation, and meticulous characterization of the first series of yttrium compounds containing fluoflavine ligands. First, [K(crypt-222)](flv^•^), **1**, was synthesized, containing the free monoanionic flv^1–•^ radical, which marks the first isolation of a flv^1–•^ radical of any kind and is in fact bottleable. Subsequently, the monoanionic flv^1–•^ radical was stabilized within the coordination complex [(Cp*_2_Y)_2_(μ-flv^•^)][Al(OC{CF_3_}_3_)_4_], **2** (where Cp* = pentamethylcyclopentadienyl), constituting the first unambiguous report of a crystallographically characterized flv-radical complex for any metal ion. The neutral complex [(Cp*_2_Y)_2_(μ-flv)], **3**, bearing a dianionic flv^2–^ bridge, was isolated from a salt metathesis reaction and was chemically reduced to yield [K(crypt-222)][(Cp*_2_Y)_2_(μ-flv^•^)], **4**, comprising the first ever tamed flv^3–•^ radical anion for any metal ion. Excitingly, compounds **1**, **2**, and **4** constitute the first evidence of isolable flv^1–•^ and flv^3–•^ radicals. The diamagnetic nature of Y^III^ (^89^Y, *I* = 1/2) renders yttrium compounds suitable for a detailed analysis of the metal–radical interaction, employing EPR spectroscopy and density functional theory (DFT) calculations. A thorough understanding of the electron spin density distribution has important ramifications in organic electronics and organic radical-containing SMMs, respectively.

## Results and Discussion

### Synthesis, Electrochemistry, and Spectroscopy

Dinuclear yttrocene complexes with a fluoflavine framework as a redox-active bridge were first pursued based on the neutral fluoflavine (flv) molecule, which could, in theory, provide access to various oxidation states. To this end, the parent H_2_flv was synthesized through the condensation of ortho-phenylenediamine with 2,3-dichloroquinoxaline in hot ethylene glycol, followed by filtration to give a microcrystalline golden solids.^59^ The neutral flv^0^ (also known as TATC, but for clarity will be exclusively referred to as flv^0^ herein) was attained through the oxidation of H_2_flv with excess PbO_2_ in boiling chloroform and subsequent filtration under inert conditions.^59^ After evaporating the filtrate to dryness, the resulting red-brown solid was used for analysis and synthesis without further purification.

Experiments on the electrochemical time scale (Figure S24, Table S4) shed light on the redox activity of flv^0^ with two successive, reversible reduction events occurring at *E*_1/2_^1^ = −1.73 (1) V and *E*_1/2_^2^ = −0.96(1) V vs the ferrocene/ferrocenium redox couple. Thus, we pursued the synthesis of the free flv^1–•^ radical ligand in the form of an alkali metal salt, corresponding to the first bottleable flv radical of any type. Simultaneously, this may qualify as an important entry into flv^1–•^ radical-bridged complexes such as [(Cp*_2_Y)_2_(μ-flv^•^)]^+^ (**2**^**+**^) following a salt metathesis route.

**Figure 2:**
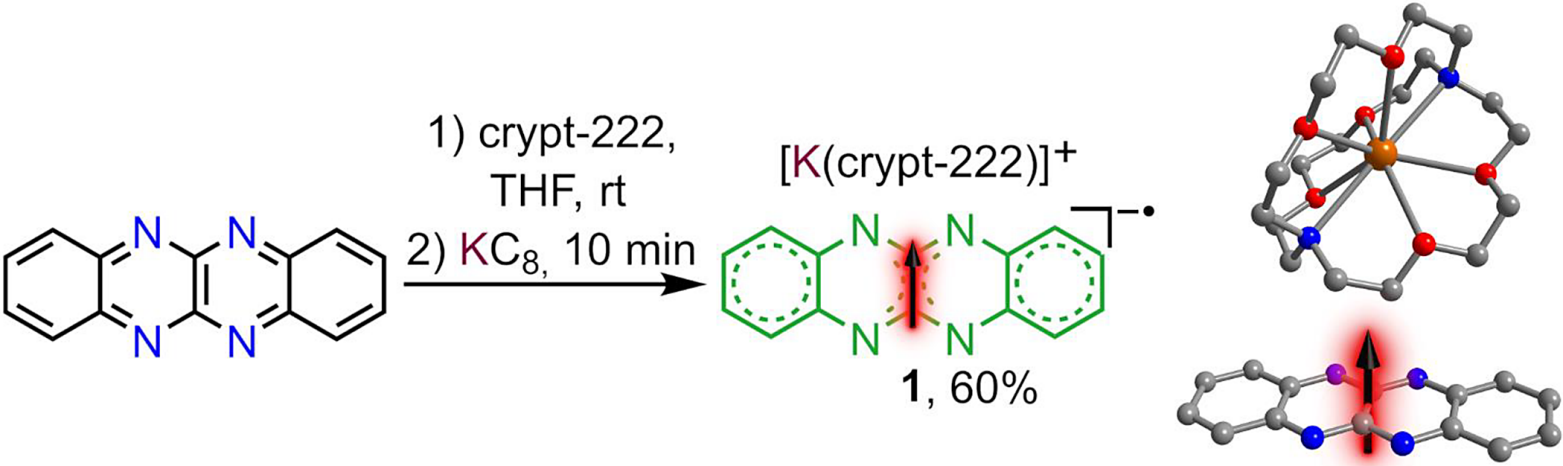
Left: synthesis of [K(crypt-222)](flv^•^) (**1**) via the reduction of flv^0^. Right: structure of compound **1**. Dark orange, red, blue, and gray spheres represent potassium, oxygen, nitrogen, and carbon atoms, respectively. All hydrogen atoms and solvent molecules are omitted for clarity.

**Figure 3:**
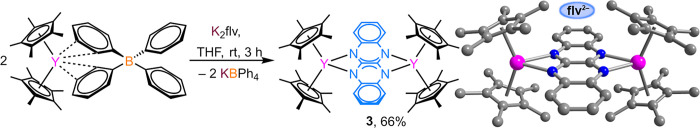
Left: synthesis of [(Cp*_2_Y)_2_(μ-flv)] (**3**) proceeded through a salt metathesis reaction of Cp*_2_Y(BPh_4_) with K_2_flv. Right: structure of **3**; pink, blue, and gray spheres represent yttrium, nitrogen, and carbon atoms, respectively. All hydrogen atoms and solvent molecules are omitted for clarity.

**Figure 4:**
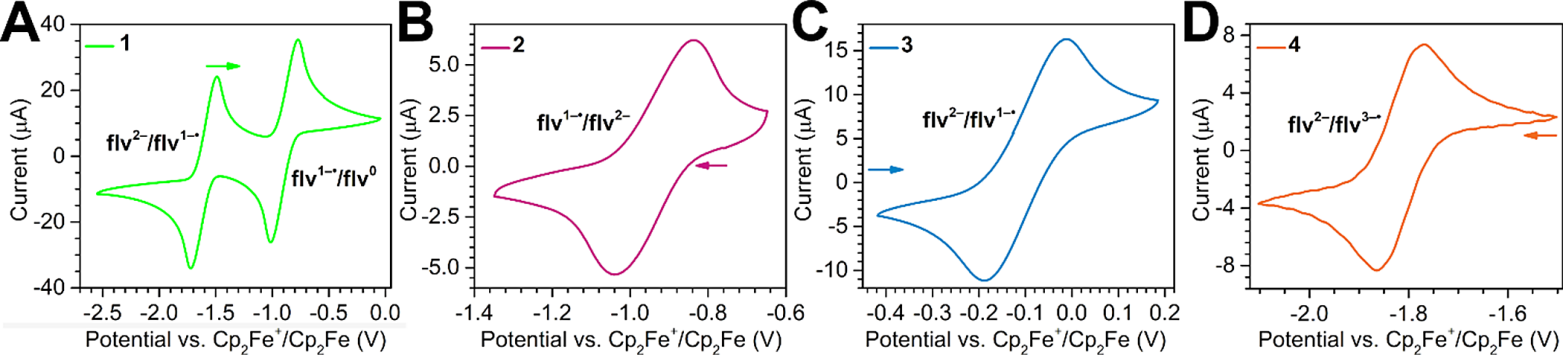
Magnification of the reversible features in cyclic voltammograms of (**A**): [K(crypt-222)](flv^•^) (**1**), (**B**) [(Cp*_2_Y)_2_(μ-flv^•^)][Al(OC{CF_3_}_3_)_4_] (**2**); (**C**) [(Cp*_2_Y)_2_(μ-flv)] (**3**); and (**D**) [K(crypt-222)][(Cp*_2_Y)_2_(μ-flv^•^)] (**4**). Compounds **1**–**3** were recorded in 1,2-difluorobenzene and **4** in THF. All electrochemical data were referenced against ferrocene. All experiments were carried out at a 100 mV/s scan rate with a (^*n*^Bu_4_N)PF_6_ supporting electrolyte. Arrows indicate the starting potential and scan direction. A summary of all determined redox potentials is given in Table S4. Full scans are shown in Figures S26, S28–S30.

Excitingly, we successfully isolated the radical oxidation state flv^1–•^ as [K(crypt-222)](flv^•^) (**1**) through one-electron reduction of flv^0^ employing the strong reducing agent potassium graphite (KC_8_) in the presence of the chelating 2.2.2-cryptand (crypt-222) (Figure 2). Dark blue single crystals of **1** were obtained from a concentrated THF solution at −35 °C over the course of 2 days in 60% yield. The radical nature of the compound was confirmed via room temperature EPR spectroscopy, which, in conjunction with the crystal structure, proves that the monoradical of flv is isolable (Figure S1 and Table S2). Electrochemical measurements on **1** exhibit two reversible reduction features at *E*_1/2_^1^ = −1.608(3) V and *E*_1/2_^2^ = −0.902(3) V, which are essentially identical to the parent flv^0^ under similar experimental conditions (Figures 4 and S25–S27, Table S4). The electrochemical results indicate that a chemical approach to flv^1–•^ functioning as a radical bridging ligand between two metal centers may be feasible under appropriate synthetic conditions. To this end, a flv^1–•^ radical-bridged dinuclear yttrium complex, [(Cp*_2_Y)_2_(μ-flv^•^)][BPh_4_] , was pursued by treating flv^0^ with KC_8_ to generate Kflv comprising the flv^1–•^ radical anion, which was subsequently transferred to a THF solution of two equiv of Cp*_2_Y(BPh_4_). An immediate color change from an off-white solution to orange-red with green fluorescence was observed alongside the formation of colorless solids, presumably KBPh_4_. Notably, although a precise addition of one equivalent of KC_8_ was carried out, the dinuclear complex, [(Cp*_2_Y)_2_(μ-flv)], **3**, formed, representing the first example of a crystallographically characterized rare earth complex containing a flv^2–^ dianion (Figures 3 and S3, Tables 1 and S2). **3** also corresponds to the first organometallic complex innate to a flv ligand for any metal ion. Repetitive experiments following analogous procedures yielded consistently **3**, instead of a complex containing the singly reduced flv^1–•^ radical as was anticipated based on the well-separated reversible redox features of flv^1–•^. Compound **3** was crystallized from concentrated THF solutions at −35 °C in 24% yield.

Following its discovery, salt metathesis as an alternative synthetic path was explored, which almost tripled the yield of **3**. For this, H_2_flv was deprotonated with potassium bis(trimethylsilyl)amide (KN*) in THF to give K_2_flv as a yellow powder in 77% yield. The reaction of K_2_flv with two equivalents of Cp*_2_Y(BPh_4_) afforded **3** and the byproduct KBPh_4_. Recrystallization of the crude product from THF at 60 °C and subsequent cooling and storage at −35 °C gave **3** in 66% crystalline yield (Figure 3).

Compound **3** comprises two yttrium ions, each ligated by two Cp* ligands, and a tetradentate flv^2–^ ligand that is coordinated to both metal centers symmetrically through two nitrogen atoms on each site, ultimately acting as a bridge. Compound **3** crystallizes in the triclinic space group *P*–1 alongside two THF molecules (Figure S3). An inversion center resides on the bridging flv^2–^ moiety, rendering the metal centers equivalent. The ^1^H NMR spectrum of **3** in THF-*d*_8_ exhibits a set of two triplets of doublets at 6.61 and 6.14 ppm with coupling constants ^3^*J*_H–H_ of 3.80 and 7.13 Hz that are attributed to the aromatic protons of the bridging diamagnetic flv^2–^ ligand (Figures S5 and S6).

Electrochemical studies were performed on **3** to assess the chemical accessibility of a flv^1–•^ radical-bridged complex via oxidative means. Transferring the analogous conditions employed for **1** which are THF solution and a (^*n*^Bu_4_N)PF_6_ supporting electrolyte to **3**, lacked features in the cyclic voltammogram, which is ascribed to the poor solubility in THF and/or instability of the generated flv^1–•^ radical under these electrochemical conditions. Conducting the measurements in difluorobenzene with (^*n*^Bu_4_N)PF_6_ as the supporting electrolyte instead, gave one quasi-reversible redox feature at −0.095(7) V (vs Cp_2_Fe^+^/Cp_2_Fe) (Figures 4 and S29). In addition, irreversible oxidation and reduction features were found at +0.36 and −0.81 V, which are reproducible across multiple scans. Two conclusions are drawn from the electrochemical experiments: (1) The probed potential range suggests facile electron removal from the flv^2–^ dianion to a flv^1–•^ radical anion through (electro)chemical oxidation. (2) The irreversible features observed under reductive conditions towards the cathodic limit pointed at the potential attainability of a flv^3–•^ radical trianion. Remarkably, the latter oxidation state is peerless for any ligand of the fluoflavine, aka tetraazatetracene family.

**Figure 5:**
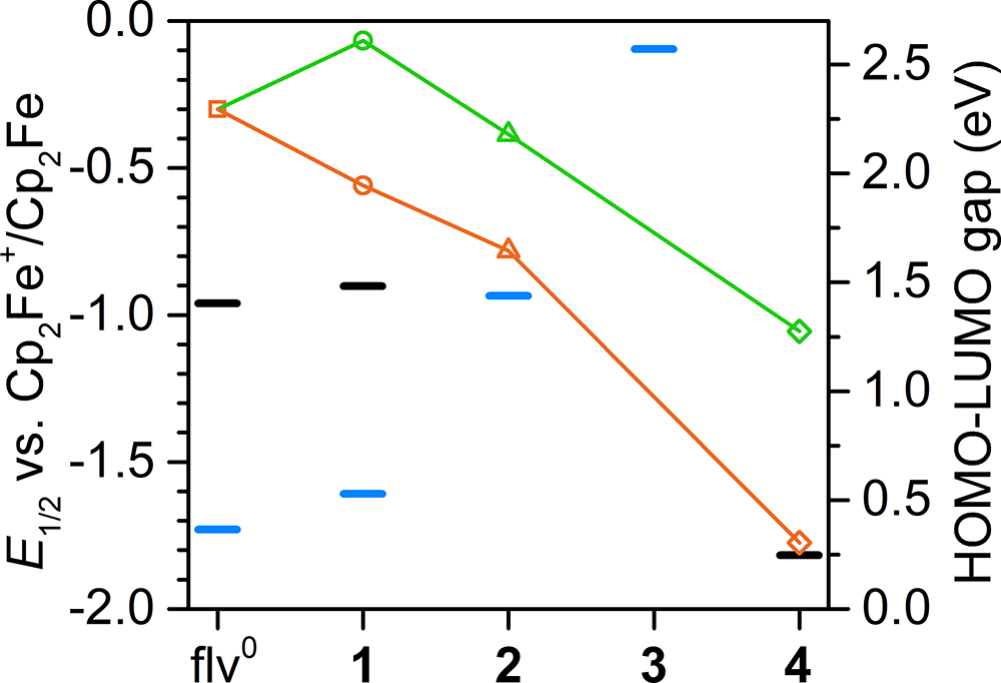
Schematic representation of the experimentally determined redox potential for compounds **1**–**4** and the free flv^0^ ligand as solid blue and black bars. Blue bars show the flv^1–•^/flv^2–^ redox processes, whereas the black bars show either flv^0^/flv^1–•^ or flv^2–^/flv^3–•^ redox processes, respectively. Green and orange symbols represent the calculated *α*- and *β*-spin manifold HOMO–LUMO gaps for **1^–^**, **2′**, and **4′**. Green and orange lines are guides for the eye.

**Figure 6:**
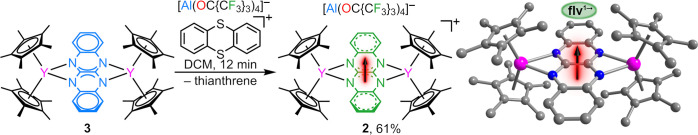
Left: synthesis of [(Cp*_2_Y)_2_(μ-flv^•^)][Al(OC{CF_3_}_3_)_4_] (**2**) through the oxidation of [(Cp*_2_Y)_2_(μ-flv)] (**3**) with thianthrenium [Al(OC{CF_3_}_3_)_4_]. Right: structure of compound **2**. Pink, blue, and gray spheres represent yttrium, nitrogen, and carbon atoms, respectively. All hydrogen atoms, solvent molecules, and the counterion [Al(OC{CF_3_}_3_)_4_]^−^ are omitted for clarity.

Inspired by our electrochemical results of **3**, both the chemical oxidation and reduction of **3** were investigated. First, the oxidation of **3** was carried out with thianthrenium tetra(perfluoro(tert-butoxy))aluminate. Since CV experiments of **3** showed no discernible features in the THF solution, the oxidation was carried out in dichloromethane (DCM). Excitingly, the addition of a thianthrenium solution to in DCM dissolved **3** resulted in an immediate color change from orange to dark green. After removal of presumably unreacted **3** via toluene extraction, the radical-containing complex [(Cp*_2_Y)_2_(μ-flv^•^)][Al(OC{CF_3_}_3_)_4_] (**2**) was crystallized from concentrated DCM solution at −35 °C in 61% yield, constituting the first example of a crystallographically characterized molecular flv^1–•^-radical complex (Figures 6 and S2). The [(Cp*_2_Y)_2_(μ-flv^•^)]^+^ cation crystallizes in the monoclinic space group *I*2/a alongside the [Al(OC{CF_3_}_3_)_4_] counteranion and one DCM molecule and exhibits an inversion center such that the yttrium metal centers are equivalent by symmetry. Notably, compared to the flv^2–^ bearing complex **3**, the one-electron oxidation of the bridging ligand is most prominently expressed through the elongation of Y–N- and Y···Y-distances (Δ = 0.050 and 0.114 Å, respectively), while the central C–C distance is marginally contracted (Δ = −0.021 Å) (Table 1). The elongation can likely be attributed to weaker electrostatic interaction owing to a reduced charge of the bridging ligand. While comparative systems are extremely scarce, such structural trends have been observed for other dinuclear RE metal complexes innate to differing oxidation states of the bridging ligand. For example, radical complexes bearing the 2,3,5,6-tetra(2-pyridyl)pyrazine (tppz) ligand within the [(Cp*_2_Ln)_2_(μ-tppz^1–•^)]^+^ and [(Cp*_2_Ln)_2_(μ-tppz^3–•^)]^−^ complexes exhibit a ∼0.11 Å shorter Ln···Ln-distance with increasing negative tppz charge.^60^ Compared to the free monoanionic flv radical in **1**, the central C–C bond is slightly shorter in **2** (1.467(3) Å (**1**) vs 1.431(6) (**2**)), which likely originates from a reduced charge distribution within the flv^1–•^ ligand due to a coordination to the highly Lewis-acidic yttrium ions (Table S2). The paramagnetic nature of **2** was confirmed by SQUID magnetometry. The experimental room temperature χ_M_*T* value of 0.355 cm^3^ K mol^–1^ (0.1 T) is in excellent agreement with the expected value for an organic radical (*S* = 1/2, 0.375 cm^3^ K mol^–1^, Figures 8, S31–S33, Tables S5 and S6).

**Table 1:** Selected Interatomic Distances (Å) and Angles (deg) of Compounds **1–4**^a^

	**1**	**2**	**3**	**4**
Avg. Y–N		2.437(2)	2.387(2)	2.315(3)
C_2_–C_2′_	1.467(3)	1.431(6)	1.452(4)	1.373(7)
Y–Y		7.144(6)	7.030(1)	6.869(1)
Cnt–Y-Cnt		141.6	141.0	138.2
Ph_Plane1_–Ph_Plane2_	0.1(1)	0.1(1)	0.1(1)	0.1(1)

a A full list of structural parameters are given in Tables S2 and S3. Crystallographic data and structural refinements are listed in Table S1.

**Figure 7:**
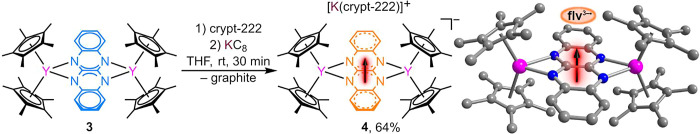
Left: synthesis of [K(crypt-222)][(Cp*_2_Y)_2_(μ-flv^•^)] (**4**) proceeded through reduction of **3** with KC_8_ in the presence of crypt-222. Right: structure of **4**. Pink, blue, and gray spheres represent yttrium, nitrogen, and carbon atoms, respectively. All hydrogen atoms, solvent molecules, and the counterion [K(crypt-222)]^+^ are omitted for clarity.

**Figure 8:**
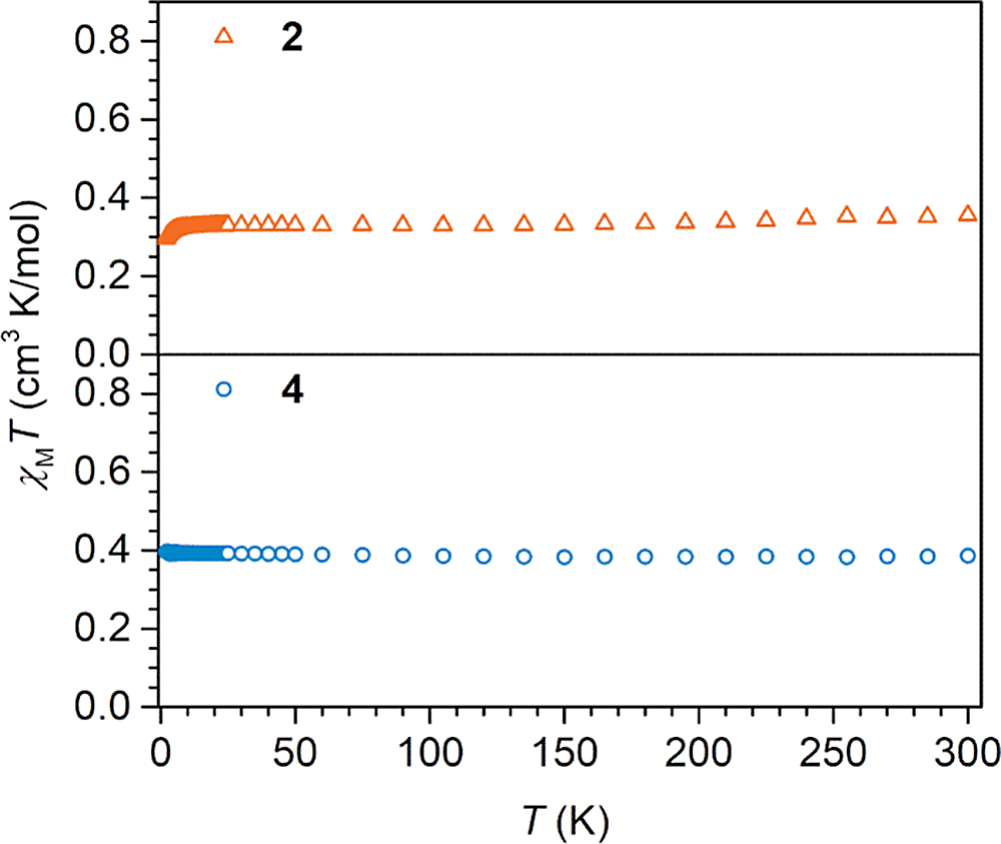
Variable-temperature dc magnetic susceptibility data (*χ*_M_*T* vs *T*) for [(Cp*_2_Y)_2_(μ-flv^•^)][Al(OC{CF_3_}_3_)_4_] (**2**, top) and [K(crypt-222)][(Cp*_2_Y)_2_(μ-flv^•^)] (**4**, bottom), collected from 2 to 300 K under a 0.1 T applied dc field.

**Figure 9:**
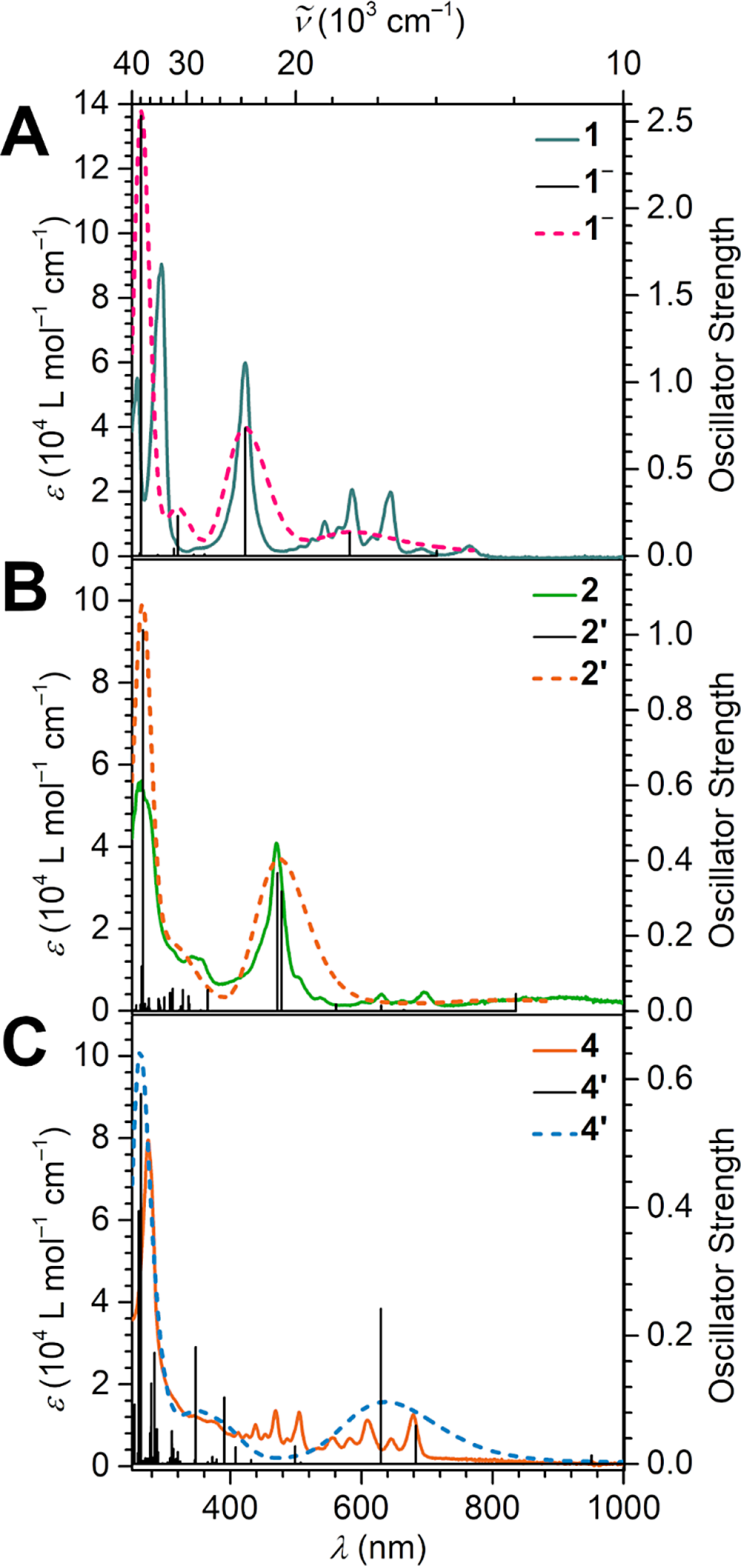
Experimental UV-vis spectra (solid lines) of (**A**) [K(crypt-222)](flv^•^) (**1**), (**B**) [(Cp*_2_Y)_2_(μ-flv^•^)][Al(OC{CF_3_}_3_)_4_] (**2**); (**C**) [K(crypt-222)][(Cp*_2_Y)_2_(μ-flv^•^)] (**4**), and calculated spectra for **1^–^** (pink dashed line), and calculated spectra for the contracted models **2′** and **4′** (orange and blue dashed lines, respectively). Black bars in all plots represent calculated transitions. Concentrations: **1**: 19.29 μmol/L in THF; **2**: 19.95 μmol/L in DCM; and **4**: 14.20 μmol/L in THF.

**Figure 10:**
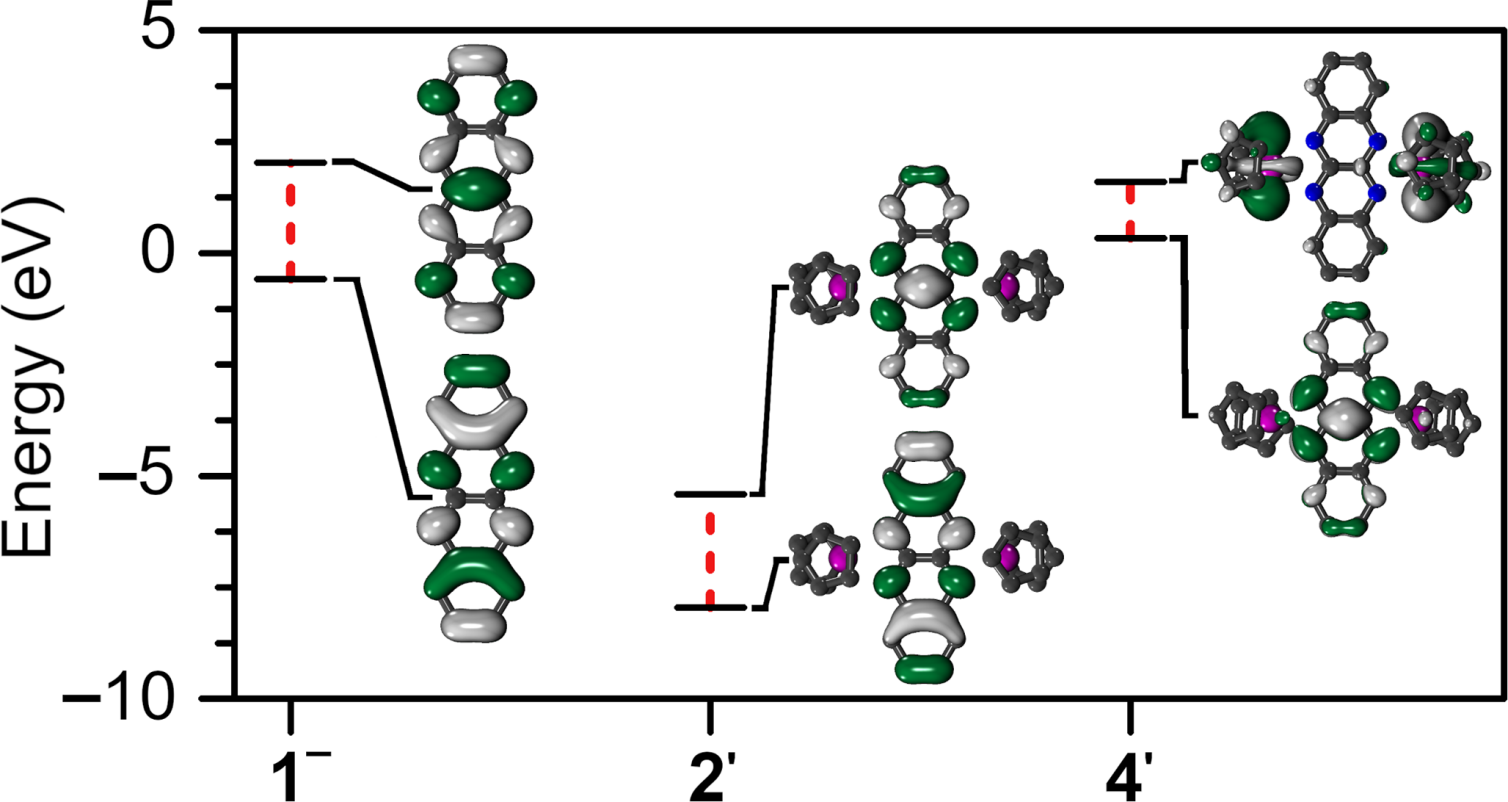
Calculated frontier *α*-spin orbitals and energies of flv^1–•^ (**1^–^**), [(Cp_2_Y)_2_(μ-flv^•^)]^+^ (**2′**), and [(Cp*_2_Y)_2_(μ-flv^•^)]^−^ (**4′**). A full frontier orbital diagram is given in Figure S41.

The crystalline isolation of both the free flv^1–•^ radical anion in **1** and the metal-coordinated version {Y-flv^1–•^-Y} in **2** provided the unique opportunity to scrutinize their spectroscopic properties. This invaluable study augments our understanding of the electronic structure change of organic radicals as a function of their coordination to metal ions. As expected, the UV–vis absorption spectra of **1** and **2** differ vastly: The visible region of the free radical ligand **1** comprises three strong transitions at 544, 586, and 644 nm, as well as two weak absorptions at 691 and 765 nm, and a sharp band at 423 nm. The latter could be assigned to a π → π* transition into a singly occupied molecular orbital (SOMO) via time-dependent density functional theory (TDDFT) calculations on the contracted model of **2**, **2′**, where all Cp* methyl groups are substituted by hydrogen atoms (Figures 9, S19, S20, and S23, Tables S29 and S30). By contrast, the bands between 500 and 800 nm in **2** experience a red shift and a decrease in overall intensity, and the π → π* transition into the SOMO is shifted to 470 nm. These shifts correlate with a reduced energy difference between the highest occupied and lowest unoccupied orbitals (HOMO and LUMO, respectively), where both *α*- and *β*-spin manifolds are substantially stabilized in the metal-coordinated radical versus the free radical (Figures 5, 10, and S41). The smaller HOMO–LUMO gaps in **2′** suggest further electron uptake to be readily feasible, which would lead to higher negatively charged flv oxidation states.

An inspection of the cyclic voltammograms of **1** and **2** uncovered a considerable shift in *E*_1/2_ of the flv^1–•^ → flv^2–^ process (−1.608(3) V (**1**) and –0.935(2) V (**2**), vs Cp_2_Fe^+^/Cp_2_Fe) (Figures 4, S26, and S28). Such anodic shifts in reduction potential may be traced back to decreased HOMO–LUMO gaps arising from a ligation to Lewis-acidic yttrium ions (Figures 5, 10, and S41). This may be a powerful yet generally applicable demonstration that a metal coordination to a potentially redox-active ligand provides access to hitherto unknown radical oxidation states. Notably, when scanning toward negative potentials beyond −2.0 V, no further reversible redox features were monitored for **2**. By contrast, scans toward the anodic limit revealed a strongly irreversible feature and a loss of the reversible flv^1–•^ → flv^2–^ signal (Figure S28). This suggests that the oxidation of flv^1–•^ to a targeted flv^0^ neutral compound instead gives rise to highly reactive species, potentially decomposing to release free flv^0^.

Delighted by the isolation of archetypical flv radical-bridged complex **2**, we tackled the synthetic challenge of devising a path to the yet unknown flv radical oxidation state 3–•. Our approach encompassed the chemical reduction of the flv^2–^-bridged complex **3** with one equivalent of KC_8_ in the presence of chelating crypt-222. Excitingly, the addition of the reducing agent caused an instant color change from bright yellow to dark green, followed by a gradual turn to dark blue as the final color that ultimately led to the isolation of [K(crypt-222)][(Cp*_2_Y)_2_(μ-flv^•^)], **4** (Figure 7). Dark blue single crystals of **4** were grown from a concentrated THF solution at –30 °C over the course of 3 days in 64% yield. Complex **4** constitutes the first isolated system of any kind that contains a fluoflavine radical trianion, flv^3–•^.

The metal centers in **4** are inequivalent, reminiscent of an asymmetric unit containing two halves of the complex unit. **4** crystallizes in the triclinic space group *P*1̅ alongside the [K(crypt-222)]^+^ countercation and four THF molecules (Figure S4). Comparing the bond metrics of **3** and **4**, the change in the oxidation state of flv is most prominently reflected in the core of the ligand: The central C–C, C–N, and Y–N bonds and the Y···Y distance are significantly decreased by 0.079, 0.047, 0.072, and 0.161 Å, respectively (Tables 1, S2 and S3). The DFT calculations performed (see below) ascribe these structural differences to the population of the SOMO of **4′**, which is predominantly located on the flv ligand (Figure 10). An akin tendency of bond distance shrinkage at the heart of the bridge also occurred when [K(crypt-222)][(Cp*_2_Y)_2_(μ-Bbim^•^)] containing a radical trianion formed upon reduction of the parent complex bearing a nonradical bisbenzimidazole dianion.^13^ The inner-ligand contraction additionally engenders enhanced steric repulsion between the {Cp*_2_Y}^+^ units. This causes a slight increase of the N–Y–N bite angle from 57.6(1)° in **3** to 60.4(1)° in **4** and results in a small out-of-phase displacement of the Y ions relative to the bridging flv ligand (Y–flv–Y: 16.3(3)° (**3**) and 13.1(5)°/20.3(5)° (**4**)).

As the complexes **2**, **3**, and **4** contain the fluoflavine ligand in the oxidation states −1, −2, and −3, respectively, this series represents an extremely rare example for isolable ligand-based multivalence. Thus, the comparison of structural parameters across the three flv complexes is of great interest. In fact, such wealth of accessible oxidation states is considered a feat for transition metal chemistry, and ligand-based redox activity has been proposed as a promising substitute for expensive metal-based catalysts.^61,62^

The successive increase of negative charge traversing from **2** to **4** is structurally best reflected in the gradual contraction of the Y–N and Y···Y distances by 0.122 and 0.275 Å, respectively. Specifically, the contraction of the Y···Y distance in **4** is attributed to both the increased electrostatic interaction owing to the higher negative charge of the bridge and a pronounced out-of-plane displacement of the Y atoms with respect to the flv plane that thus forms a zigzag conformation. By contrast, the Y–N distance contracts steadily by ∼0.06 Å per additional negative charge. Notably, the inner-flv bonds experience a nonlinear change in interatomic distances: The central C–C bond is elongated by 0.021 Å when **2** is chemically reduced to **3**, but considerably contracted by 0.079 Å upon further reduction to **4**. Such deformations reflect the differences in frontier orbital geometries, where the SOMO in **2′** comprises nitrogen *p*_z_-orbitals of opposing sign per each quinoxaline unit and no contribution of the central C–C bond (Figure 10). In contrast, the SOMO in **4′** is composed of nitrogen *p*_z_-orbitals that exhibit the same sign, with substantial contribution of the central C–C bond.

The oxidation state change was also monitored in the corresponding UV–vis absorption spectra for **3** and **4** (Figure 9). Both compounds feature strong absorptions around 270, 440, 470, and 505 nm; however, only **4** shows absorptions between 523 and 700 nm (Figures 9, S20–S23, Tables S30 and S31). For the free flv ligand, a sharp, intense absorption at ∼280 nm and a broad absorption feature between 360 and 440 nm (in acetonitrile) were reported.^59^ Taking this into account, the sharp features for **3** and **4** at 440, 470, and 505 nm are attributed to metal–to–ligand charge transfer transitions. This is further supported by TDDFT-predicted transitions for **4′**, most prominently for a transition around 499 nm, which can be ascribed to a flv(π) → Y(*d*)* transition (Table S31). Furthermore, the transitions of **4** between 600 and 700 nm match well with two predicted transitions that could be assigned to a stronger HOMO → SOMO π → π* transition and a weaker π → π* transition from the SOMO to a vacant excited π* orbital. Relative to the flv^1–•^-containing complex **2**, the most noticeable difference is the absence of the strong band at 423 nm, which corresponds to the π → π* transition into the SOMO of **2′** but is inaccessible in **4′** due to double occupation of the respective MO (Figure 10, Table S30). Noteworthy, a transition involving the same ligand MO geometry is identified for all three radical compounds **1**^**–**^, **2′**, and **4′**: For **1**^**–**^ and **2′**, this represents a SOMO to LUMO transition, whereas for **4′** this is the highest doubly occupied MO to SOMO transition. It is predicted to occur at 629 nm for both **2′** and **4′**, but considerably more intense in **4′**.

The structural changes arising from the coordination of **1**–**2,** and a formal two-electron reduction of **2**–**4** are also mirrored in the respective IR spectra (Figures S14 and S15): For **1**, among multiple vibrational bands, the emergence of a strong vibrational band at 1517 cm^–1^ is found that is reproduced well via a calculated vibrational mode corresponding to an in-plane wagging vibration involving the peripheral flv Ph rings (Figure S16). Upon coordination of the flv^1–•^ ligand to two yttrium atoms, this vibration experiences a blue shift by 16 cm^–1^ from 1517 (**1^–^**) to 1533 cm^–1^ (**2′**) (Figure S17). The same vibration experiences a further blue shift by 17 cm^–1^ to 1550 cm^–1^ (**4′**) (Figure S18). Both shifts are in excellent agreement with the strengthening of the central C–C bond upon coordination and reduction, as confirmed via DFT calculations below. Comparing both radical-bridged complexes, a strong vibrational band at 1338 cm^–1^ stands out, which matches well with the vibrational mode calculated at 1347 cm^–1^ for **4′**. This calculated vibration comprises a combination of longitudinal in-plane fluoflavine deformations, most prominently an antisymmetric wagging of the central C–C bond (Figures S17 and S18). A comparable vibration is calculated for **2′** at lower energies (1317 cm^–1^). The appearance of this higher-energy vibration in **4′** is again in excellent agreement with the crystallographically observed contraction, which ultimately strengthens the bond.

Similar to the case of **1** and **2**, the poor solubility and paramagnetic nature of **4** precluded further spectroscopic insight via ^1^H NMR spectroscopy. The room temperature *χ*_M_*T* value of 0.386 cm^3^ K mol^–1^ (0.1 T), determined via SQUID magnetometry, is close to the expected value for an organic radical with one unpaired electron (Figures 8, S34–S36, Tables S7 and S8).

### Variable-Temperature EPR Spectroscopy

The electronic structure and spin density of the new radical complexes **2** and **4**, bearing the flv^1–•^ and flv^3–•^ radical anions, were explored through a combination of variable-temperature continuous wave (cw) EPR spectroscopy and DFT calculations. In EPR spectroscopy, the intensity of a signal for an *S* = 1/2 spin system depends on the population difference between the spin ground state and the excited state, which are degenerate under ambient conditions but are split under the application of an external magnetic field due to the Zeeman effect.^100^ The EPR signals are then split due to hyperfine coupling (HFC) of the unpaired electron with spin-active nuclei (*I* > 0), such as ^1^H (*I* = 1/2) and ^14^N (*I* = 1). The magnitude of this coupling correlates with the spin density at the respective nucleus and can be used to map the electron density within an organic radical.^24^ For appealing intrinsic magnetic properties to emerge in polynuclear lanthanide complexes, such as those leading to slow magnetic relaxation indicative of SMM behavior, a substantial delocalization of the spin density on the radical ligand is required to promote strong magnetic exchange coupling involving paramagnetic metal ions. Thus, determining HFC constants through EPR techniques aids in quantifying the degree of electron density delocalization for a given paramagnetic ligand.

**Figure 11:**
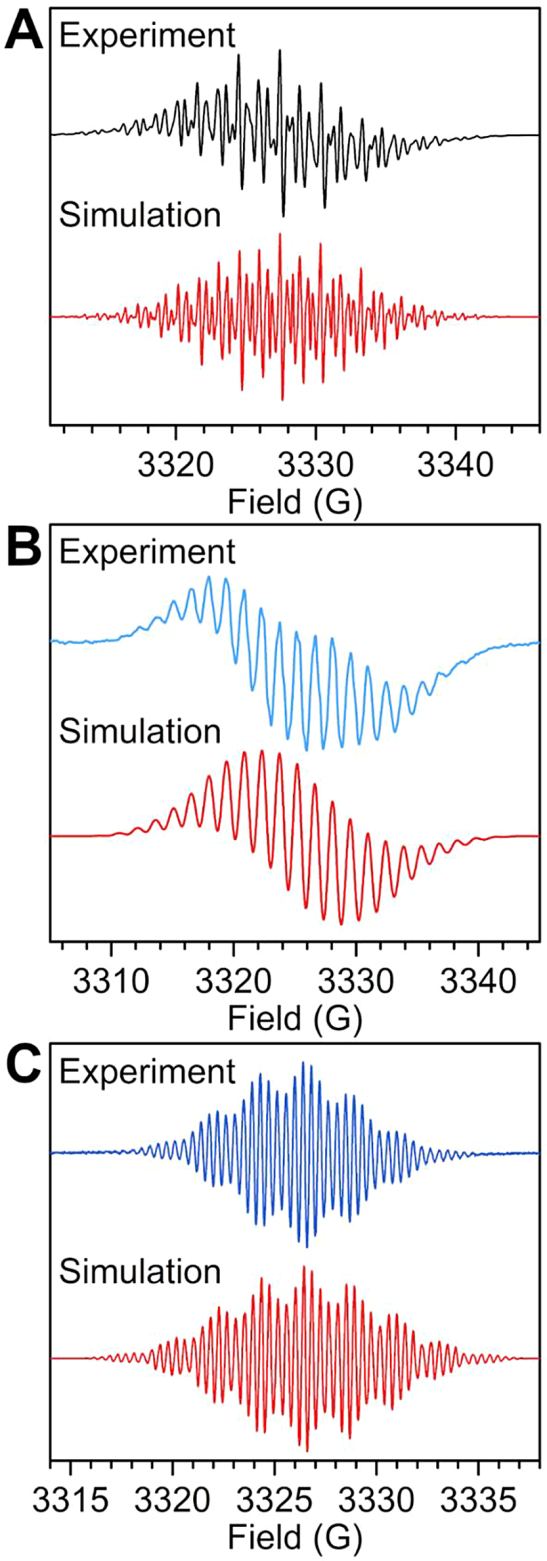
Experimental (black and blue) and simulated (red lines) cw-EPR spectra of (A) [K(crypt-222)](flv^•^) (**1**); (B) [(Cp*_2_Y)_2_(μ-flv^•^)][Al(OC{CF_3_}_3_)_4_] (**2**); (C) [K(crypt-222)][(Cp*_2_Y)_2_(μ-flv^•^)] (**4**), collected on 0.88 and 0.22 mmol/L THF (**1** and **4**) and 0.67 mmol/L 1,2-difluorobenzene (**2**) solutions at room temperature. Simulation parameters are given in Table 2.

**Figure 12:**
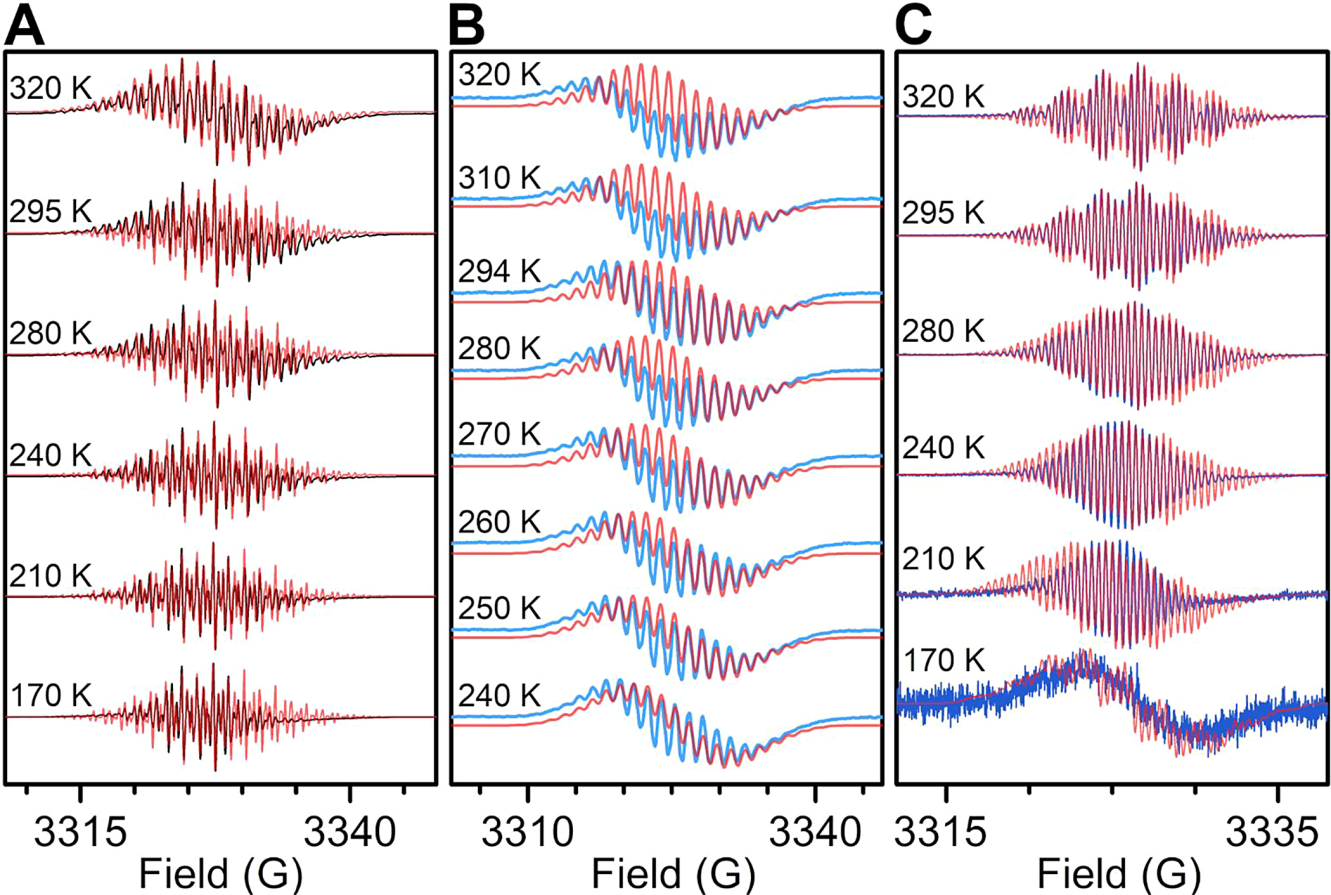
Experimental (black and blue) and simulated (red) normalized VT-EPR spectra of (**A**) [K(crypt-222)](flv^•^) (**1**); (**B**) [(Cp*_2_Y)_2_(μ-flv^•^)][Al(OC{CF_3_}_3_)_4_] (**2**); (**C**) [K(crypt-222)][(Cp*_2_Y)_2_(μ-flv^•^)] (**4**), collected on 0.88 and 0.22 mmol/L THF (**1** and **4**) or 0.67 mmol/L difluorobenzene (**2**) solutions between 170 and 320 K (**1** and **4**) or 240 and 320 K (**2**). Simulation parameters are given in Table 2. Absolute intensity values were obtained via double integration and normalization against stable TEMPONE samples in Figures S7–S11 and plotted in Figures S12 and S13.

Room temperature and variable temperature X-Band EPR data of **1**, **2**, and **4** were collected by using ∼0.5 mmol/L solutions in THF (**1** and **4**) and difluorobenzene (**2**), respectively (Figures 11 and 12). The spectra reveal rich fine structure due to coupling involving ^89^Y, ^14^N, and ^1^H nuclei. The spectrum of **1** was satisfactorily simulated via the EasySpin package^63^ for MATLAB by employing HFC constants, *A*, for the four ^14^N donor atoms of 8.10 MHz, 4.25 MHz for the four innermost protons, and 2.50 MHz for the four outer protons with an isotropic *g*-value of 2.00355 (Table 2). These values show a pronounced concentration of spin density on the ^14^N nuclei with considerable delocalization onto the peripheral phenyl rings. The unnormalized intensity of the EPR signal gradually declines with decreasing temperature, possibly indicating the formation of diamagnetic π-dimers in solution (Figure 12). However, a corrected intensity can be obtained by dividing the experimental double integration of **1** through the double integration of a reference TEMPONE sample, which reveals that the intensity is unchanged within the probed temperature range and thus signifies an absence of π-dimerization (Figures S7–S9 and S12). The complexation of the free flv^1–•^ radical anion to two yttrium ions causes massive changes in the EPR spectrum. While the spectrum of **1** featured a narrow line width that allowed for the accurate simulation of even the minor HFCs, the line width attained for **2** is considerably larger, which did not improve upon cooling or heating the sample, rendering its simulation challenging without further knowledge of the spin density. Such broad lines may originate from multiple factors, such as (a) the presence of multiple, very similar HFCs, (b) incomplete averaging of anisotropic *A* contributions due to slow tumbling of the analyte in solution, or (c) fast relaxation times.^100^ Hence, the EPR parameters of **2** were calculated through DFT methods, which uncovered significant HFCs to the ^89^Y nuclei (Tables 2, S25 and S26). With this insight, a successful simulation of the room temperature EPR spectrum of **2** was possible with 7.90 MHz (^14^N), 3.90 MHz (^89^Y), 4.50 MHz (^1^H), and 1.30 MHz (^1^H) (Figure 11). This confirms that the spin density of the flv^1–•^ radical is mostly depleted at the peripheral Ph positions, and substantial spin density is transferred onto the yttrium centers. Notably, the normalized EPR intensity profile remains essentially unchanged prior to versus after metal coordination, akin to isolated paramagnetic species in solution (Figures S10 and S13). Such spin density delocalization from the ligand-based radical onto metal centers is a prerequisite for enticing properties, such as strong direct magnetic coupling or conductivity.

**Table 2:** Overview of EPR Parameters Discussed in the Main Text^a^^,^^b^

**Nucleus**	**Number**	***A*_iso_ (sim) (MHz)**			***A*_iso_ (DFT) (MHz)**			**Mulliken spin population**		
		**1**	**2**	**4**	**1^–^**	**2′**	**4′**	**1^–^**	**2′**	**4′**
**^89^Y**	2		3.90	1.00		3.38	1.07		–0.0094	0.039
**^14^N**	4	8.10	7.90	5.85	103.9	–7.59	5.34	0.18	0.16	0.12
**^1^H (inner)**	4	4.25	4.50	2.25	–3.89	–4.62	–2.29	–0.0034	–0.00076	–0.0017
**^1^H (terminal)**	4	2.50	1.30	1.00	–2.55	–0.72	–0.99	–0.0027	–0.0037	–0.00094

a *g*_iso_: 2.00355 (**1**), 2.003175 (**2**), 2.0049 (**4**). Peak-to-peak separation: 0.033 mT (**1**), Gaussian line width: 0.08 mT (**2**), 0.03 mT (**4**).

b Simulated experimental and DFT-calculated hyperfine coupling constants *A*_iso_, and calculated Mulliken spin populations for compounds **1^–^**, **2′**, and **4′**. Simulated values for **1**, **2**, and **4** were obtained from experimental spectra taken at room temperature. Details on DFT-calculated HFCs are listed in Tables S25–S27.

To assess the degree of spin density transfer from the flv ligand onto the yttrium ions as a function of charge, EPR data were collected for **4** (Figure 12). The two-electron reduction of **2**–**4** alters the EPR spectrum tremendously in that the signals are grouped into a set of nine primary signals, which are further split into five sub-signals, giving rise to a set of 45 lines. Such splittings hint at spin density accumulation at the ^14^N atoms and one dominant ^1^H species. A satisfactory simulation of the EPR spectra for **4** was reached using 5.85 MHz (^14^N), 1.0 MHz (^89^Y), 2.25 MHz (^1^H, inner), and 1.0 MHz (^1^H, terminal), which is in excellent agreement with the EPR parameters obtained through DFT (Table S27). Remarkably, both the ^14^N and ^89^Y HFCs in **4** are much smaller than in **2**, which may seem counterintuitive since a higher charge is generally associated with a more diffuse electron cloud and thus raised spin density transfer. However, the calculated Mulliken spin populations reveal a ∼4.1-fold larger spin population at the yttrium centers in **4′** relative to **2′** (Table 2).

The delocalization can also be estimated as the ratio of |*s*_M_(^89^Y)|/|*s*_M_(^15^N)|, where *s*_M_ are the Mulliken spin populations at the ^89^Y and ^15^N nuclei, respectively. The determined ratios for **2′** and **4′** exhibit a ∼5.6-fold increase as a result of the formal two-electron reduction of flv^1–•^ → flv^3–•^. Accordingly, the spin density transfer from the ligand-based radical onto the metal centers seems far more efficient in **4′** than in **2′** when taking into account both the absolute and relative spin populations. Such metal–ligand interactions are pivotal for strong magnetic exchange coupling with metal ions and lanthanide ions, in particular, as their valence 4*f*-orbitals are deeply buried.

Like for **2**, the normalized EPR intensity profile for **4** is reminiscent of noninteracting paramagnetic molecules in solution, exhibiting only minor changes across the probed temperature range (Figures S11 and S13).

Notably, we isolated previously the bisbenzimidazolyl radical-bridged complex [K(crypt-222)][(Cp*_2_Y)_2_(μ-Bbim^•^)] that is structurally related to **4** with the same metallocenium cations and a similar tetradentate bridge composed of the same atoms but differing in the alignment of the central C–C bond (see Figures S39 and S40).^13^ This provides the unique opportunity to analyze the influence of coordination geometry on the spin density distribution and transfer onto metal centers in radical complexes containing an isomeric bridge. The simulation of the VT-EPR spectra of the Bbim^3–•^ complex amounted to ^14^N and ^89^Y HFCs of 5.1 and 0.42 MHz,^13^ which signify that the spin density delocalization in **4** is larger. Notably, the DFT calculated HFCs for the Bbim complex suggest a more substantial yttrium contribution than that for **4**′**** (Tables S27 and S28). This can be ascribed to variations in the coordination geometry or to conformational changes arising from much longer Y···Y distances of 6.869(1) Å in **4** relative to 6.030(1) Å in [(Cp*_2_Y)_2_(μ-Bbim^•^)]^−^. This causes a lesser out-of-plane displacement of the Y atoms in **4** (average Y–N–N–Y angle: 16.7(5)° vs 23.3(2)° in [(Cp*_2_Y)_2_(μ-Bbim^•^)]^−^), giving rise to a less tilted flv bridge with respect to the Y–Y plane (Tables S14 and S15).

### Effect of Charge on Electronic Structure

Electronic structure analyses of RE complexes comprising paramagnetic ligands through computational means are extremely rare, which is largely attributed to the challenging synthesis of such elusive compounds. In addition, computations are substantially more difficult owing to the presence of unpaired electrons. In fact, there are no in-depth computational studies on radical-bridged dinuclear RE complexes known to have radicals in two differing oxidation states. Excitingly, the presented computations on **2′** and **4′**, constituting contracted model complexes of **2** and **4**, herein represent the first thorough study of its kind and give insight into the electronic structure. Such information is critical for improved material design.

These three aspects of chemical bonding are most relevant: (1) Y–N covalency, (2) flv aromaticity, and (3) spin density transfer from the paramagnetic ligand onto the Y centers. Hence, the electronic structure and spin density of **2′** and **4′** were calculated via DFT and studied via natural localized molecular orbital (NLMO) and quantum theory of atoms in molecules (QTAIM) analyses on optimized structures (Figures S37–S39, Tables S9–S13). All calculations and NLMO analysis were carried out with the Orca 5.0.3 program suite^64,65^ and NBO 7.0.^63^ QTAIM analysis was conducted with the MultiWfn program.^66^

The total electron density of an open shell molecule within the framework of unrestricted density functional theory (UDFT) at a point *r* is described as the sum of electron densities of the spin up (or *α*) and the spin down (or *β*) manifold, eq 1.
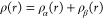
1where *ρ*_α_(*r*) and *ρ*_β_(*r*) are the spin densities of the *α*- and *β*-electron manifolds, respectively. *ρ*(*r*) is comprised of all occupied and unoccupied canonical orbitals, which are delocalized over the entire molecule, rendering an interpretation via “conventional” chemical understanding difficult. A clearer picture is provided through localization schemes such as the Natural Bonding Orbital (NBO) and NLMO analyses.^63,67^

First, the Y–N covalency was probed through inspection of the NLMOs describing the metal–ligand interaction (Tables S16–S21). **2′** and **4**′**** lack bonding NLMOs, suggestive of strongly ionic bonding, which is expected for organometallic RE complexes. Each nitrogen atom of the flv ligand exhibits lone pairs (LPs) with contributions of the coordinated Y atom, indicating a small degree of covalency. These contributions resemble ∼5.0% for **2**′**** and ∼6.5% for **4**′****. Although an akin trend is anticipated from an electrostatic point of view, the discrepancy is too small to justify a covalency increase due to enhanced overlap between radical and metal orbitals. Intriguingly, **2**′**** features an equal number of LPs for *α*- and *β* spin manifolds, whereas **4**′**** exhibits twice as many *α* spin N LPs, whereof the second LP possesses a larger ionic character (∼2% Y contribution). Though remarkable, the experimental differences in spin density transfer are not interlinked to Y–N covalency. Noteworthy, the N LP contributions in [(Cp_2_Y)_2_(μ-Bbim^•^)]^−^ are identical to **4**′****, which excludes covalency changes to originate from ligand geometry (Tables S22–S24). The findings alluded to the insensitivity of NLMOs toward subtle electronic structure variations causing dramatic changes in EPR spectra, prompting us to examine the bonding in **2**′**** and **4**′**** via QTAIM analysis (Figures S42–S44, Tables S32–S34).

In QTAIM, the topology of the electron density *ρ*(*r*) is examined through its first derivative ∇*ρ*(*r*), and points where ∇*ρ*(*r*) = 0⃗ are described as critical points *r*_c_.^68^ These points reside primarily between two neighboring atoms and in the center of carbocycles, called bond-critical points (BCPs) and ring-critical points (RCPs), respectively.

Real space values at BCPs are distinctive for certain types of bonding, most importantly, the total energy density (*H*), which is obtained through *H* = *G* + *V*, where *G* is the electronic kinetic energy and *V* is the potential energy. For positive *H* at a BCP (for example, when *G* > −*V*), the accumulation of electrons is destabilizing, as opposed to a negative *H* (*H* < 0), which signifies a stabilization. Thus, the sign of *H* classifies an interaction as either electrostatic dominant (*H* > 0) or covalent dominant (*H* < 0).^68^

Besides *H*, the additional descriptors ellipticity of the electron density (*ε*) and the Mayer Bond Order (MBO) are relevant to pinpoint the bond order through QTAIM. *ε* adopts values close to 0 for single bonds and larger values for higher bond orders such as 0.23 for aromatic bonds or 0.45 for the double bond in ethylene.^68^

All descriptors affirm substantially increased bond strength and order for the Y–N bonds in **4**′**** compared to **2**′****: *H* possesses small, negative values for both compounds, indicative of a weakly covalent interaction (Tables 3, S33 and S34). The two-electron reduction of **2**′**** to **4**′**** engenders a considerably more negative *H*, suggestive of increased covalency in **4**′**** (*H* = −0.004 (**2**′****),–0.009 (**4**′****)) and therefore, illustrates stronger Y–N bonding in **4**′****. The augmented covalency is also reflected in a 2.4-fold growth in *ε* values and a 1.5-fold increase in MBO values going from **2**′**** to **4**′****, where higher bond orders are ascribed to **4**′****. Compared to the Y–N MBO of 0.57 found for [(Cp_2_Y)_2_(μ-Bbim^•^)]^−^, **4**′**** exhibits a marginally larger value in accordance with slightly increased covalency (Figure S46, Table S36). The stronger Y–N interaction also has ramifications for the ligand itself. The two-electron uptake of flv^1–•^ to give flv^3–•^ alters the descriptors of the inner-flv BCPs. For instance, the central C–C bond exhibits an increase in *H* by 16% for **4**′**** vs **2**′****, while the *ε* value of **4**′**** is almost twice as large as in **2**′****. Similarly, the MBO value of 1.28 in **2**′**** vs 1.46 in **4**′**** implies a considerably higher bond order.

**Table 3:** Summary of QTAIM Bond Order Parameters for Complexes **2′** and **4′**^a^

	***ε***		**MBO**	
	**2′**	**4′**	**2′**	**4′**
**Central C–C**	0.17	0.31	1.28	1.46
**Lateral C–C**	0.19	0.21	1.28	1.31
**Y–N**	0.055	0.13	0.41	0.62

a The bond ellipticity *ε* and Mayer Bond Order (MBO) adopt larger values with increasing bond orders. See Tables S33 and S34 for detailed description for each critical point.

Besides varying degrees of Y–N covalency, the coordination of the flv radicals to metal ions in **2**′**** and **4**′**** results in a higher electron delocalization within the flv ligand. The central C–C BCP in **1^–^** exhibits much smaller *H* values, as well as 12% smaller *ε* and MBO values (Figure S42, Tables 3 and S32). These changes are less distinct for the terminal Ph rings, as exemplified by the identical MBO of 1.27 for **1^–^** and **2**′****, whereas for **4**′**** the MBO value of 1.31 is almost equivalent to the central C–C bond. Excitingly, the foregoing results mark the first DFT study of metal coordination to a paramagnetic ligand and the associated impact on its electronic structure.

QTAIM constitutes a measure to quantify the aromaticity of a given ring system by inspection of the real space values at RCPs. To this end, the Shannon aromaticity index (SAI) is employed. The aromaticity boosts with decreasing SAIs, where the cutoff between aromaticity and antiaromaticity is 30 < SAI < 50 (×10^–4^).^69^ Although useful to describe organic aromatic systems,^70^ we found the curvature of the electron density (λ_π3_) to experience only marginal changes upon oxidation state change of the flv ligand and metal coordination; hence, we focus on SAI as a more sensitive aromaticity descriptor in the following.

First, we calculated the neutral flv^0^ as a reference system and plotted the SAI value for each ring (Figures S45 and S47, Table S35). The large disparity in SAI values implies that the terminal rings are more aromatic than the inner rings (SAI: 36.2 and 16.6; for the central and terminal rings, respectively). This trend remains for **1^–^** containing the free flv^1–•^ radical, with a strong increase in aromaticity for the terminal rings (SAI: 33.1 and 5.08). In **2′**, the MBO of the lateral C–C bonds uncovers that the complexation of the flv^1–•^ radical augments electron delocalization, as indicated by much smaller SAI values (SAI: 17.6 and 5.6). The delocalization is even stronger in complex **4**′**** innate to the more reduced flv^3–•^ radical, with almost identical metrics for the central and terminal rings (SAI: 4.0 and 1.9). Taken together, both the coordination and reduction of flv radicals result in a significant amplification of aromaticity in the flv scaffold according to the Shannon aromaticity index, where the largest aromaticity is predicted for the flv^3–•^ radical-containing complex **4**′****.

### Effect of Charge on Spin Density Distribution

The metal coordination and reduction of the ligand have profound implications for the electronic structure of the flv radicals, but the differences in HFCs obtained through EPR spectroscopy cannot originate from aromaticity/covalency changes alone. Therefore, we probed the spin density quantitatively to determine the spin density transfer mechanism present in each part of the molecule. In general, the topological analysis of spin density is carried out analogously to the electron density, which is the function investigated for QTAIM. However, despite the mathematical equalities, most studies examine the topology of the electron density over the spin density. In fact, the latter scrutiny is scarce, with only a few organic radicals and even fewer paramagnetic metal complexes explored.

In principle, there are two mechanisms that describe how spin density is placed by a given *α*-unpaired electron onto other atoms or its own atomic orbitals.^72^ The first is spin delocalization: Spin density distribution across a molecule arises from the individual atomic orbitals comprising the SOMO. The second is spin polarization: The presence of an *α*-spin introduces the spin density of the opposing *β* sign at atoms bonded to it. To minimize electronic repulsion between same-spin electrons, the *α* electron of a bonding electron pair will stay closer to its atom, thus prompting excess positive spin, whereas the corresponding *β* electron will *β*-polarize neighboring atoms. The competition between both mechanisms determines the sign and magnitude of the spin density for a paramagnetic center.^72^

This is of particular interest in the context of magnetic coupling: The origin of large direct magnetic exchange coupling between paramagnetic transition metal ions and paramagnetic ligands is ascribed to the orbital overlap between the bonding *d*-orbitals and the ligands SOMO.^71^ By contrast, the mechanism for RE complexes is far less clear-cut, since direct orbital overlap is extremely weak owing to the deeply buried 4*f* orbitals. Here, we present the first comprehensive study of the impact of RE metal coordination and radical oxidation state, considering two distinct ones, on the spin density.

Hence, to gain valuable insight into the magnetic structures of **1^–^**, **2′**, and **4′**, we explored the spin densities via topological means. As quantitative studies of the spin density are rare, we chose to briefly outline the underlying fundamentals here. A comprehensive review of topology analysis on spin density has been reported by Bruno et al.^72^ The spin density for a given paramagnetic molecule at a point *r* is expressed by eq 2.
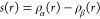
2

The Laplacian (or the second derivative) of the spin density is consequently obtained through eq 3,
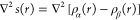
3where ∇^2^*ρ*_α/β_(*r*) is the Laplacian of the *α*- or *β* spin density, respectively. Comparably to *ρ*(*r*), *s*(*r*) exhibits regions where ∇*s*(*r*) = 0 (the first derivative of the spin density), corresponding to the critical points of the spin density, depicted in the following as *r*_c_. These critical points are utilized to extract real space values of the spin density and thus, deliver insight into the magnetic structure of a given paramagnetic molecule. Here, we confine our analysis to the (3,–3) and (3,+3) critical points of the two central pyrazine rings of the flv ligands, which represent maxima of positive spin density and minima of negative spin density, respectively.

**Figure 13:**
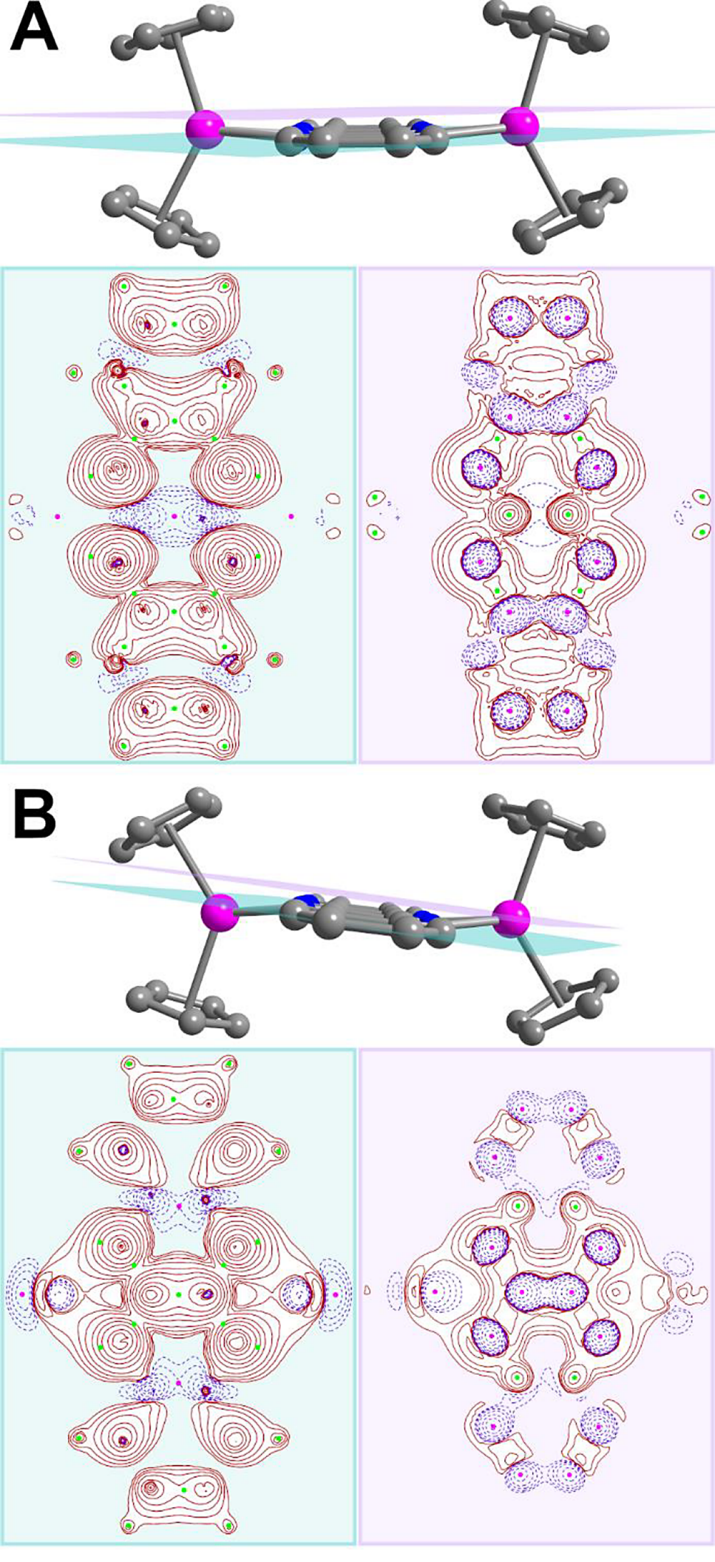
Plots of the Laplacian of the spin density ∇^2^*s*(*r*) of (**A**) [(Cp_2_Y)_2_(μ-flv^•^)]^+^ (**2**′****) and (**B**) [(Cp_2_Y)_2_(μ-flv^•^)]^−^ (**4**′****) along colored planes. Green planes are plotted within the flv plane, and purple planes are plotted through the (3,–3) critical points positioned symmetrically above and below each nitrogen atom. In the plane plots, pink and green spheres represent (3,–3) and (3,+3) critical points (CPs) of the spin density *s*(*r*_c_), respectively. (3,–3) CPs represent maxima of positive spin density and (3,+3) CPs minima of negative spin density. Red solid and purple dashed lines indicate positive and negative eigenvalues of ∇^2^*s*(*r*), where the former reflects spin density localization and the latter spin density delocalization.

The topologies of the spin density for **2**′**** and **4**′**** are shown as plane-plots of ∇^2^*s*(*r*) (Figures 13 and S48). In **1**^**–**^, two spin density maxima are found for each flv nitrogen atom, residing above and below the flv plane, and two maxima are observed for each peripheral Ph carbon atom. The largest *s*(*r*_c_) maxima occur out-of-plane around the central C–C bond with negative spin density. Notably, *s*(*r*) within the flv plane is rather localized, as indicated by the dominantly positive ∇^2^*s*(*r*) values. The flv^1–•^ ∇^2^*s*(*r*) profile remains remarkably unchanged once the radical is coordinated to yttrium (**2′**), displaying negligible overlap with the yttrium atoms, which is suggestive of a spin density localization mainly in the flv ligand. In **2′**, the spin density at the *s*(*r*_c_) maxima residing above and below the nitrogen atoms (positive spin) and central C–C bond (negative spin) is slightly reduced in favor of increased spin density on the peripheral Ph rings. The spin topology changes dramatically after the two-electron chemical reduction, yielding **4**′****, where the nitrogen and central C–C bond spin density maxima exhibit the same sign and are equal in magnitude. **4**′**** is innate to a large overlap of ligand spin density with yttrium metals, both through in-plane and out-of-plane spin densities. The absolute spin density values are larger relative to **2**′****, and the ratio of yttrium to nitrogen *s*(*r*_c_) values is considerably higher in **4**′****, in accordance with the EPR analysis. This additionally highlights the enhanced spin density transfer from the ligand radical to yttrium atoms.

Lastly, the mechanism of spin density transfer from the flv radicals onto the yttrium atoms was elucidated through determining the local spin polarization index (SPI(*r*_c_)) as a measure to compare local and global spin polarization via eq 4 (Tables 4 and S37–S39).^72^
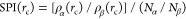
4where *N*_α_ and *N*_β_ constitute the number of *α*- and *β* electrons, respectively. If local spin polarization exceeds the global polarization, meaning SPI > 0.993 in **2′** and **4′**, spin polarization is presumed to be the predominant spin density transfer mechanism. A smaller SPI would indicate spin delocalization. Here, 0.993 represents the SPI value for **2′** and **4′** in the absence of spin polarization (*ρ*_α_(*r*_c_)/*ρ*_β_(*r*_c_) = 1). Notably, the SPI values for **2′** and **4′** are extremely close to the polarization-free value, with values ranging from 0.966 to 1.081 for **2′** and from 0.971 to 1.137 for **4′**, suggesting that the SOMO dominated spin delocalization is far more prevalent than spin polarization in both compounds (Tables S38 and S39). This is also attested by essentially identical SPI values of 0.993 for **2**′ and 1.002 for **4′** for the yttrium centers, further underpinning the absence of local spin polarization. Although small, the SPI values are in accordance with the calculated HFC constants and the spin density topological analysis. Altogether, the findings confirm that spin delocalization and polarization are prevailing in **4′** over **2′**.

**Table 4:** Summarized Topological Analysis of the Spin Density at Critical Points *r*_c_ in **2′** and **4′**^a^^,^^b^

	**2′**						**4′**					
	**(3,–3)**			**(3,+3)**			**(3,–3)**			**(3,+3)**		
	**SPI(*r*_C_)**	***s*(*r*_c_) (E–02)**	**∇^2^*s*(*r*_c_)**	**SPI(*r*_c_)**	***s*(*r*_c_) (E–02)**	**∇^2^*s*(*r*_c_)**	**SPI(*r*_c_)**	***s*(*r*_c_) (E–02)**	**∇^2^*s*(*r*_c_)**	**SPI(*r*_c_)**	***s*(*r*_C_) (E–02)**	**∇^2^*s*(*r*_C_)**
**Y**	0.99	0.0014	–0.0013	0.99	–0.015	0.0017	1.00	0.24	–0.028	0.99	–0.025	0.0098
**N**	1.08	5.7	–1.5	0.99	–0.065	0.024	1.06	4.6	–1.15	0.99	–0.084	0.055
**central** C–C	1.00	0.040	–0.014	0.97	–0.80	0.14	1.14	3.9	–0.69	0.97	–3.6	0.13
**lateral** C–C	1.05	1.4	–0.24	0.99	–1.1	0.036	0.99	0.0028	–0.0020	0.98	–0.22	0.030
**Y–N**	0.99	0.0016	–0.00095				1.01	0.23	–0.021			

a Averaged values are given for spin density critical points *r*_c_. (3,–3) and (3,+3) critical points represent maxima of positive spin density and minima of negative spin density, respectively. Full details of all investigated spin density critical points are given in Tables S37–S39.

b SPI(*r*_c_): Spin polarization index; *s*(*r*_c_): Spin density; ∇^2^*s*(*r*_c_): Laplacian of the spin density; *N*_α_/*N*_β_ = 1.007. SPI < 1 indicate prevalence of the SOMO. Global spin polarization from SOMO_α_–LUMO_β_ difference: 0.93 eV (**2′**), 1.04 eV (**4**′).

For completeness, we quantified the global spin polarization for **2′** and **4′**, which first required the scrutiny of the energy difference between frontier canonical orbitals of the paramagnetic system. In UDFT, the *α*- and *β*-spin manifolds are given the freedom to occupy different spatial regimes, engendering a small energy difference between the SOMO (*α* spin) and the *β* spin equivalent, with which the spin polarization is quantified (Figure S41).^73,74^ The trend that emerged from local spin polarization is the same for the global spin polarization: While a decrease in spin polarization is seen following the coordination of the free radical **1**^–^**** to form **2**′**** (0.95 eV (**1**), 0.86 eV (**2**′****)), a considerable surge is observed for the flv^3–•^-bearing complex (1.05 eV (**4′**)).

Taken together, the spin density topology analysis confirmed the results obtained from the EPR simulations and calculations, enabling a peerless, in-depth understanding of the spin density transfer capabilities of flv radicals in different oxidation states. In conjunction with the electron density scrutiny, the increased covalency and spin delocalization are hypothesized to be the primary driving forces for the spin density transfer in **2**′**** and **4**′****, whereas the additional charge in **4′** augments this further. When compared to the structurally related bisbenzimidazole (Bbim^3–•^) radical-bridged RE complex, the acquired data unfold a similar orbital overlap between the yttrium ions and the corresponding radical bridging ligands, despite the significant structural differences of these two compounds. Thus, the next level in the metal-radical approach toward new RE materials with the potential to revolutionize future technologies is to systematically alter the electronic structure of the radical bridge through chemical substitution, besides the goal to isolate and study new radical complexes, whose substance class remains underexplored due to the synthetic challenge.

## Conclusions

The isolation of a rare series of multiredox organometallic compounds employing the fluoflavine ligand in two radical and one nonradical oxidation state was accomplished, in the form of [(Cp*_2_Y)_2_(μ-flv^•^)][Al(OC{CF_3_}_3_)_4_] (**2**), [(Cp*_2_Y)_2_(μ-flv)] (**3**) and [K(crypt-222)][(Cp*_2_Y)_2_(μ-flv^•^)] (**4**). This series of complexes constitutes an extremely rare example of isolable radical ligands in two different oxidation states for any RE metal and, in fact, the first for yttrium. The successful synthesis of all compounds involved the first implementation of fluoflavine as a ligand platform in RE chemistry. Furthermore, a synthetic route to the free flv^1–•^ in the form of [K(crypt-222)](flv^•^) (**1**) was devised, providing readily access to a bottleable multidentate radical ligand, which is extremely rare and its use will benefit coordination chemistry at large.

The examination of redox processes on the neutral complex (**2**) bearing a flv^2–^ dianion enabled the discoveries of the first fluoflavine flv^1–•^ and flv^3–•^ radical-containing complexes for any metal ion. Their identity was unambiguously proven by single-crystal X-ray diffraction analysis, cyclic voltammetry, UV–vis spectroscopy, and VT EPR spectroscopy. Various DFT techniques aided in elucidating their electronic and magnetic structures.

Importantly, QTAIM analysis revealed a substantial impact of the ligand oxidation state onto the yttrium–nitrogen covalency, and the Shannon aromaticity indices hinted at augmented aromaticity within the flv ligand. The higher spin polarization/spin delocalization confirmed a remarkably increased spin density transfer from the flv^3–•^ radical onto the yttrium center. This is the first report for any metal-radical system in which such a study has been performed. The ramifications are large: The outcome highlights the importance of both increasing the orbital overlap between the radical ligand and metal ions and employing highly reduced radicals to boost the electronic communication between the entities.

The introduction of the flv ligand, including the flv^1–•^ and flv^3–•^ radicals, in lanthanide chemistry may usher in new discoveries around spin-based reactivity and singular optical and magnetic properties. Specifically, merging each radical variant with paramagnetic lanthanides will benefit from the diffuse spin-orbital of the radical ligand and the high magnetic anisotropy of the metal centers, which may pave the way to new high-performance SMMs with real magnetic memory. Another profitable avenue is the design of polymeric coordination assemblies comprising repeating units of metal-coordinated building blocks to achieve unprecedented conductive materials. A third lucrative application bears on the use of redox-active ligands as potential avenues to substitute precious metal catalysts by more economical alternatives. This necessitates, however, accessible and readily switchable oxidation states under ambient conditions which are extremely scarce. Excitingly, the complexes presented herein are accessible in three distinct oxidation states and are potentially applicable in redox-initiated catalysis, which may be probed in the future.

## Supporting Information Available

Detailed synthetic procedures, crystallographic refinement parameters, UV–vis-/NMR-/EPR- and IR spectra, cyclic voltammetry data, magnetic data, computational data, and coordinates of optimized structures (PDF)
