# Supplementary material for: Isolation of Elusive Fluoflavine Radicals in Two Differing Oxidation States
Source: J Am Chem Soc. 2024 Sep 12;146(38):26008–23. doi: 10.1021/jacs.4c05267 (PMC11440492; doi:10.1021/jacs.4c05267)
Supplement: Supplementary file 1 — ja4c05267_si_001.pdf [file ja4c05267_si_001.pdf]

Supporting Information

Isolation of Elusive Fluoflavine Radicals in Two Differing Oxidation States

Florian Benner and Selvan Demir*

Department of Chemistry, Michigan State University, 578 South Shaw Lane, East Lansing, Michigan 48824, USA

*Email: sdemir@chemistry.msu.edu (S.D.)

Table of Contents

1.1 Experimental Methods **S6**

1.1.1 General Information S6

1.1.2 Synthesis of [K(crypt-222)](flv•), (**1**) S6

1.1.3 Synthesis of [(Cp*$_2$Y)$_2$(μ-flv•)][Al(OC{CF$_3$}$_3$)$_4$] (**2**) S6

1.1.4 Synthesis of K$_2$flv S7

1.1.5 Synthesis of [(Cp*$_2$Y)$_2$(μ-flv)] (**3**) via Reduction of flv^0 S7

1.1.6 Synthesis of [(Cp*$_2$Y)$_2$(μ-flv)] (**3**) via Salt Metathesis using K$_2$flv S7

1.1.7 Synthesis of [K(crypt-222)][(Cp*$_2$Y)$_2$(μ-flv•)] (**4**) S8

1.1.8 Single Crystal X-ray Diffraction S8

1.1.9 NMR Spectroscopy S9

1.1.10 EPR Spectroscopy S9

1.1.11 IR Spectroscopy S9

1.1.12 UV-Vis Spectroscopy S9

1.1.13 Cyclic Voltammetry S9

1.1.14 Elemental Analysis S10

1.1.15 Magnetic Susceptibility Measurements S10

1.1.16 Computational Methods S10

1.2 Single Crystal X-ray Diffraction **S12**

Table S1. Crystallographic data and structural refinement of K(crypt-222)(flv•) (**1**), [(Cp*$_2$Y)$_2$(μ-flv•)][Al(OC{CF$_3$}$_3$)$_4$]·CH$_2$Cl$_2$ (**2**·CH$_2$Cl$_2$), [(Cp*$_2$Y)$_2$(μ-flv)]·2THF (**3**·2THF), and [K(crypt-222)][(Cp*$_2$Y)$_2$(μ-flv•)]·4THF (**4**·4THF). S12

Table S2. Selected structural parameters of [K(crypt-222)](flv•) (**1**), [(Cp*$_2$Y)$_2$(μ-flv•)][Al(OC{CF$_3$}$_3$)$_4$]·CH$_2$Cl$_2$ (**2**·CH$_2$Cl$_2$) and [(Cp*$_2$Y)$_2$(μ-flv)]·2THF (**3**·2THF). S13

Table S3. Selected structural parameters of [K(crypt-222)][(Cp*$_2$Y)$_2$(μ-flv•)]·4THF (**4**·4THF). S14

Figure S1. Unit cell of [K(crypt-222)](flv•) (**1**). S15

Figure S2. Unit cell of [(Cp*$_2$Y)$_2$(μ-flv•)][Al(OC{CF$_3$}$_3$)$_4$]·CH$_2$Cl$_2$ (**2**·CH$_2$Cl$_2$). S16

Figure S3. Unit cell of [(Cp*$_2$Y)$_2$(μ-flv)]·2THF (**3**·2THF). S17

Figure S4. Unit cell of [K(crypt-222)][(Cp*$_2$Y)$_2$(μ-flv•)]·4THF (**4**·4THF). S18

1.3 NMR Spectroscopy **S19**

Figure S5. ^{1}H NMR spectrum of [(Cp*$_2$Y)$_2$(μ-flv)] (**3**) (500 MHz, THF-d_8). S19

Figure S6. ^{13}C NMR spectrum of [(Cp*$_2$Y)$_2$(μ-flv)] (**3**) (125 MHz, THF-d_8). S20

1.4 EPR Spectroscopy **S21**

1.4.1 Details for Simulated VT EPR Spectra Depicted in Figure 11 S21

1.4.2 Variable-Temperature EPR Intensity Analysis of Flv Radicals S22

Figure S7. Baseline-corrected EPR spectra collected for the variable-temperature intensity study of [K(crypt-222)](flv•) (**1**) in 1,2-difluorobenzene. S24
Figure S8. Baseline-corrected EPR spectra collected for the variable-temperature intensity study of [K(crypt-222)](flv•) (**1**) in THF. S24
Figure S9. Baseline-corrected EPR spectra collected for the variable-temperature intensity study of [K(crypt-222)](flv•) (**1**) in acetonitrile. S25
Figure S10. Baseline-corrected EPR spectra collected for the variable-temperature intensity study of [(Cp*$_2$Y)$_2$(μ-flv•)][Al(OC{CF$_3$}$_3$)$_4$] (**2**) in 1,2-difluorobenzene. S25
Figure S11. Baseline-corrected EPR spectra collected for the variable-temperature intensity study of [K(crypt-222)][(Cp*$_2$Y)$_2$(μ-flv•)] (**4**) in THF. S26
Figure S12. EPR-integrated intensity as a function of the temperature for [K(crypt-222)](flv•) (**1**) in THF, acetonitrile, and 1,2-difluorobenzene. S26
Figure S13. EPR-integrated intensity as a function of the temperature for [K(crypt-222)][(Cp*$_2$Y)$_2$(μ-flv•)] (**4**) in THF and [(Cp*$_2$Y)$_2$(μ-flv•)][Al(OC{CF$_3$}$_3$)$_4$] (**2**) in 1,2-difluorobenzene. S27

1.5 IR Spectroscopy S28

Figure S14. IR spectra of [K(crypt-222)](flv•) (**1**) and [(Cp*$_2$Y)$_2$(μ-flv•)][Al(OC{CF$_3$}$_3$)$_4$], (**2**), recorded on polycrystalline solids, superimposed with calculated spectra for the flv$^{1-•}$ anion in **1** and **2'**. S28
Figure S15. IR spectra of [(Cp*$_2$Y)$_2$(μ-flv)] (**3**) and [K(crypt-222)][(Cp*$_2$Y)$_2$(μ-flv•)] (**4**) recorded on polycrystalline solids, superimposed with calculated spectrum for **4'.** S29
Figure S16. Visualization of core vibrations of flv$^{1-•}$ (**1**$^-$). S29
Figure S17. Visualization of core vibrations of [(Cp$_2$Y)$_2$(μ-flv•)]$^+$ (**2'**). S30
Figure S18. Visualization of core vibrations of [(Cp$_2$Y)$_2$(μ-flv•)]$^-$ (**4'**). S30

1.6 UV-Vis Spectroscopy S31

Figure S19. Experimental UV-Vis spectrum of [K(crypt-222)](flv•), (**1**). S31
Figure S20. Experimental UV-Vis spectrum of [(Cp*$_2$Y)$_2$(μ-flv•)][Al(OC{CF$_3$}$_3$)$_4$], (**2**). S32
Figure S21. Experimental UV-Vis spectrum of [(Cp*$_2$Y)$_2$(μ-flv)] (**3**). S33
Figure S22. Experimental UV-Vis spectrum of [K(crypt-222)][(Cp*$_2$Y)$_2$(μ-flv•)] (**4**). S34
Figure S23. Superimposed experimental UV-Vis spectra of [K(crypt-222)](flv•) (**1**), [(Cp*$_2$Y)$_2$(μ-flv•)][Al(OC{CF$_3$}$_3$)$_4$], **2** [(Cp*$_2$Y)$_2$(μ-flv)] (**3**), [K(crypt-222)][(Cp*$_2$Y)$_2$(μ-flv•)] (**4**). S35

1.7 Cyclic Voltammetry S36

Figure S24. Full cyclic voltammogram and magnification of the reversible processes of flv^0, measured in THF. S36
Figure S25. Full cyclic voltammogram of [K(crypt-222)](flv•) (**1**), measured in THF. S37
Figure S26. Full cyclic voltammogram of [K(crypt-222)](flv•) (**1**), measured in 1,2-difluorobenzene. S37

Figure S27. Full cyclic voltammogram of [K(crypt-222)](flv•) (**1**), measured in acetonitrile. S38
Figure S28. Full cyclic voltammogram of [(Cp*$_2$Y)$_2$(μ-flv•)][Al(OC{CF$_3$}$_3$)$_4$] (**2**), measured in 1,2-difluorobenzene. S38
Figure S29. Full cyclic voltammogram of [(Cp*$_2$Y)$_2$(μ-flv)] (**3**), measured in 1,2-difluorobenzene. S39
Figure S30. Full cyclic voltammogram of [K(crypt-222)][(Cp*$_2$Y)$_2$(μ-flv•)] (**4**), measured in THF. S39
Table S4. Summary of the cyclic voltammetry measurements on compounds **1-4** and the free ligand flv^0. S40

1.8 Magnetic Data S40

Figure S31. Curie-Weiss plots (1/χ_M vs. *T*) of [(Cp*$_2$Y)$_2$(μ-flv•)][Al(OC{CF$_3$}$_3$)$_4$] (**3**) at 0.1 T and 1.0 T applied dc fields. S40
Table S5. Parameters used to fit Curie-Weiss plots of [(Cp*$_2$Y)$_2$(μ-flv•)][Al(OC{CF$_3$}$_3$)$_4$] (**3**). S40
Figure S32. Variable-temperature magnetic susceptibility of [(Cp*$_2$Y)$_2$(μ-flv•)][Al(OC{CF$_3$}$_3$)$_4$] (**3**) with fits. S41
Figure S33. Variable-temperature variable-field magnetization data of [(Cp*$_2$Y)$_2$(μ-flv•)][Al(OC{CF$_3$}$_3$)$_4$] (**2**), recorded at 2 K, 4 K, 6 K, 8 K, and 10 K with fits. S41
Table S6. Parameters used to fit variable-temperature variable-field magnetization data of [(Cp*$_2$Y)$_2$(μ-flv•)][Al(OC{CF$_3$}$_3$)$_4$] (**2**) to a Brillouin function for a *S* = ½ spin system. S41
Figure S34. Curie-Weiss plots (1/χ_M vs. *T*) of [K(crypt-222)][(Cp*$_2$Y)$_2$(μ-flv•)] (**4**) at 0.1 T and 1.0 T applied dc fields. S42
Table S7. Parameters used to fit Curie-Weiss plots of [K(crypt-222)][(Cp*$_2$Y)$_2$(μ-flv•)] (**4**). S42
Figure S35. Variable-temperature magnetic susceptibility of [K(crypt-222)][(Cp*$_2$Y)$_2$(μ-flv•)] (**4**) at 0.1 T and 1.0 T applied dc fields. S42
Figure S36. Variable-temperature variable-field-magnetization data [K(crypt-222)][(Cp*$_2$Y)$_2$(μ-flv•)] (**4**), recorded at 2 K, 4 K, 6 K, 8 K, and 10 K with fits. S43
Table S8. Parameters used to fit variable-temperature, variable-field magnetization data of [K(crypt-222)][(Cp*$_2$Y)$_2$(μ-flv•)] (**4**) to a Brillouin function for a *S* = ½ spin system. S43

1.9 DFT Calculations S44

1.9.1 Structure Optimizations S44

Figure S37. Optimized structure of the flv$^{1-•}$ anion in [K(crypt-222)](flv•) (**1**). S44
Table S9. Structural parameters of the optimized geometries of the flv$^{1-•}$ anion in [K(crypt-222)](flv•) (**1**) in comparison to the unoptimized values of **1** from single-crystal XRD. S44
Figure S38. Optimized structure of [(Cp$_2$Y)$_2$(μ-flv•)]$^+$ (**2'**). S45
Table S10. Structural parameters of the optimized geometries of **2'** in comparison to the unoptimized values of [(Cp*$_2$Y)$_2$(μ-flv•)][Al(OC{CF$_3$}$_3$)$_4$], (**2**) from single-crystal XRD. S45
Table S11. Selected angles of the optimized geometries of **2'** in comparison to the unoptimized values of [(Cp*$_2$Y)$_2$(μ-flv•)][Al(OC{CF$_3$}$_3$)$_4$], (**2**) from single-crystal XRD. S46

Figure S39. Optimized structure of $[(Cp_2Y)_2(\mu\text{-flv}\bullet)]^-$ (**4'**). S46
Table S12. Structural parameters of the optimized geometries of **4'** in comparison to the unoptimized values of $[K(\text{crypt-222})][(Cp^*{}_2Y)_2(\mu\text{-flv}\bullet)]$ (**4**), from single-crystal XRD. S47
Table S13. Selected angles of the optimized geometries of $[(Cp_2Y)_2(\mu\text{-flv}\bullet)]^-$ (**4'**) in comparison to the unoptimized values of $[K(\text{crypt-222})][(Cp^*{}_2Y)_2(\mu\text{-flv}\bullet)]$ (**4**) from single-crystal XRD. S48
Figure S40. Optimized structure of $[(Cp_2Y)_2(\mu\text{-Bbim}\bullet)]^-$. S48
Table S14. Structural parameters of the optimized geometries of $[(Cp_2Y)_2(\mu\text{-Bbim}\bullet)]^-$ in comparison to the unoptimized values of $[K(\text{crypt-222})][(Cp^*{}_2Y)_2(\mu\text{-Bbim}\bullet)]$ from single-crystal XRD. S48
Table S15. Selected angles of the optimized geometries of $[(Cp_2Y)_2(\mu\text{-Bbim}\bullet)]$ in comparison to the unoptimized values of $[K(\text{crypt-222})][(Cp^*{}_2Y)_2(\mu\text{-Bbim}\bullet)]$ from single-crystal XRD. S49
Figure S41. Frontier orbital energies for all calculated compounds in eV. S50

1.9.2 Natural Localized Molecular Orbital Analysis S50

Table S16. Results of the hybridization/polarization analysis of $[(Cp_2Y)_2(\mu\text{-flv}\bullet)]^+$ (**2'**) NLMOs for the *α*-spin manifold. S50
Table S17. Results of the hybridization/polarization analysis of $[(Cp_2Y)_2(\mu\text{-flv}\bullet)]^+$ (**2'**) NLMOs for the *β*-spin manifold. S51
Table S18. Results of the second order perturbation analysis for $[(Cp_2Y)_2(\mu\text{-flv}\bullet)]^+$ (**2'**). S51
Table S19. Results of the hybridization/polarization analysis of $[(Cp_2Y)_2(\mu\text{-flv}\bullet)]^-$ (**4'**) NLMOs for the *α*-spin manifold. S52
Table S20. Results of the hybridization/polarization analysis of $[(Cp_2Y)_2(\mu\text{-flv}\bullet)]^-$ (**4'**) NLMOs for the *β*-spin manifold. S53
Table S21. Results of the second order perturbation analysis for $[(Cp_2Y)_2(\mu\text{-flv}\bullet)]^-$ (**4'**). S53
Table S22. Results of the hybridization/polarization analysis for NLMOs of $[(Cp_2Y)_2(\mu\text{-Bbim}\bullet)]^-$ for the *α*-spin manifold. S54
Table S23. Results of the hybridization/polarization analysis for NLMOs of $[(Cp_2Y)_2(\mu\text{-Bbim}\bullet)]^-$ for the *β*-spin manifold. S54
Table S24. Results of the second order perturbation analysis for $[(Cp_2Y)_2(\mu\text{-Bbim}\bullet)]^-$. S54

1.9.3 EPR Parameters S55

Table S25. Calculated EPR parameters and electron densities for $\text{flv}^{1-\bullet}$ anion in $[K(\text{crypt-222})](\text{flv}\bullet)$ (**1**). S55
Table S26. Calculated EPR parameters and electron densities for $[(Cp_2Y)_2(\mu\text{-flv}\bullet)]^+$ (**2'**), a contracted model of $[(Cp^*{}_2Y)_2(\mu\text{-flv}\bullet)][Al(OC\{CF_3\}_3)_4]$, (**2**). S56
Table S27. Calculated EPR parameters and electron densities for $[(Cp_2Y)_2(\mu\text{-flv}\bullet)]^-$ (**4'**), a contracted model of $[K(\text{crypt-222})][(Cp^*{}_2Y)_2(\mu\text{-flv}\bullet)]$ (**4**). S57
Table S28. Calculated EPR parameters and electron densities for $[(Cp_2Y)_2(\mu\text{-Bbim}\bullet)]$, a contracted model of $[K(\text{crypt-222})][(Cp^*{}_2Y)_2(\mu\text{-Bbim}\bullet)]$. S58

1.9.4 TDDFT S59

Table S29. TDDFT-calculated transitions for $\text{flv}^{1-\bullet}$ ($\mathbf{1}^-$). S59
Table S30. TDDFT-calculated transitions for $[(Cp_2Y)_2(\mu\text{-flv}\bullet)]^+$ (**2'**). S60

Table S31. TDDFT-calculated transitions for $[(Cp_2Y)_2(\mu\text{-flv}\bullet)]^-$, (**4'**). S62

1.9.5 Quantum Theory of Atoms in Molecules (QTAIM) Analysis S64

Figure S42. Electron localization function (ELF, left) and Laplacian of the electron density (right) maps of $flv^{1-\bullet}$ ($\mathbf{1}^-$). S64
Table S32. Real space values of critical points as obtained from QTAIM analysis on $flv^{1-\bullet}$ ($\mathbf{1}^-$). S64
Figure S43. Electron localization function (ELF, left) and Laplacian of the electron density (right) maps of the $[(Cp_2Y)_2(\mu\text{-flv}\bullet)]^+$ cation (**2'**). S65
Table S33. Real space values of critical points as obtained from QTAIM analysis on the $[(Cp_2Y)_2(\mu\text{-flv}\bullet)]^+$ cation (**2'**). S65
Figure S44. Electron localization function (ELF, left) and Laplacian of the electron density (right) maps of the $[(Cp_2Y)_2(\mu\text{-flv}\bullet)]^-$ anion (**4'**). S66
Table S34. Real space values of critical points as obtained from QTAIM analysis on the $[(Cp_2Y)_2(\mu\text{-flv}\bullet)]^-$ anion (**4'**). S67
Figure S45. Electron localization function (ELF, left) and Laplacian of the electron density (right) maps of the flv^0. S68
Table S35. Real space values of critical points as obtained from QTAIM analysis on flv^0. S68
Figure S46. Electron localization function (ELF, left) and Laplacian of the electron density (right) maps of the $[(Cp_2Y)_2(\mu\text{-Bbim}\bullet)]^-$ anion. S69
Table S36. Real space values of critical points as obtained from QTAIM analysis on the $[(Cp_2Y)_2(\mu\text{-Bbim}\bullet)]^-$ anion. S69
Figure S47. Graphical representation of aromaticity descriptors obtained via QTAIM analysis at BCP of flv^0 and model compounds $\mathbf{1}^-$, **2'** and **4'**. S71
Figure S48. Plots of the Laplacian of the spin density $\nabla^2 s(r)$ for $flv^{1-\bullet}$ ($\mathbf{1}^-$), $[(Cp_2Y)_2(\mu\text{-flv}\bullet)]^+$ (**2'**), $[(Cp_2Y)_2(\mu\text{-flv}\bullet)]^-$ (**4'**) and $[(Cp_2Y)_2(\mu\text{-Bbim}\bullet)]^-$. S72
Table S37. Real space values of critical points as obtained from spin density topology analysis on the $flv^{1-\bullet}$ anion ($\mathbf{1}^-$). S73
Table S38. Real space values of critical points as obtained from spin density topology analysis on the $[(Cp_2Y)_2(\mu\text{-flv}\bullet)]^+$ cation (**2'**). S74
Table S39. Real space values of critical points as obtained from spin density topology analysis on the $[(Cp_2Y)_2(\mu\text{-flv}\bullet)]^-$ anion (**4'**). S75

1.9.6 Coordinates of Optimized Structures S76

1.10 References **S87**

1.1 Experimental Methods

1.1.1 General Information

All manipulations were performed under inert conditions using either standard Schlenk techniques or an argon-filled glovebox. House nitrogen was purified through a MBraun HP-500-MO-OX gas purifier. ^{n}Hexane was purified by refluxing over CaH_2, toluene and THF were purified by refluxing over potassium and subsequent distillation. THF was subsequently stirred over NaK for at least a day and distilled a second time prior to use. Acetonitrile was refluxed for 5 days over $CaCl_2$ before distillation. A drop of sodium benzophenone radical solution was used as an indicator to prove the absence of water and oxygen within the glovebox. The NMR solvent THF-d_8 was dried by storing over Na/K alloy for several days in a glovebox and filtered prior to use.

The chemicals pentamethylcyclopentadiene (Cp*H), allylmagnesium chloride (2.0 M in THF), and anhydrous YCl_3 were purchased from Sigma Aldrich and used as received. Potassium bistrimethylsilylamide ($KN(Si(CH_3)_3)_2$) and 2.2.2-cryptand (crypt-222) were purchased from Sigma Aldrich, the former was recrystallized from toluene and the latter from ^{n}hexane prior to use. KCp*,[1] ($HNEt_3$)(BPh_4),[2] Cp*$_2$Y(BPh_4),[3] H_2flv,[4] flv^0,[4] (Thian•)[Al(OC{CF_3}$_3$)$_4$][5] and KC_8,[6] were synthesized according to literature procedures.

1.1.2 Synthesis of [K(crypt-222)](flv•), (1)

Flv^0 (10.2 mg, 0.0439 mmol) and crypt-222 (16.4 mg, 0.0436 mmol) were combined as solids and then dispersed in THF (4.6 mL) in a 7 mL vial. To this mixture, solid KC_8 (5.9 mg, 0.0436 mmol) was added in one portion, affording an immediate color change from red to dark blue. The mixture was gently shaken for approximately 10 min, at which point the potassium of all KC_8 fully reacted. Subsequently, the mixture was filtered into a 7 mL vial. The insoluble solids were washed once with a small amount of THF (0.4 mL) and the clear, dark blue filtrate was moved to the freezer for crystallization at −35 °C. Dark blue crystals suitable for single-crystal X-ray diffraction analysis were grown from a concentrated THF solution over the course of two days. These crystals were washed once with cold THF (~0.5 mL) and dried under vacuum for 50 min (17.0 mg, 0.0262 mmol, 60%). Anal. Calcd for $C_{32}H_{44}KN_6O_6$: C, 59.33; H, 6.85; N, 12.97; found: C, 59.51 H, 6.71; N, 12.90. IR (ATR, cm^{-1}): 3043 (vw), 2963 (vw), 2879 (w), 2814 (w), 1526 (s), 1479 (m), 1450 (s), 1356 (m), 1341 (vs), 1301 (s), 1258 (s), 1223 (s), 1131 (s), 1096 (vs), 1055 (s), 1032 (m), 1012 (s), 1079 (vs), 945 (s), 932 (s); 831 (m), 820 (m), 745 (vs).

1.1.3 Synthesis of [(Cp*$_2$Y)$_2$(μ-flv•)][Al(OC{CF_3}$_3$)$_4$] (2)

Compound **3** (12.1 mg, 0.0127 mmol) and (Thian•)[Al(OC{CF_3}$_3$)$_4$] (15.1 mg, 0.0128 mmol) were dissolved separately in DCM (3 mL and 1 mL, respectively) and then the oxidant was added to **3** in one portion in a 20 mL vial. An immediate color change from bright yellow to dark green was observed, and the reaction mixture was stirred for 12 min. Subsequently, the reaction mixture was filtered, and the filtrate evaporated to dryness. The obtained dark yellow/greenish solid was washed three times with toluene (~1.5 mL in total) and the insoluble solid was dried under vacuum. This dark green solid (17.8 mg, 73% crude yield) was redissolved in DCM (1.5 mL), filtered, and moved to the freezer for crystallization. Dark green crystals suitable for single-crystal X-ray diffraction analysis were obtained from a concentrated DCM solution at −35 °C over the course of four days (14.9 mg, 0.0078 mmol, 61%). Anal. Calcd for $C_{70}H_{68}AlF_{36}N_4O_4Y_2 \cdot CH_2Cl_2$: C, 42.58; H, 3.52; N, 2.80; found: C, 42.90; H, 3.34; N, 2.88. IR (ATR, cm^{-1}): 2913 (vw), 2863 (vw),

1600 (vw), 1538 (w), 1491 (vw), 1450 (vw), 1435 (vw), 1381 (vw), 1351 (m), 1295 (s), 1273 (vs), 1210 (vs), 1161 (s), 1144 (s), 1133 (s), 1019 (vw), 969 (vs), 908 (m), 833 (m), 753 (s), 725 (vs).

1.1.4 Synthesis of K_2flv

A THF (2 mL) solution of $KN(Si(CH_3)_3)_2$ (313.7 mg, 1.5726 mmol) was added to a suspension of H_2flv (171.1 mg, 0.7304 mmol) in THF (12 mL) in a 20 mL vial, resulting in an immediate color change from golden to green fluorescent. The mixture was stirred for 2.5 h at room temperature, and then left to settle for 2 h. The clear, intense green supernatant was removed by decanting, and the insoluble yellow solid was washed with fresh THF (5 mL). After removing the supernatant again by decanting, the insoluble solid was dried under vacuum for 1 h at 60 °C and used without further purification (175.3 mg, 0.5647 mmol, 77 %). Anal. Calcd for $C_{14}H_8K_2N_4$: C, 54.17; H, 2.60; N, 18.05; found: C, 53.86; H, 2.59; N, 17.65.

1.1.5 Synthesis of [(Cp*$_2$Y)$_2$(μ-flv)] (3) via Reduction of flv^0

KC_8 (5.1 mg, 0.0377 mmol) was added to a suspension of flv^0 (8.6 mg, 0.0370 mmol) in THF (2 mL) in a 20 mL vial, resulting in a color change from red to dark blue over the course of a few seconds. This was mixed via pipette for approximately 5 min, and subsequently added to a stirring solution of Cp*$_2$Y(BPh_4) (50.3 mg, 0.074 mmol) in THF (5 mL) in a 20 mL vial, affording an immediate color change from dark blue to dark green. The mixture was stirred for another 15 min whereby the color became gradually more orange and was then filtered to remove gray and colorless solids, presumably $KBPh_4$ and graphite. The clear, bright orange filtrate was evaporated to dryness to afford a yellow-orange solid (53.8 mg). This solid was extracted three times with toluene (8 mL in total) and filtered through a Celite plug. The clear, orange filtrate was evaporated to dryness to give an orange solid (29.2 mg, 83% crude yield) which was dissolved in 1.5 mL THF, filtered, and stored at −35 °C. Orange crystalline materials of **3** suitable for single-crystal X-ray diffraction analysis were obtained over the course of three days. These crystals were dried under vacuum for 1 h, after removal of the orange mother liquor, to yield an orange powder (8.5 mg, 0.0089 mmol, 24 %).

1.1.6 Synthesis of [(Cp*$_2$Y)$_2$(μ-flv)] (3) via Salt Metathesis using K_2flv

Solid K_2flv (38.6 mg, 0.1243 mmol) was added to a stirring solution of Cp*$_2$Y(BPh_4) (168.2 mg, 0.2479 mmol) in THF (10 mL) in a 20 mL vial, resulting in an immediate color change from colorless to bright yellow accompanied by the formation of a colorless solid, presumably $KBPh_4$. After stirring at room temperature for 3 h, the solid was filtered through a Celite plug to give a clear, bright yellow filtrate, which was evaporated to dryness. The resulting yellow-orange flaky solid was then dissolved in hot THF (7 mL) and filtered hot which removed a colorless insoluble solid. The hot, orange filtrate was cooled down to first room temperature and then to −35 °C in the freezer for crystallization of **3**. Orange crystals of **3** were grown over the course of two days, suitable for single-crystal X-ray diffraction analysis. The crystal with the structural parameters listed in Table 1.2 was grown through this route. These crystals were washed once with THF (0.5 mL) and then dried under vacuum for 1.5 h. **3** was obtained as large orange crystals (77.9 mg, 0.0819 mmol, 66%). Anal. Calcd for $C_{54}H_{68}N_4Y_2$: C, 68.20; H, 7.21; N, 5.89; found: C, 68.10; H, 7.37; N, 5.81. ^{1}H NMR (THF-d_8, 500 MHz) δ (ppm) = 6.61 (sex, 4H, flv-H_{inner}), 6.14 (sex, 4H, flv-$H_{terminal}$), 1.99 (s, 60H, Cp*-H). ^{13}C NMR (THF-d_8, 125 MHz) δ (ppm) = 138.6, 123.1, 122.1, 118.6, 11.4 (s, Cp*-Me). IR (ATR, cm^{-1}): 3026 (vw), 2893 (m), 2855 (m), 2718 (vw), 1600 (vw), 1563

(vw), 1473 (s), 1457 (vs), 1337 (s), 1301 (s), 1279 (s), 1238 (s), 1149 (w), 1117 (w), 1060 (vw), 1032 (m), 1021 (m), 966 (m), 915 (w), 844 (vw), 800 (vw), 779 (vw), 743 (vs).

1.1.7 Synthesis of [K(crypt-222)][($Cp^*_2Y)_2$(μ-flv•)] (4)

Complex **3** (42.9 mg, 0.0451 mmol) and crypt-222 (1.9 mg, 0.050 mmol) were dissolved in THF (11 mL) in a 20 mL vial, and a suspension of KC_8 (6.10 mg, 0.0451 mmol) in THF (1 mL) was added at once to the bright yellow solution. An immediate color change to intense dark green was observed, which gradually shifted towards dark blue over the course of 5 min. After 30 min of stirring, the reaction mixture was filtered to remove insoluble graphite, which was washed twice with THF (1 mL in total). The clear, dark blue filtrate was stored in the freezer at −35 °C for crystallization. The first crop of crystals suitable for single-crystal X-ray diffraction analysis were isolated after 2 days. These were washed once with THF (0.5 mL) and dried under vacuum for 1 h to afford **4** as a dark blue crystalline solid (5.4 mg, 0.0040 mmol, 9%). The mother liquor was evaporated to dryness, and the resulting dark blue-green solid was washed four times with toluene to remove the unreacted, bright yellow complex **3**. After drying the remaining toluene-insoluble solid in vacuum, the crude product (46.4 mg, 0.0340 mmol) was dissolved in THF (5 mL), filtered, and stored in the freezer for further crystallization. After four days, a second crop of dark blue crystals was isolated, which were washed twice with THF (1 mL in total) and dried under vacuum for 1 h to afford crystalline **4** (34.1 mg, 0.0250 mmol, 55%). Anal. Calcd for $C_{72}H_{104}KN_6O_6Y_2$: C, 63.28; H, 7.67.; N, 6.15; found: C, 63.28; H, 7.91; N, 6.09. IR (ATR, cm^{-1}): 3037 (vw), 2951 (vw), 2881 (m), 2851 (m), 2722 (vw), 2607 (vw), 1540 (vw), 1456 (s), 1338 (vs), 1298 (s), 1274 (s), 1217 (s), 1175 (s), 1101 (vs), 1077 (vs), 1030 (s), 948 (s), 930 (s), 885 (w), 829 (w), 820 (w), 753 (w), 718 (s).

1.1.8 Single Crystal X-ray Diffraction

Data on **1**, **2** and **3** were collected on a XtaLAB Synergy DualflexHyPix four-circle diffractometer, equipped with a HyPix Hybrid Pixel Array Detector. The crystals were kept at 100 K during data collection. Data were measured using CuK_α radiation. The maximum resolution that was achieved was Θ = 71.296° (0.81 Å). The CrysAlisPro software package[7] was used to retrieve and refine the cell parameters, and was used for data reduction. Correction for absorption effects was performed using a numerical correction based on gaussian integration over a multifaceted crystal model and an empirical correction using spherical harmonics, implemented in SCALE3 ABSPACK[8] scaling algorithm (spherical harmonics and frame scaling).

Data of complex **4** were collected on a Bruker CCD (charge coupled device) based diffractometer using MoKα radiation. The instrument was equipped with an Oxford Cryostream low-temperature apparatus operating at 173 K. Data were measured using omega and phi scans of 1.0° per frame for 30 s. The total number of images was based on results from the program COSMO[9] where redundancy was expected to be 4.0 and completeness of 100% out to 0.83 Å. Cell parameters were retrieved using APEX II software[10] and refined using SAINT on all observed reflections. Data reduction was performed using the SAINT software[11] which corrects for Lp. Scaling and absorption corrections were applied using SADABS[12] multi-scan technique.

The structures were solved with the ShelXT[13] structure solution program using intrinsic phasing and refined with version 2018/3 of ShelXL[14] using least squares minimization as implemented in Olex2[15]. All non-hydrogen atoms are refined anisotropically. Hydrogen atoms were calculated by geometrical methods and refined as a riding model. The crystals used for the diffraction study

showed no decomposition during data collection. Crystal data and structure refinement for all compounds are shown in Table S1.

1.1.9 NMR Spectroscopy

^1H and ^{13}C NMR spectra of **1** were recorded on a 500 MHz Agilent DirectDrive2 and referenced to the residual solvent signals (THF-d_8: δ_H = 3.58 ppm, δ_C = 67.6 ppm). Signal multiplicities are abbreviated as: *s* (singlet), *d* (doublet), *m* (multiplet), *br* (broad). Air-sensitive samples were prepared in a nitrogen-filled glovebox using standard NMR tubes air-tight sealed.

1.1.10 EPR Spectroscopy

Variable-temperature solution-state X-Band EPR spectra of **1** and **2** were recorded on a Bruker EMX-plus EPR in THF (0.88 mmol/L) and difluorobenzene (0.67 mmol/L) solutions, respectively. For **4**, these measurements were carried out in THF solution (0.22 mmol/L) on a Bruker Elexsys E-680X. The spectra were simulated by using the EasySpin software package[16] for MATLAB.

1.1.11 IR Spectroscopy

IR spectra were taken with an Agilent Cary 630 ATR spectrometer residing in an argon-filled glovebox.

1.1.12 UV-Vis Spectroscopy

In an argon-filled glovebox, UV-Vis spectra were collected in 1 cm cuvettes with an Agilent Cary 60 spectrometer, equipped with QP600-1-SR fiber optics and a Square One cuvette holder from Ocean Insight.

1.1.13 Cyclic Voltammetry

Cyclic voltammetry experiments were conducted under an inert atmosphere in an argon-filled glovebox using a PGSTAT204 potentiostat from Metrohm. A three-electrode setup involving glassy carbon working electrodes, platinum spring counter electrodes and varying reference electrodes were used. All measurements were conducted cycling the solvent range four-fold at a 100 mV/s scan rate and $E_{1/2}$ averaged, while all voltammograms displayed in the main text constitute the second scan. Measurements on **1** were internally referenced against ferrocene. Due to chemical incompatibility of the organometallic complexes with ferrocene, the measurements of compounds **2**-**4** were externally referenced to ferrocene solutions with identical supporting electrolyte concentrations and electrode setup.

Compounds **1**, **2**, and **3** were measured as 1,2-difluorobenzene solutions. Flv^0 and **4** were measured as THF solutions. For all measurements, 220 mmol/L electrolyte concentrations (($^nBu_4N)PF_6$) were used except for **4** which was measured with a 250 mmol/L concentration. As pseudo reference electrode, an Ag wire was utilized for all compounds except for **4** which was measured against a Pt wire. Sample concentrations of 3 mmol/L were used for flv^0 and compounds **1**-**3**, while a 1 mmol/L solution was used for **4** due to poor solubility of the analyte in THF.

The effects of the solvent on the measured $E_{1/2}$ were investigated by conducting control experiments on **1** in THF and acetonitrile as well. The comparison of $E_{1/2}$ for **1** in MeCN, DFB and

THF shows a gradual shift towards more negative potentials with decreasing solvent polarity (Table S4).

1.1.14 Elemental Analysis

Elemental analysis was carried out with a PerkinElmer 2400 Series II CHNS/O analyzer with assistance by Francis Delano IV and Saroshan Deshapriya (for **1** and **2**), and by Dr. Rui Huang (for **3** and **4**). The crystalline compounds of all samples (~1 – 3 mg) were weighed into tin sample holders and folded multiple times to ensure proper sealing from surrounding atmosphere. The samples were then transferred to the instrument under exclusion of air in a capped and taped scintillation vial.

1.1.15 Magnetic Susceptibility Measurements

Magnetic susceptibility data were collected on a Quantum Design MPMS3 Superconducting Quantum Interference Device (SQUID) magnetometer. The magnetic samples of $[(Cp^*_2Y)_2(\mu\text{-flv}^\bullet)][Al(OC\{CF_3\}_3)_4]$, **2**, and $[K(\text{crypt-222})][(Cp^*_2Y)_2(\mu\text{-flv}^\bullet)]$, **4,** were prepared by saturating and covering dried, crushed crystalline solids (**2**: 28.6 mg, 1.491×10^{-5} mol; **4**: 26.6 mg, 1.946×10^{-5} mol) with molten eicosane (**2**: 60.8 mg; **4**: 53.4 mg) at 60 °C to prevent crystallite torquing and to provide good thermal contact between the sample and the bath. The samples were sealed airtight and transferred to the magnetometer. The raw static field susceptibility data were corrected for diamagnetic contributions via subtraction from a blank sample, prepared using the amounts of eicosane in an identical sample design as the samples of **2** and **4** and measured in the same way as the samples. The core diamagnetism was estimated using Pascal's constants.[17]

1.1.16 Computational Methods

DFT calculations were carried out on a truncated model of the paramagnetic flv compounds **1**, **2**, and **4** using the Orca program suite (version 5.0.3).[18,19] The starting coordinates for the geometry optimization were taken from the crystallographically obtained molecular structures, and for **2** and **4** all methyl groups of the Cp* rings were substituted by hydrogen atoms in order to reduce computational costs (from here on referred to as **2'** and **4'**, respectively). The computational model of **1**, $\mathbf{1}^-$, was not further contracted. The counterions for $\mathbf{1}^-$, **2'** and **4'** were omitted in all calculations. Geometry optimizations were carried out using the unrestricted uTPSSh functional[19,20] with D3BJ dispersion correction.[20,21] The RIJCOSX algorithm was used to speed up the computations of Coulomb and Hartree-Fock exchange integrals,[22,23] accompanied by the RI-J auxiliary basis set def2/J.[24] Normal SCF convergence criteria and the slowconv keywords were employed to aid the convergence process. The def2 integration grid was chosen throughout. The geometry was first optimized using the def2-SVP level of theory and subsequently reoptimized as def2-SVP for the Cp atoms and def2-TZVP on the Y and flv atoms.[24] Stuttgart-Cologne electron core potentials were used for the Y atoms as implemented via the def2-SD keyword.[25] The minimum structures of $\mathbf{1}^-$, **2'**, and **4'** were confirmed via frequency calculations, revealing the absence of any imaginary frequencies for **1** and **2'**, and two imaginary frequencies for **4'** <10 cm^{-1}, which are associated to in-plane rotations of the Cp ligands that could not be removed through further optimization cycles.

Bond analysis by means of NLMO and QTAIM analysis were carried out on this geometry using the NBO7.0 and MultiWfn programs on the SVP(Cp)/TZVP(Y,flv) level.[26,27] All plots of real-space functions within a plane were prepared via MultiWfn.

Calculated IR frequencies were converted into spectra via application of a 8 cm^{-1} Lorentzian broadening, applied via MultiWfn, and empirically shifted for **1**$^{-}$, **2'**, and **4'** by −38 cm^{-1}.

For TDDFT and EPR calculations, this geometry was optimized further employing the CPCM solvent model for THF on the SVP(Cp)/TZVP(Y,flv) level of theory, which was also used for the following property calculations. EPR calculations were performed using the uPBE0 functional[28–30] with relativistic segmented all-electron relativistically contracted (SARC)[31] and second order Douglas-Kroll (DKH2)[25,32] approaches using SARC-ZORA-TZVP (Y)[33] and ZORA-def2-TZVP (flv)[28] and ZORA-def2-SVP (Cp)[28] level of theory, and their respective DKH2 counterparts.[28] TDDFT calculations were carried out under consideration of 150 excited states using the same level of theory as utilized for the EPR calculations with the SARC approach.

For comparison, NLMO and EPR calculations were also carried out on the contracted model of the previously reported [K(crypt-222)][$(Cp^*_2Y)_2$(μ-Bbim$^•$)], $[(Cp_2Y)_2$(μ-Bbim$^•$)$]^-$, using the same approximations and level of theory.[34]

All orbital visualizations were generated via VMD.[35]

1.2 Single Crystal X-ray Diffraction

Table S1. Crystallographic data and structural refinement of [K(crypt-222)](flv•) (**1**), [(Cp*$_2$Y)$_2$(μ-flv•)][Al(OC{CF$_3$}$_3$)$_4$] (**2**), [(Cp*$_2$Y)$_2$(μ-flv)] (**3**), and [K(crypt-222)][(Cp*$_2$Y)$_2$(μ-flv•)] (**4**). **2** crystallized with one CH_2Cl_2 molecule in the lattice. **3** crystallized with two THF molecules in the lattice. **4** crystallized with four THF molecules in the lattice.

Compound	**1**	**2**·CH_2Cl_2	**3**·2THF	**4**·4THF
CCDC no.	2347986	2347988	2348009	2347984
Empirical formula	$C_{32}H_{44}KN_6O_6$	$C_{71}H_{70}AlCl_2F_{36}N_4O_4Y_2$	$C_{62}H_{84}N_4O_2Y_2$	$C_{88}H_{136}KN_6O_{10}Y_2$
Formula weight	647.83	2003.01	1095.15	1654.94
Temperature/K	99.99(11)	100.15	100.00(10)	173.15
Crystal system	triclinic	monoclinic	triclinic	triclinic
Space group	*P*−1	*I*2/a	*P*−1	*P*−1
a (Å)	11.7713(3)	16.95440(10)	9.82860(10)	12.2894(17)
b (Å)	12.7847(3)	17.86180(10)	10.00560(10)	13.5418(19)
c (Å)	13.4766(3)	26.2088(2)	14.1765(2)	26.672(4)
α (°)	64.380(2)	90	96.9570(10)	87.521(2)
β (°)	75.260(2)	96.0460(10)	97.9430(10)	89.612(2)
γ (°)	65.593(2)	90	93.0320(10)	78.667(2)
Volume (Å^3)	1658.20(8)	7892.82(9)	1367.15(3)	4348.1(10)
Z	2	4	1	2
ρ_{calc} (g/cm^3)	1.297	1.686	1.330	1.264
μ (mm^{-1})	1.829	3.931	3.184	1.435
F(000)	690.0	4020.0	578.0	1766.0
Crystal size (mm^3)	0.125 × 0.102 × 0.065	0.19 × 0.15 × 0.13	0.175 × 0.141 × 0.114	0.428 × 0.146 × 0.137
Radiation	CuKα (λ = 1.54184)	CuKα (λ = 1.54184)	Cu Kα (λ = 1.54184)	MoKα (λ = 0.71073)
2Θ range for data collection (°)	7.306 to 160.626	5.998 to 155.494	6.348 to 159.43	3.056 to 50.662
Index ranges	$-14 \le h \le 15$, $-16 \le k \le 16$, $-17 \le l \le 16$	$-20 \le h \le 21$, $-22 \le k \le 16$, $-33 \le l \le 32$	$-10 \le h \le 12$, $-12 \le k \le 12$, $-17 \le l \le 16$	$-14 \le h \le 14$, $-16 \le k \le 16$, $-32 \le l \le 32$
Reflections collected	32022	54092	17589	69299
Independent reflections	7025 [R_{int} = 0.0349, R_σ = 0.0249]	8338 [R_{int} = 0.0363, R_σ = 0.0233]	5710 [R_{int} = 0.0362, R_σ = 0.0341]	15811 [R_{int} = 0.0745, R_σ = 0.0662]
Data/restraints/ parameters	7025/0/406	8338/1501/819	5710/0/326	15811/0/966
Goodness-of-fit on F^2	1.060	1.057	1.082	1.021
Final *R* indexes (*I* > = 2 σ (*I*))	R_1 = 0.0446, wR_2 = 0.1155	R_1 = 0.0469, wR_2 = 0.1180	R_1 = 0.0300, wR_2 = 0.0800	R_1 = 0.0547, wR_2 = 0.1293
Final *R* indexes (all data)	R_1 = 0.0492, wR_2 = 0.1187	R_1 = 0.0496, wR_2 = 0.1203	R_1 = 0.0315, wR_2 = 0.0808	R_1 = 0.0871, wR_2 = 0.1443
Largest diff. peak/hole (e Å^{-3})	0.54/-0.56	1.17/-0.85	0.58/-0.91	0.80/-0.60

Table S2. Selected structural parameters of [K(crypt-222)](flv•) (**1**), [$(Cp^*{}_2Y)_2$(μ-flv•)][$Al(OC\{CF_3\}_3)_4$] (**2**), and [$(Cp^*{}_2Y)_2$(μ-flv)] (**3**).

1		2		3	
**Atoms**	**Distance (Å)**	**Atoms**	**Distance (Å)**	**Atoms**	**Distance (Å)**
			**Central C–C**		
C2–C2'	1.465(3)	C2–C2'	1.431(6)	C2–C2'	1.452(4)
C11–C11'	1.467(3)				
			**N–C$_{central}$**		
N4–C11	1.348(3)	N1–C2	1.350(6)	N1–C2	1.338(2)
N3–C11	1.342(2)	N2–C2	1.345(4)	N2–C2	1.343(3)
N1–C2	1.342(2)	N1'–C2'	1.350(3)	N1'–C2'	1.338(2)
N2–C2	1.347(2)	N2'–C2'	1.345(4)	N2'–C2'	1.343(3)
			**N–C$_{peripheral}$**		
N3–C10	1.357(3)	N1–C1	1.366(4)	N1–C3	1.393(2)
N4–C12	1.358(2)	N2–C3	1.372(3)	N2–C1	1.397(2)
N1–C1	1.361(2)	N1'–C1'	1.366(4)	N1'–C3'	1.393(2)
N2–C3	1.356(2)	N2'–C3'	1.372(3)	N2'–C1'	1.397(2)
			**Phenyl C–C**		
C10–C9	1.415(3)	C3–C4	1.411(4)	C3–C4	1.401(3)
C9–C8	1.372(3)	C4–C5	1.374(4)	C4–C5	1.385(3)
C14–C8	1.409(3)	C5–C6	1.406(4)	C5–C6	1.393(3)
C13–C14	1.371(3)	C6–C7	1.364(4)	C6–C7	1.387(3)
C12–C13	1.415(3)	C1–C7	1.419(4)	C1–C7	1.399(3)
C1–C7	1.418(3)	C1–C3	1.425(4)	C1–C3	1.415(3)
C7–C6	1.372(3)	C3'–C4'	1.411(4)	C1'–C7'	1.399(3)
C5–C6	1.407(3)	C4'–C5'	1.374(4)	C7'–C6'	1.387(3)
C4–C5	1.370(3)	C5'–C6'	1.406(4)	C5'–C6'	1.393(3)
C3–C4	1.418(2)	C6'–C7'	1.364(4)	C4'–C5'	1.385(3)
		C1'–C7'	1.419(4)	C3'–C4'	1.401(3)
		C1'–C3'	1.425(4)	C1'–C3'	1.415(3)
			**Y–N**		
		Y1–N1	2.439(2)	Y1–N1	2.392(2)
		Y1–N2	2.434(2)	Y1–N2	2.382(2)
		Y1'–N1'	2.439(2)	Y1'–N1'	2.392(2)
		Y1'–N2'	2.434(2)	Y1'–N2'	2.382(2)
**Atoms**	**Angles (°)**	**Atoms**	**Angles (°)**	**Atoms**	**Angles (°)**
(Ph–Ph)1	0.1(1)	Ph–Ph	0.1(1)	Ph–Ph	0.1(1)
(Ph–Ph)1	0.1(1)				
		Y1–N1–N2'–Y1'	−4.9(4)	Y1–N1–N2'–Y1'	−16.3(3)
		Y1–N2–N1'–Y1'	−4.9(4)	Y1–N2–N1'–Y1'	−16.3(3)
		Cnt–Y1-Cnt	141.6	Cnt–Y1-Cnt	141.0
		Cnt–Y1'-Cnt	141.6	Cnt–Y1'-Cnt	141.0

Table S3. Selected structural parameters of [K(crypt-222)][($Cp^*_2Y)_2$(μ-flv•)] (**4**).

**4**			
**Atoms**	**Distance (Å)**	**Atoms**	**Distance (Å)**
**Central C–C**		**Phenyl C–C continued**	
C1–C1'	1.373(7)	C9–C10	1.395(5)
C8–C8'	1.373(7)	C10–C11	1.391(5)
**N–C$_{central}$**		C11–C12	1.379(6)
N1–C1	1.384(5)	C12–C13	1.388(5)
N2–C1	1.387(4)	C13–C14	1.396(5)
N1'–C1'	1.384(5)	C14–C9	1.434(5)
N2'–C1'	1.387(4)	C9'–C10'	1.395(5)
N3–C8	1.380(5)	C10'–C11'	1.391(5)
N4–C8	1.391(5)	C11'–C12'	1.379(6)
N3'–C8'	1.380(5)	C12'–C13'	1.388(5)
N4'–C8'	1.391(5)	C13'–C14'	1.396(5)
**N–C$_{peripheral}$**		C14'–C9'	1.434(5)
N1–C2	1.389(5)	**Y–N**	
N2–C6	1.384(5)	Y1–N1	2.300(3)
N1'–C2'	1.389(5)	Y1–N2	2.320(3)
N2'–C6'	1.384(5)	Y1'–N1'	2.300(3)
N3–C9	1.383(5)	Y1'–N2'	2.320(3)
N4–C14	1.385(5)	Y2–N3	2.316(3)
N3'–C9'	1.383(5)	Y2–N4	2.322(3)
N4–C14	1.385(5)	Y2'–N3'	2.316(3)
**Phenyl C–C**		Y2'–N4'	2.322(3)
C2–C3	1.391(5)	**Atoms**	**Angles (°)**
C3–C4	1.397(5)	(Ph–Ph)1	0.1(1)
C4–C5	1.373(6)	(Ph–Ph)1	0.1(1)
C5–C7	1.389(5)	Y1–N1–N2'–Y1'	13.1(5)
C7–C6	1.401(5)	Y1–N2–N1'–Y1'	−13.1(5)
C6–C2	1.430(5)	Y2–N3–N4'–Y2'	−20.3(5)
C2'–C3'	1.391(5)	Y2–N4–N3'–Y2'	20.3(5)
C3'–C4'	1.397(5)	Cnt–Y1-Cnt	139.0(3)
C4'–C5'	1.373(6)	Cnt–Y1'-Cnt	139.0(3)
C5'–C7'	1.389(5)	Cnt–Y2-Cnt	137.3(3)
C7'–C6'	1.401(5)	Cnt–Y2'-Cnt	137.3(3)
C6'–C2'	1.430(5)		

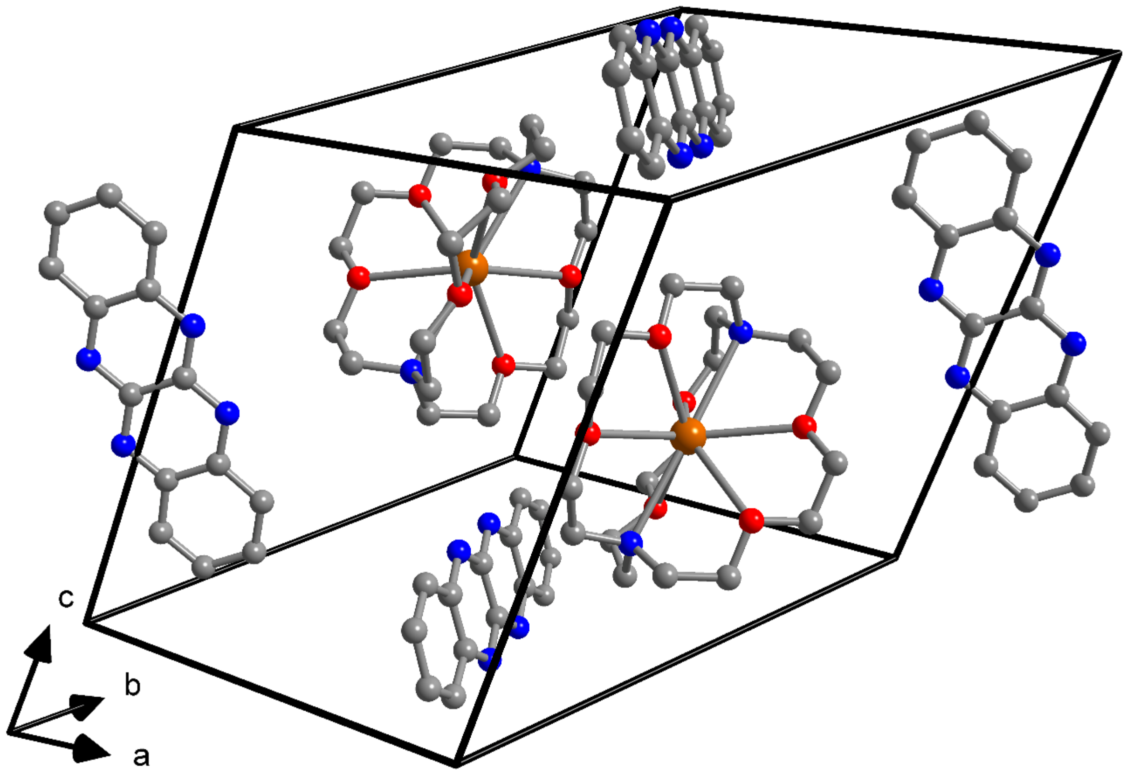

Figure S1. Unit cell of [K(crypt-222)](flv•) (**1**). Dark orange, red, blue, and gray spheres represent potassium, oxygen, nitrogen, and carbon atoms, respectively. All hydrogen atoms were omitted for clarity.

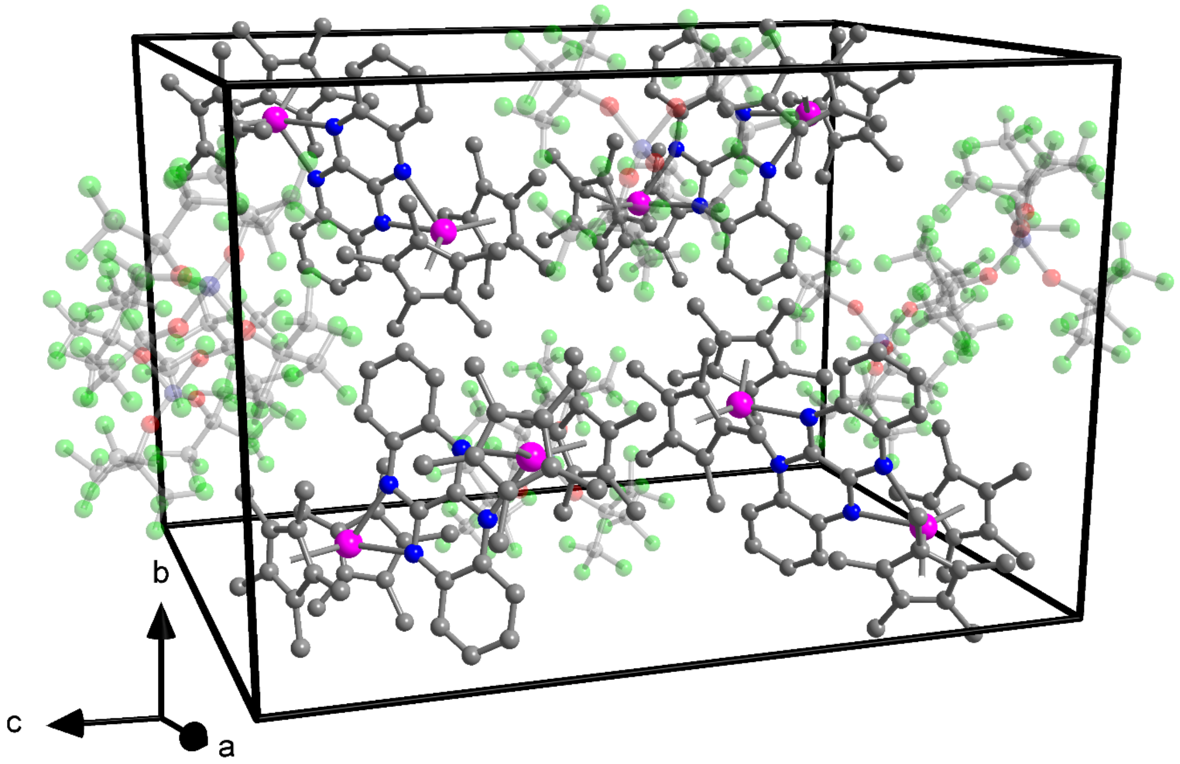

Figure S2. Unit cell of [(Cp*$_2$Y)$_2$(μ-flv•)][Al(OC{CF$_3$}$_3$)$_4$]·CH$_2$Cl$_2$ (**2**·CH$_2$Cl$_2$). Pink, blue, red, dark blue, green, and gray spheres represent yttrium, nitrogen, oxygen, aluminum, fluorine, and carbon atoms, respectively. All hydrogen atoms and solvent molecules are omitted, and the counter ions [Al(OC{CF$_3$}$_3$)$_4$]$^-$ are faded for clarity.

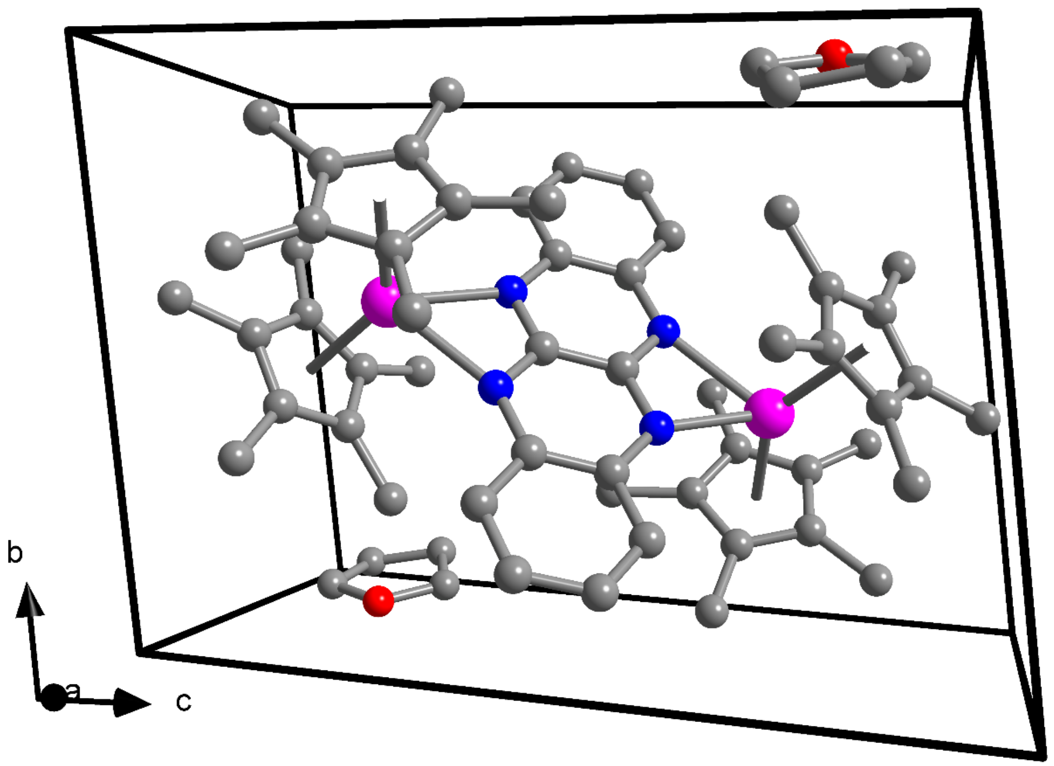

Figure S3. Unit cell of [(Cp*$_2$Y)$_2$(μ-flv)]·2THF (**3**·2THF). Pink, blue, red, and gray spheres represent yttrium, nitrogen, oxygen, and carbon atoms, respectively. All hydrogen atoms are omitted for clarity.

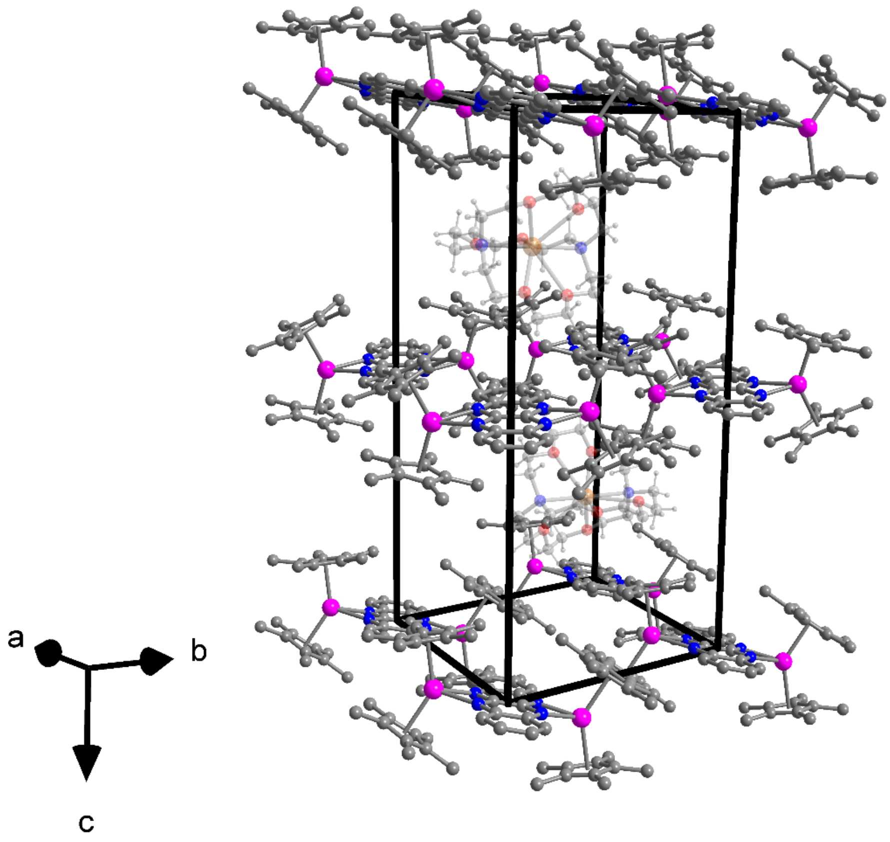

Figure S4. Unit cell of [K(crypt-222)][(Cp*$_2$Y)$_2$(μ-flv•)]·4THF (**4**·4THF). Pink, orange, red, blue, and gray spheres represent yttrium, potassium, oxygen, nitrogen, and carbon, atoms, respectively. All hydrogen atoms are omitted and the counter ions [K(crypt-222)]$^+$ are faded for clarity.

1.3 NMR Spectroscopy

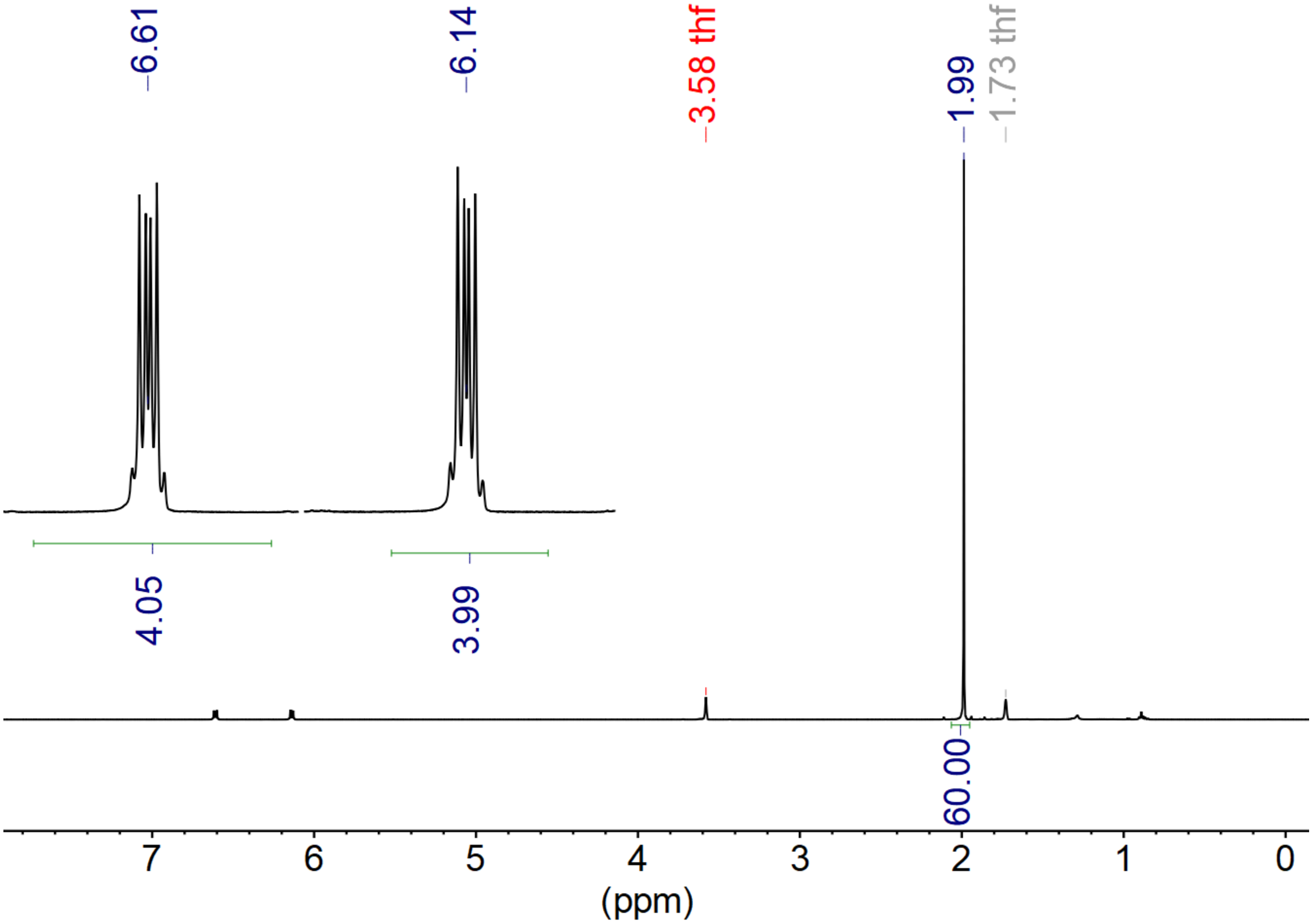

Figure S5. ^{1}H NMR spectrum of [(Cp*$_2$Y)$_2$(μ-flv)] (**3**) (500 MHz, THF-d_8, 25 °C).

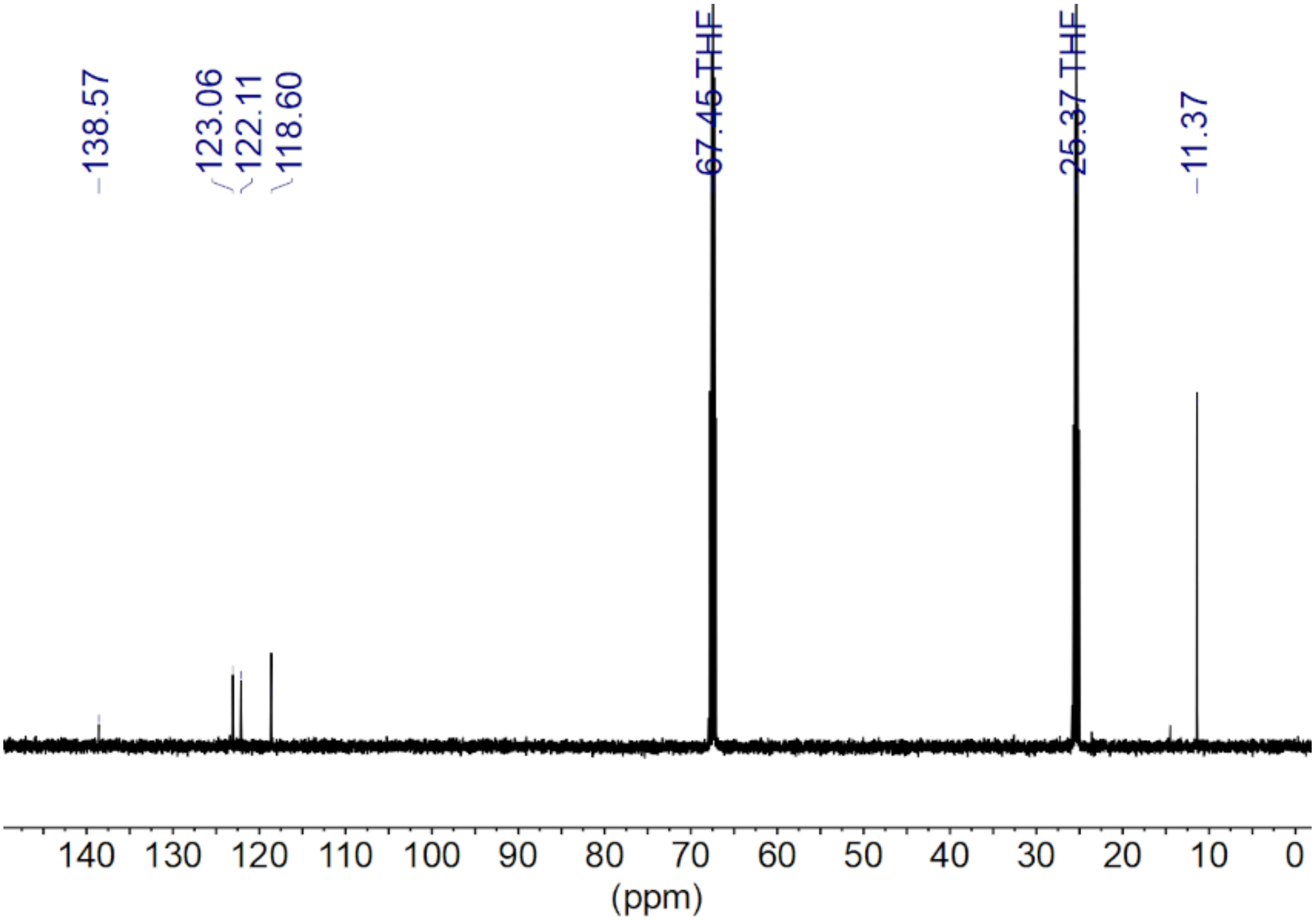

Figure S6. ^{13}C NMR spectrum of [(Cp*$_2$Y)$_2$(μ-flv)] (**3**) (125 MHz, THF-d_8, 25 °C).

1.4 EPR Spectroscopy

1.4.1 Details for Simulated VT EPR Spectra Depicted in Figure 11

Temperature-dependent EPR data were collected for compounds **1**, **2**, and **4** to evaluate hyperfine couplings at different temperatures. Due to divergent chemical stabilities, the radical complexes **2** and **4** were collected in difluorobenzene and THF solutions, respectively, giving rise to divergent accessible temperature windows (THF: 165-339 K; difluorobenzene: 239-365 K). Consequently, cw EPR spectra of samples **1** and **4** were collected between 320 K and 170 K in 6 steps, and of sample **2** between 320 K and 240 K in 8 steps.

Variable-temperature EPR spectra of [K(crypt-222)](flv•) (**1**) were measured as a THF solution (0.88 mmol/L). The experimental spectra were recorded at 9.324 GHz. The simulated spectra were obtained with parameters: 4 ^{14}N, 4 $^{1}H_1$, and 4 $^{1}H_2$ nuclei, $A(^{14}N)$ = 8.10 MHz, $A_1(^{1}H)$ = 4.25 MHz, $A_2(^{1}H)$ = 2.50 MHz, *g* = 2.00355, linewidth: 0.033 mT. Linewidths at other temperatures were kept at 0.033 mT.
Data were collected with an attenuation of 12.0 dB (12.62 mW) and a modulation amplitude of 0.100 G.

The variable-temperature EPR spectra of $[(Cp^*_2Y)_2(\mu\text{-flv}\bullet)][Al(OC\{CF_3\}_3)_4]$ (**2**) were measured in difluorobenzene solution (0.67 mmol/L). The experimental spectra were recorded at 9.324 GHz. The simulated spectra were obtained with parameters: 4 ^{14}N, 4 $^{1}H_1$, 4 $^{1}H_2$ and 2 ^{89}Y nuclei, $A(^{14}N)$ = 7.90 MHz, $A_1(^{1}H)$ = 4.50 MHz, $A_2(^{1}H)$ = 1.30 MHz, $A(^{89}Y)$ = 3.90 MHz *g* = 2.003175, linewidth: 0.07 mT. Other temperatures: **320 K:** *g* = 2.003195, LW: 0.07 mT; **310 K:** *g* = 2.003195, LW: 0.075 mT; **280 K:** *g* = 2.003195, LW: 0.08 mT; **270 K:** *g* = 2.003195, LW: 0.085 mT; **260 K:** *g* = 2.003195, LW: 0.095 mT; **250 K:** *g* = 2.003195, LW: 0.1 mT; **240 K:** *g* = 2.003195, LW: 0.115 mT.
Data were collected with an attenuation of 12.0 dB (12.62 mW) and a modulation amplitude of 0.100 G.

The variable-temperature EPR spectra of $[K(crypt\text{-}222)][(Cp^*_2Y)_2(\mu\text{-flv}\bullet)]$ (**4**) were measured in THF solution (0.22.mmol/L). The experimental spectra were recorded at 9.335 GHz. The simulated spectra were obtained with parameters: 4 ^{14}N, 4 $^{1}H_1$, 4 $^{1}H_2$ and 2 ^{89}Y nuclei, $A(^{14}N)$ = 5.85 MHz, $A_1(^{1}H)$ = 2.25 MHz, $A_2(^{1}H)$ = 1.0 MHz, $A(^{89}Y)$ = 1.00 MHz *g* = 2.0049, linewidth: 0.025 mT. Other temperatures: **320 K:** *g* = 2.00485, LW: 0.017 mT; **280 K:** *g* = 2.004855, LW: 0.035 mT; **240 K:** *g* = 2.00486, LW: 0.035 mT; **210 K:** *g* = 2.0055, LW: 0.04 mT; **170 K:** *g* = 2.0052, LW: 0.05 mT.
Data were collected with an attenuation of 33.0 dB (0.1001 mW) and a modulation amplitude of 0.07 G.

1.4.2 Variable-Temperature EPR Intensity Analysis of Flv Radicals

Since in EPR spectroscopy, the population of Zeeman states obeys Boltzmann's law, and the signal intensity hinges on the population difference between ground and excited state, a decrease in temperature should result in an increased signal intensity in the absence of any intramolecular interactions or changes in relaxation behavior. Hence, the study of the temperature dependence of a paramagnetic species in solution may unfold the presence of intramolecular processes such as dynamic dimerization via π-stacking. In this scenario, EPR inactive π-dimers would form which unequivocally lead to an intensity loss, as for example demonstrated for the recently published olympicenyl, phenalenyl, and other radical species, respectively.[36–38] The opposite behavior has recently been reported for actinide complexes bridged by two radical hexaazatrinaphthalene ($HAN^{3-\bullet}$) ligands bearing intramolecular pancake bonds.[39]

Consequently, we were interested to investigate how the charge of the flv bridging ligand would affect potential intermolecular dimerization in solution.

Generally, the intensity of an EPR spectrum as obtained via double integration (DI) of the derivative spectra is given by:[40]

$$DI = \frac{c}{f(B_1,B_m)}\{G_R C_t n\}\{\sqrt{P} B_m Q n_B S(S+1) n_S \qquad (1)$$

where:

c = Point sample calibration factor

B_1 = Microwave magnetic field

B_m = Modulation amplitude

$f(B_1, B_m)$ = Spatial distribution of $B_1$and B_m

G_R = Receiver gain

C_t = Conversion time

n = Number of averages

$\sqrt{P}$ = Square root of the microwave power

Q = Quality factor of the resonator

n_B = Boltzmann factor to correct for temperature

S = Electron spin

n_S = Number of spin

Since not all these parameters are known, particularly Q, which would be required to directly calculate the number of spins from a certain DI, a relative measurement against a standard sample can be carried out. Here, if all parameters are identical for standard and analyte, the ratio of unknown sample DI to standard DI can be used to obtain a relative spin count:

$$\frac{n_{s\,unknown}}{n_{s\,standard}} = \frac{DI_{unknown}}{DI_{standard}} = \chi_{EPR} \qquad (2)$$

The corrected EPR intensities can then be used for a quantitative assessment of the intermolecular interactions between paramagnetic molecules, where the variable-temperature can be fit to a Bleany-Bowers equation:[41]

$$\chi_{EPR} = \frac{C}{T}\frac{3}{3+exp\left(\frac{T_{ST}}{T}\right)} \qquad (3)$$

where C is a constant to the sample intensity and T_{ST} is the exchange temperature defining the singlet-triplet gap. If dimerization occurs among paramagnetic species to form singlet ground state assembly, a downward curvature in χ_{EPR} vs. T plots will become apparent.

Intensity studies for the variable-temperature EPR spectroscopy were carried out via the following procedure: Starting at room temperature, the samples were initially cooled to the lowest temperatures (230 K (MeCN), 170 K (THF) and 240 K (DFB)) and kept at these temperatures for 10 minutes to ensure proper thermal equilibration of the spins. The EPR spectra were then recorded as the average of 5 individual spectra, and subsequently warmed to the next temperatures. A waiting period of 10 min was maintained at each subsequent temperature.

To ensure intensity studies were not impacted by saturation, for each sample three attenuations were initially probed at lowest temperature, of which the highest non-saturating attenuation (lowest microwave power) was chosen for the VT EPR experiments.

For each sample, an appropriate reference sample of TEMPONE was prepared and measured of the same concentration as the analyte (0.62 mmol/L in each case). Analyte and TEMPONE samples were measured in 2 mm quartz EPR tubes except for **4**, which could not be transferred via metal syringe without immediate decomposition. Instead, **4** was measured in a 3 mm Quartz EPR tube that could be filled with a glass pipette.

EPR spectra were recorded with a modulation amplitude of 2 G for each sample. The EPR intensities were obtained from the experimental spectra by applying a baseline correction via first order polynomial subtraction, and subsequent double integration of the baseline-corrected spectra over a 100 G region (3280-3380 G). Experimental spectra are provided in Figure S7-Figure S11.

After correcting the obtained EPR intensities for the respective TEMPONE intensities it appears as most species remain unaffected by neighboring paramagnetic molecules in solution within the probed temperature regime: For the free $flv^{1-\bullet}$ radical in [K(crypt-222)](flv•) (**1**), no significant increase or decrease in intensity could be observed in either of the three probed solvents THF, difluorobenzene, and acetonitrile, which would otherwise indicate either ferromagnetic or antiferromagnetic interaction among molecules (Figure S7-Figure S9 and Figure S12). Similarly, for the yttrium coordinated $flv^{1-\bullet}$ radical in $[(Cp^*{}_2Y)_2(\mu\text{-flv}^\bullet)][Al(OC\{CF_3\}_3)_4]$ (**2**) (DFB) no significant changes were observed, as anticipated due to the considerable steric shielding of the flv radical

ligands provided by the flanking bis-Cp* coordination environment and preventing close intermolecular interactions (Figure S13).

Lastly, the intensity profile of the $flv^{3-\bullet}$ radical in [K(crypt-222)][$(Cp^*{}_2Y)_2$(μ-flv•)] (**4**) was probed in THF. Here, a slight increase is observed upon warming from 170 K, reaching a maximum at 230 K and then a gradual decrease is monitored upon approaching room temperature. This slight initial increase in intensity upon warming is hypothesized not to correlate with π-dimer formation but instead arises from instrumentation conditions.

Since none of the intensity curves showed the expected clear deviation from linearity at lower temperatures, we refrained from employing the Bleany-Bowers fits for this dataset.

To conclude, all compounds investigated do not show characteristics of π-dimerization in solution.

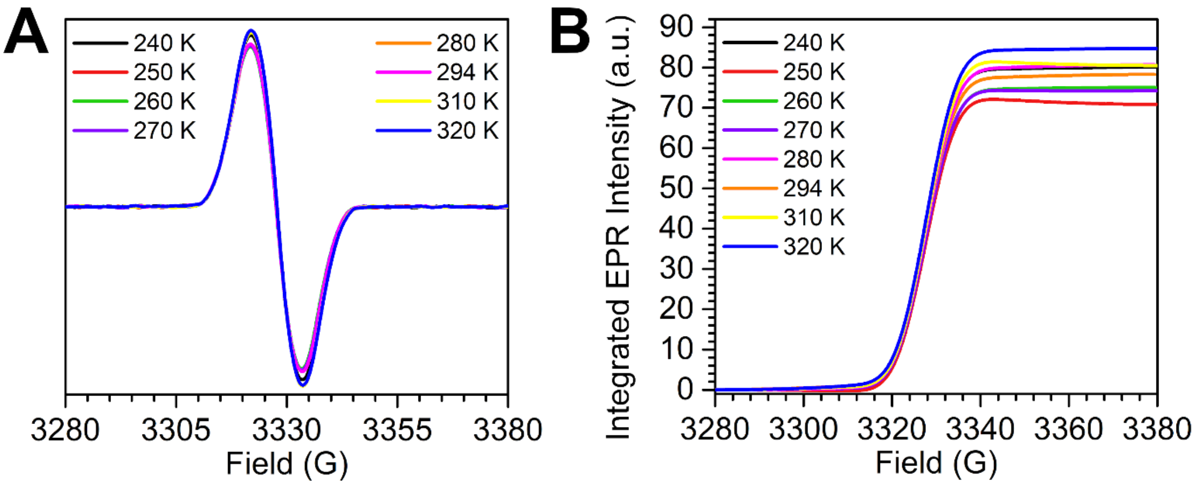

Figure S7. Baseline-corrected EPR spectra collected for the variable-temperature intensity study of [K(crypt-222)](flv•) (**1**) in 1,2-difluorobenzene between 240 K and 320 K (left). Results of the double integration of the VT EPR spectra of **1** collected in 1,2-difluorobenzene (right).

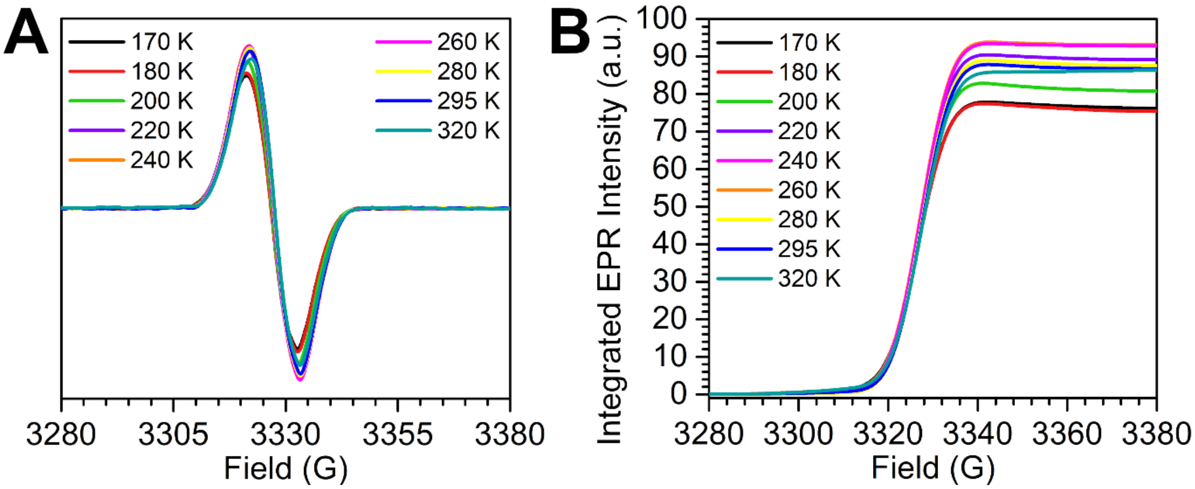

Figure S8. Baseline-corrected EPR spectra collected for the variable-temperature intensity study

of [K(crypt-222)](flv•) (**1**) in THF between 170 K and 320 K (left). Results of the double integration of the VT EPR spectra of **1** collected in THF (right).

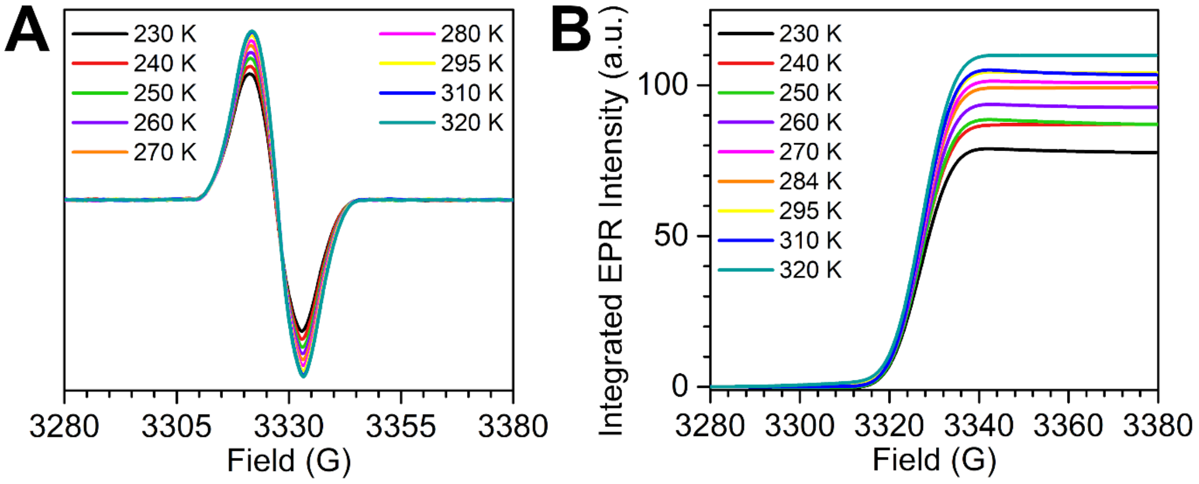

Figure S9. Baseline-corrected EPR spectra collected for the variable-temperature intensity study of [K(crypt-222)](flv•) (**1**) in acetonitrile between 230 K and 320 K (left). Results of the double integration of the VT EPR spectra of **1** collected in acetonitrile (right).

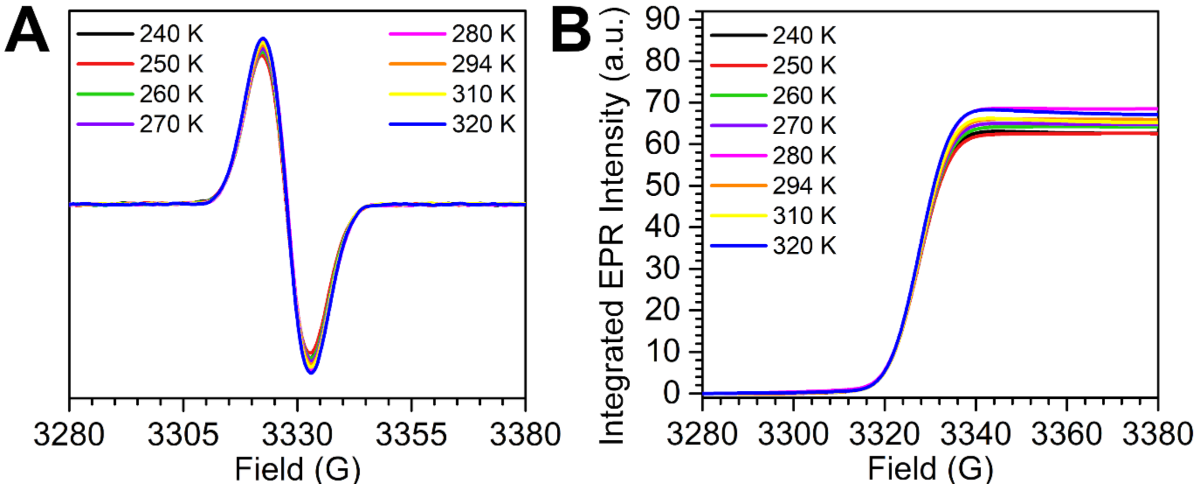

Figure S10. Baseline-corrected EPR spectra collected for the variable-temperature intensity study of [(Cp*$_2$Y)$_2$(μ-flv•)][Al(OC{CF$_3$}$_3$)$_4$] (**2**) in 1,2-difluorobenzene between 240 K and 320 K (left). Results of the double integration of the VT EPR spectra of **2** collected in 1,2-difluorobenzene (right).

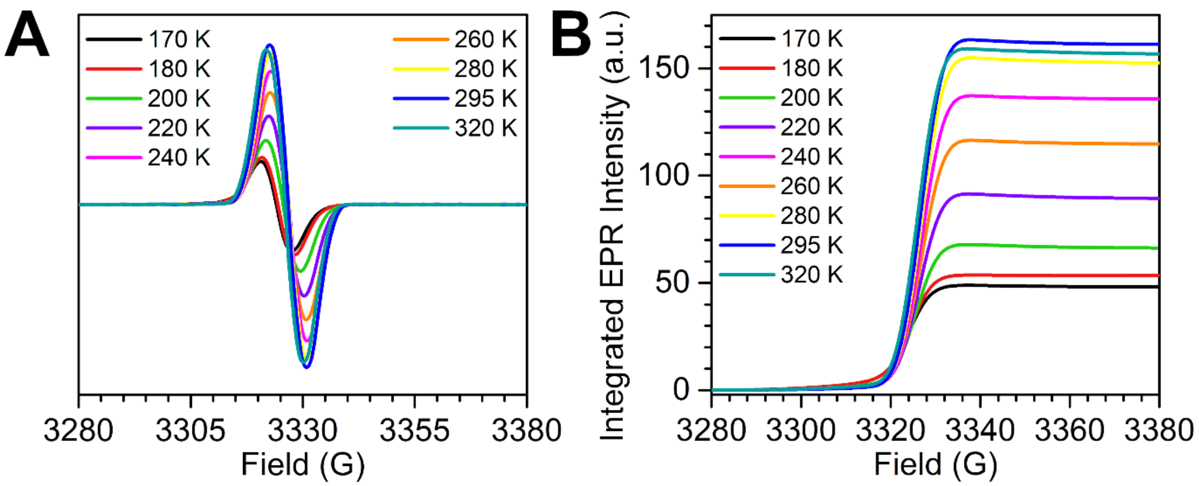

Figure S11. Baseline-corrected EPR spectra collected for the variable-temperature intensity study of [K(crypt-222)][$(Cp^*{}_2Y)_2$(μ-flv•)] (**4**) in THF between 170 K and 320 K (left). Results of the double integration of the VT EPR spectra of **4** collected in THF (right).

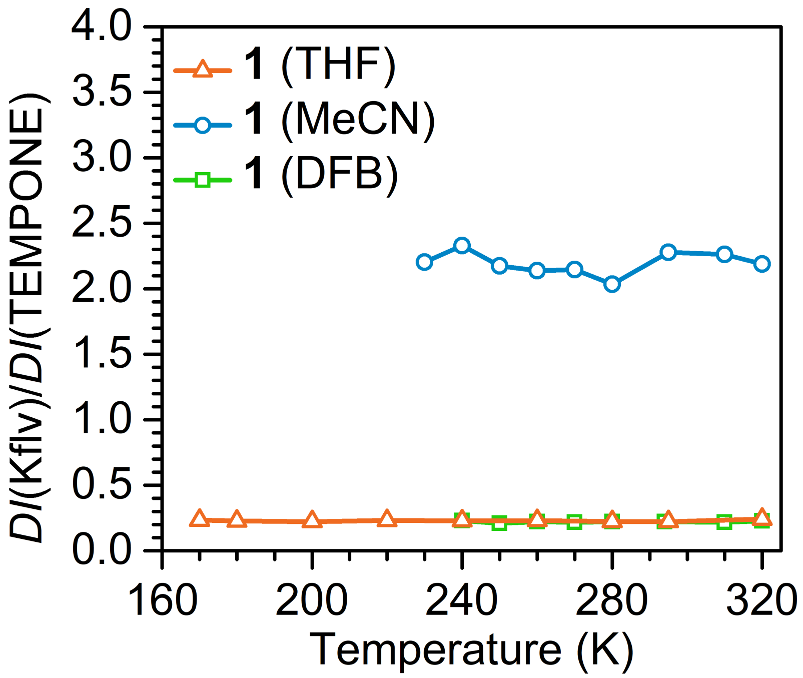

Figure S12. EPR-integrated intensity as a function of the temperature for K(crypt-222)](flv•) (**1**) in THF (orange triangles), acetonitrile (blue circles), and 1,2-difluorobenzene (green squares). Colored lines are guides for the eye.

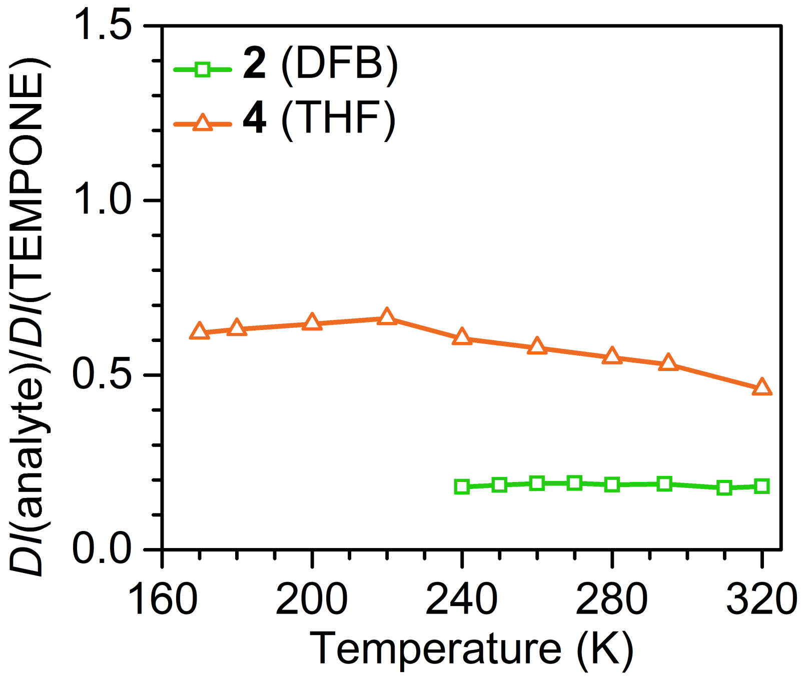

Figure S13. EPR-integrated intensity as a function of the temperature for [K(crypt-222)][(Cp*$_2$Y)$_2$(μ-flv•)] (**4**) in THF (orange triangles) and [(Cp*$_2$Y)$_2$(μ-flv•)][Al(OC{CF$_3$}$_3$)$_4$] (**2**) in 1,2-difluorobenzene (green squares). Colored lines are guides for the eye. While for **4** a slight increase in intensity upon warming can be observed (0.62 a.u. at 170 K to 0.66 a.u.at 220 K), this is hypothesized not to correlate with π-dimer formation but instead arises from instrumentation conditions.

1.5 IR Spectroscopy

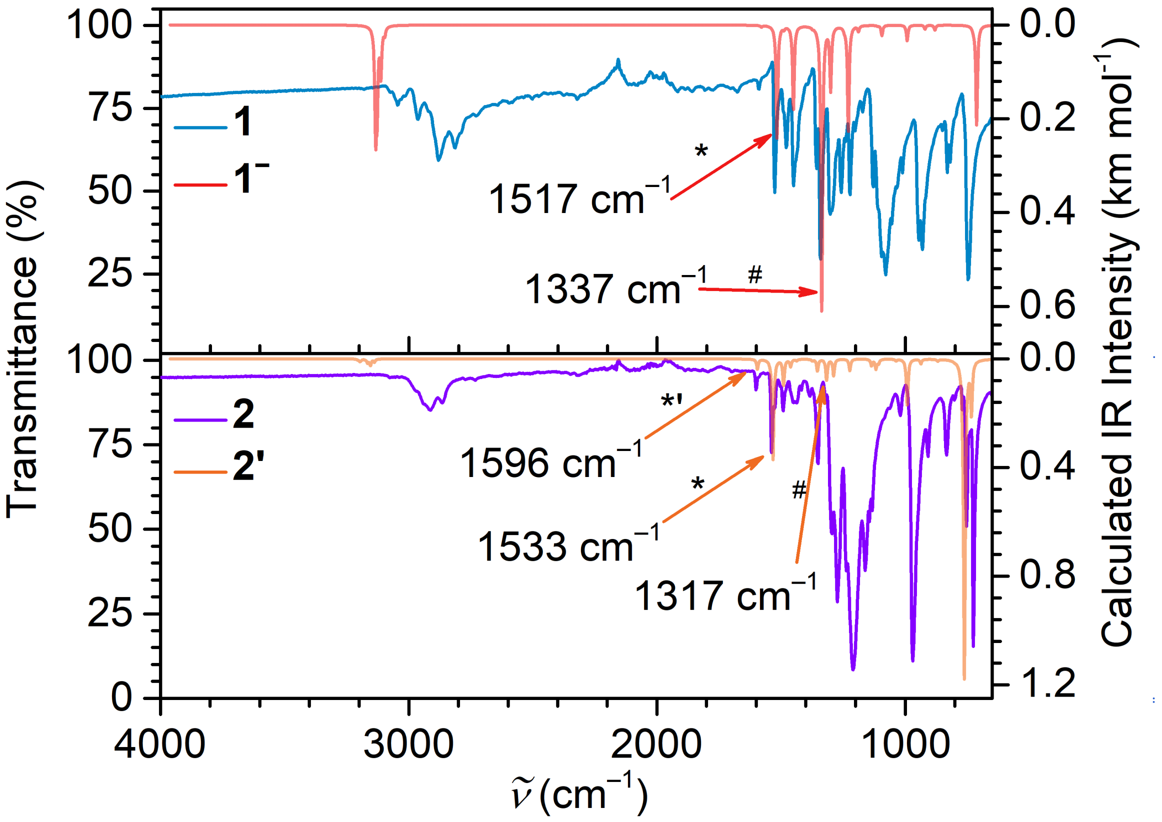

Figure S14. Top: IR spectra of [K(crypt-222)](flv•) (**1**, blue) recorded on polycrystalline solids, superimposed with calculated spectrum for the $flv^{1-•}$ anion in $\mathbf{1}^-$ (red). Bottom IR spectra of $[(Cp^*{}_2Y)_2(\mu\text{-flv•})][Al(OC\{CF_3\}_3)_4]$, (**2**, purple) recorded on polycrystalline solids, superimposed with calculated spectrum for **2'** (orange). **2'** represents a contracted model of **2** where all Cp* methyl groups were substituted with protons, $[(Cp_2Y)_2flv•]^+$. Arrows indicate characteristic flv-based vibrations: * = Antisymmetric lateral flv Ph wagging; *' = Antisymmetric longitudinal flv stretch; # = Antisymmetric lateral stretch of central C–C bonds. Calculated spectra were generated through application of an 8 cm^{-1} Lorentzian line broadening. Calculated vibrations were empirically shifted by −38 cm^{-1} for a better match with the experimental spectra.

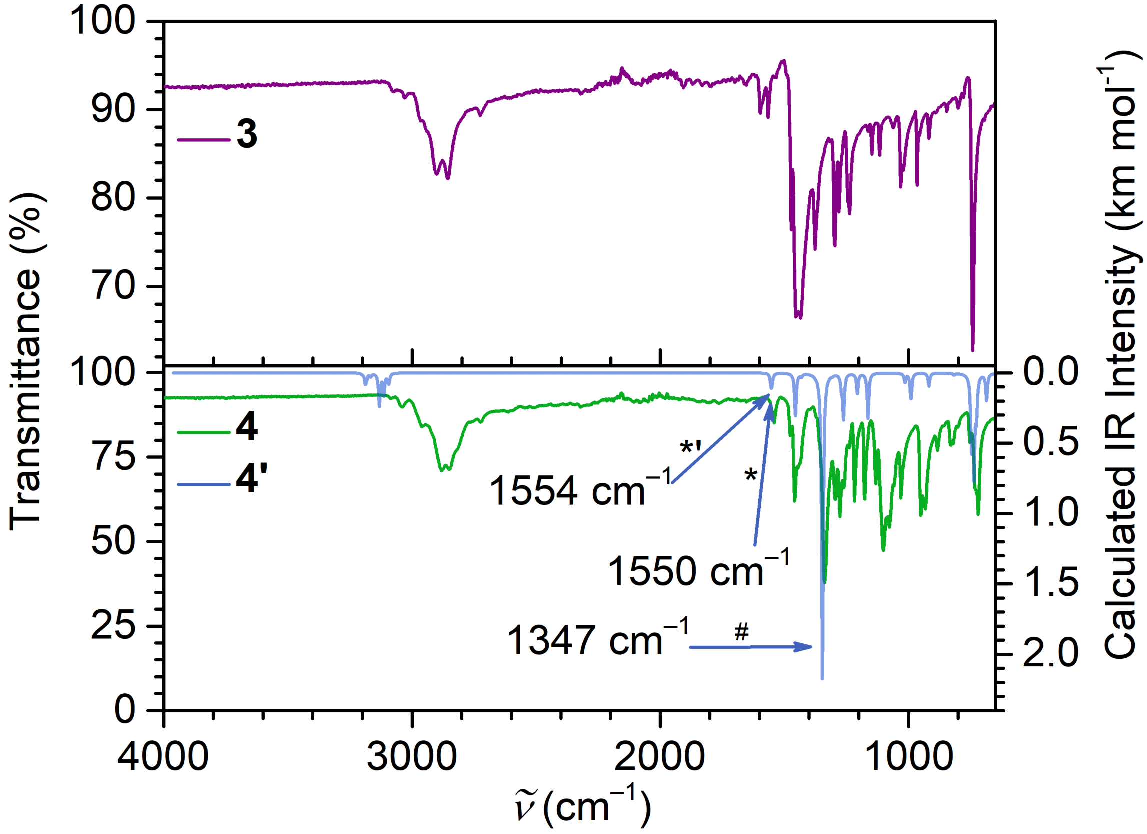

Figure S15. Top: IR spectra of $[(Cp^*_2Y)_2(\mu\text{-flv})]$ (**3**, purple) recorded on polycrystalline solids. Bottom IR spectra of $[K(crypt\text{-}222)][(Cp^*_2Y)_2(\mu\text{-flv}^\bullet)]$ (**4**, green) recorded on polycrystalline solids, superimposed with calculated spectrum for **4'** (blue). **4'** represents a contracted model of **4** where all Cp* methyl groups were substituted with protons, $[(Cp_2Y)_2flv^\bullet]^-$. Arrows indicate characteristic flv-based vibrations: *' = Antisymmetric longitudinal flv stretch; * = Antisymmetric lateral flv Ph wagging; # = Asymmetric lateral stretch of central C–C bonds. Calculated spectra were generated through application of an 8 cm^{-1} Lorentzian line broadening. Calculated vibrations were empirically shifted by −38 cm^{-1} for better match with the experimental spectra.

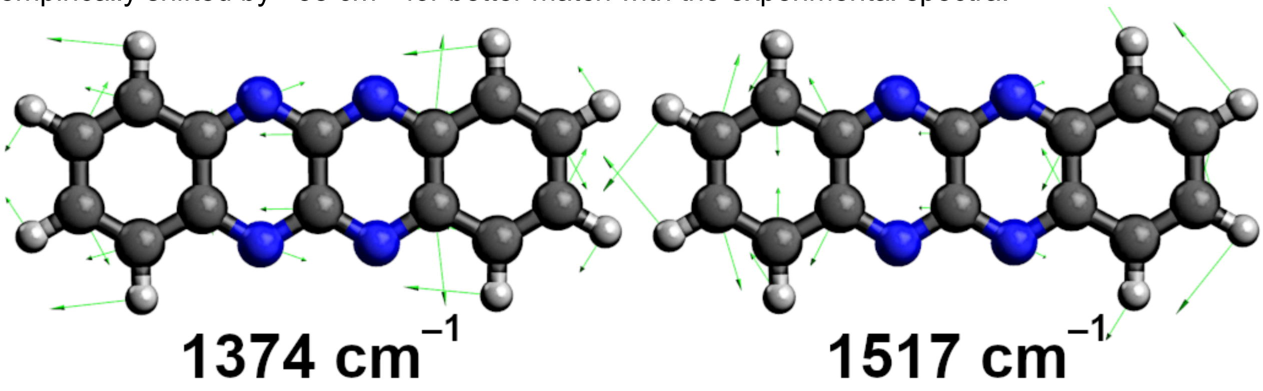

Figure S16. Visualization of core vibrations highlighted in Figure S14 of $flv^{1-\bullet}$ ($\mathbf{1}^-$).

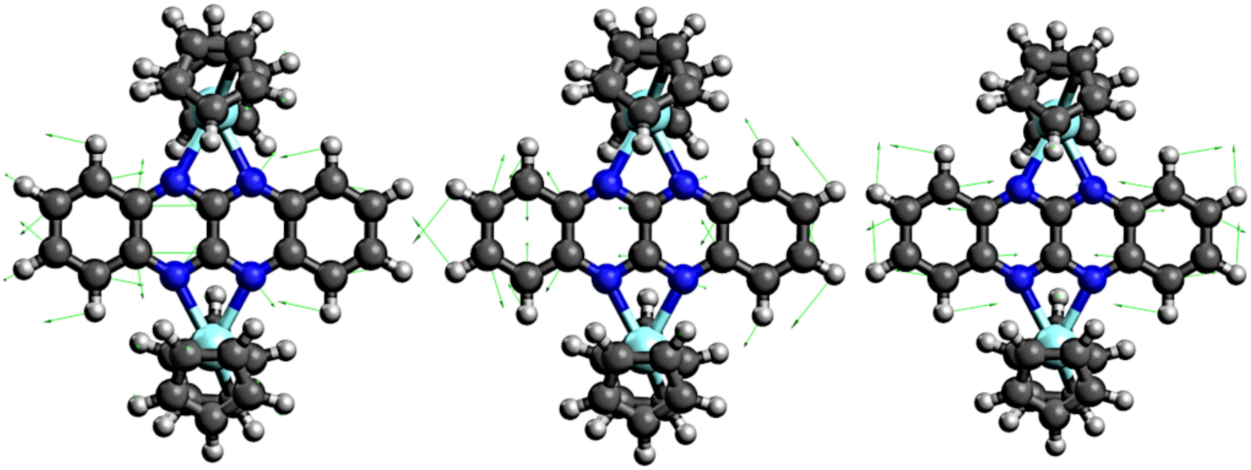

1317 cm^{-1} 1533 cm^{-1} 1596 cm^{-1}

Figure S17. Visualization of core vibrations highlighted in Figure S14 of $[(Cp_2Y)_2(\mu\text{-flv}\cdot)]^+$ (**2'**).

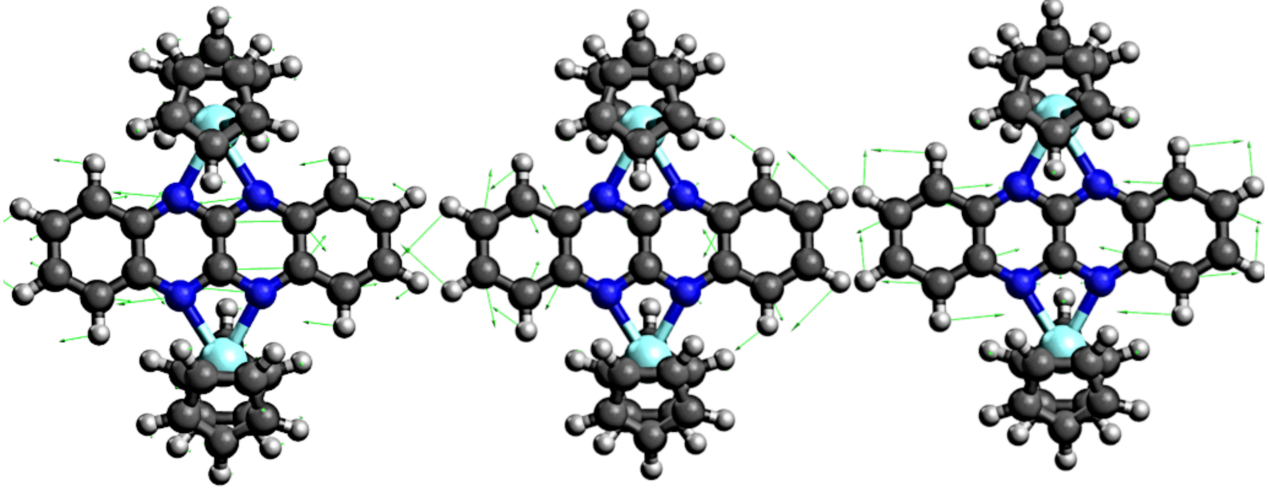

1347 cm^{-1} 1550 cm^{-1} 1554 cm^{-1}

Figure S18. Visualization of core vibrations highlighted in Figure S15 of $[(Cp_2Y)_2(\mu\text{-flv}\cdot)]^-$ (**4'**).

1.6 UV-Vis Spectroscopy

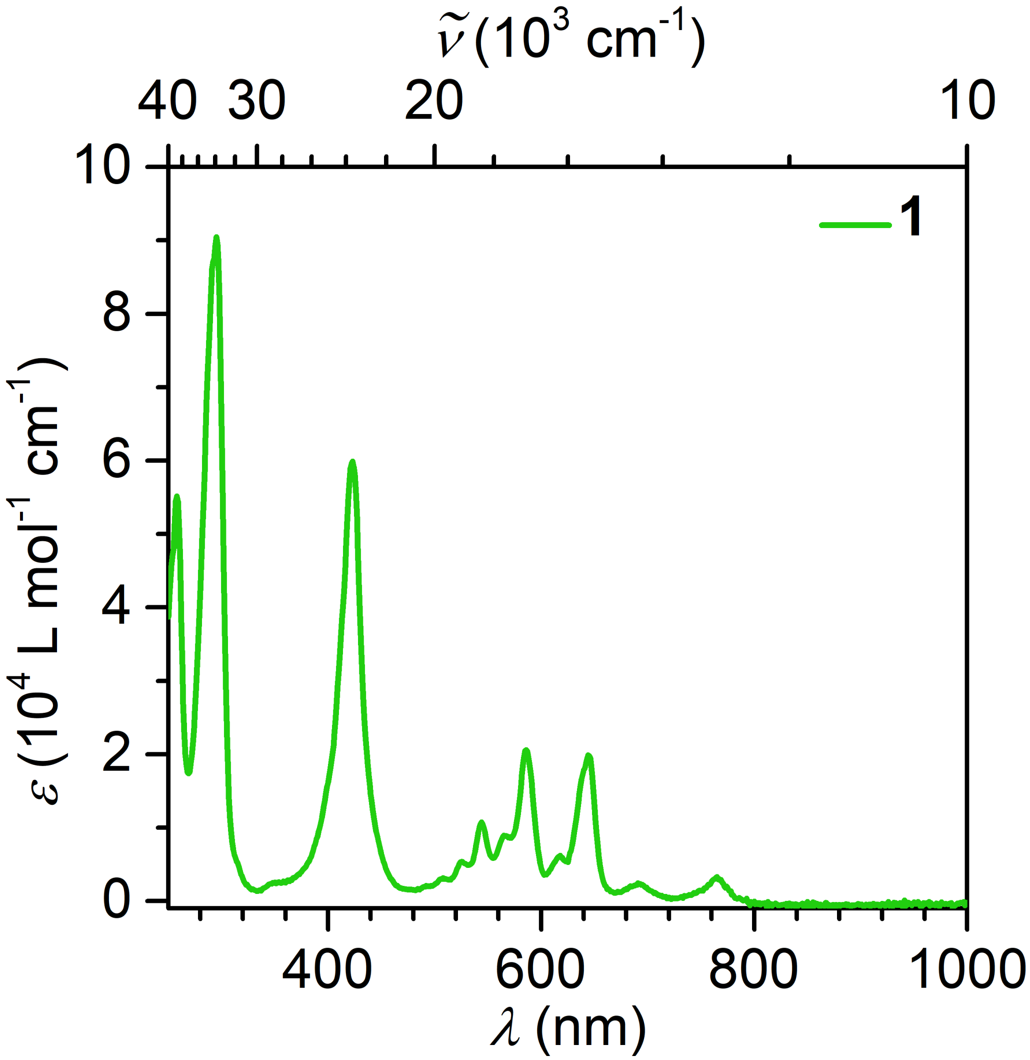

Figure S19. Experimental UV-Vis spectrum of [K(crypt-222)](flv$^{\bullet}$) (**1**), measured as a 19.29 µmol/L solution in THF.

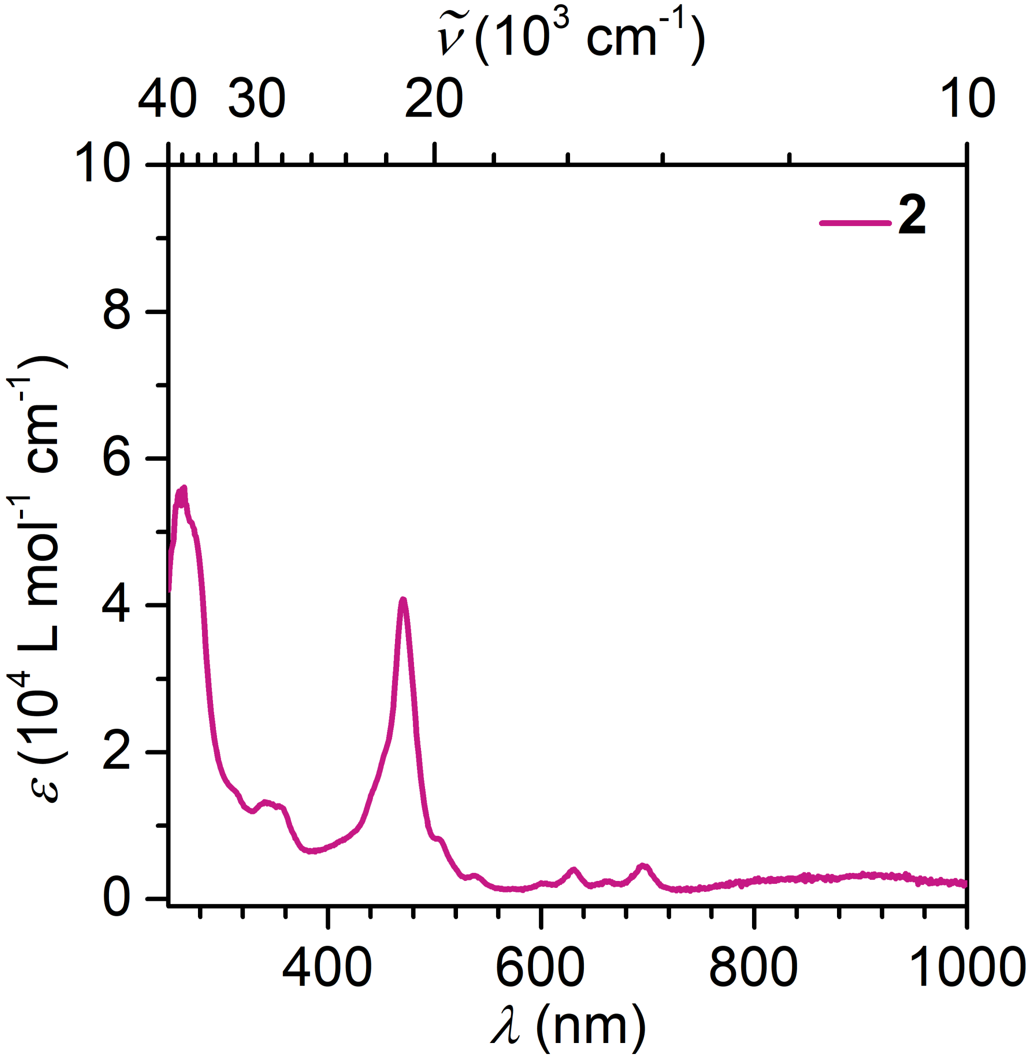

Figure S20. Experimental UV-Vis spectrum of [(Cp*$_2$Y)$_2$(μ-flv•)][Al(OC{CF_3}$_3$)$_4$] (**2**), measured as a 19.95 μmol/L solution in DCM.

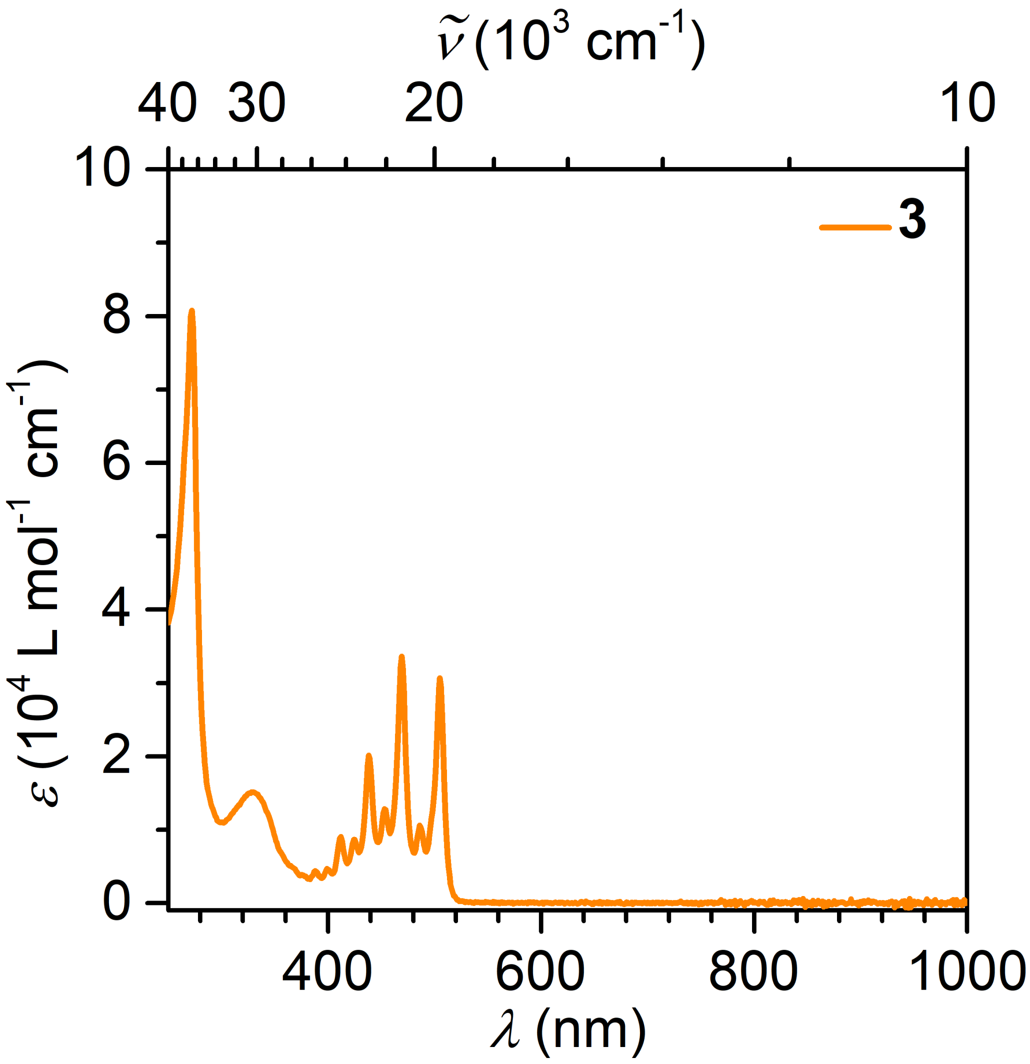

Figure S21. Experimental UV-Vis spectrum of [(Cp*$_2$Y)$_2$(μ-flv)] (**3**), measured as a 11.16 μmol/L solution in THF.

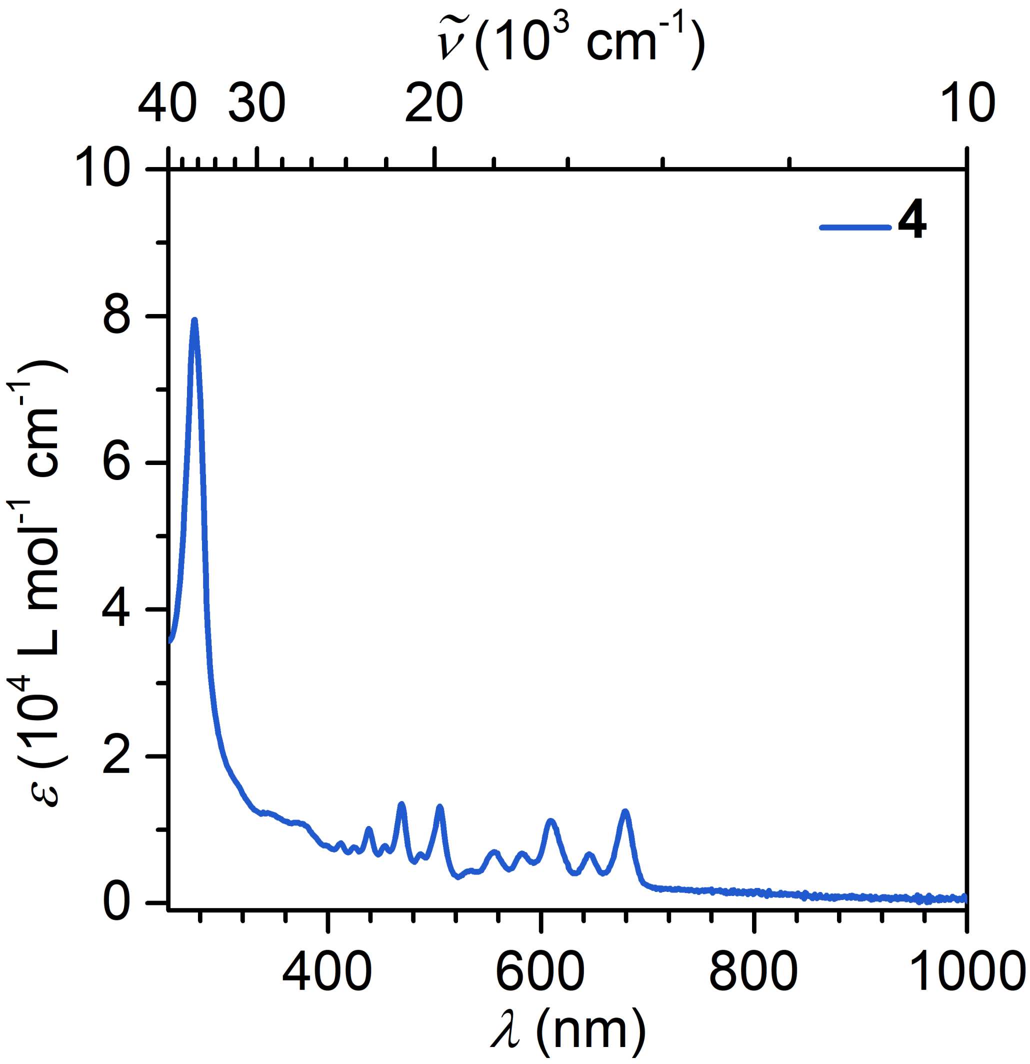

Figure S22. Experimental UV-Vis spectrum of [K(crypt-222)][(Cp*$_2$Y)$_2$(μ-flv•)] (**4**), measured as a 14.20 μmol/L solution in THF.

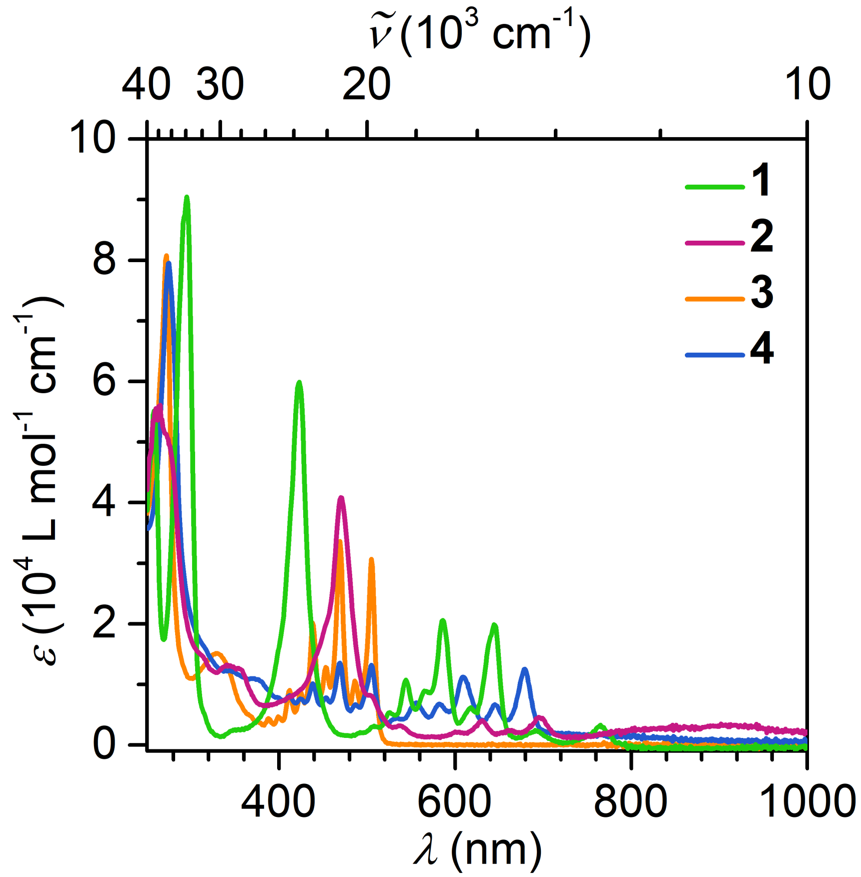

Figure S23. Superimposed experimental UV-Vis spectra of [K(crypt-222)](flv•) (**1**), [(Cp*$_2$Y)$_2$(μ-flv•)][Al(OC{CF$_3$}$_3$)$_4$] (**2**), [(Cp*$_2$Y)$_2$(μ-flv)] (**3**), [K(crypt-222)][(Cp*$_2$Y)$_2$(μ-flv•)] (**4**). Experimental concentrations: **1** (19.29 μmol/L in THF), **2** (19.95 μmol/L in DCM), **3** (11.16 μmol/L in THF), **4** (14.20 μmol/L in THF).

1.7 Cyclic Voltammetry

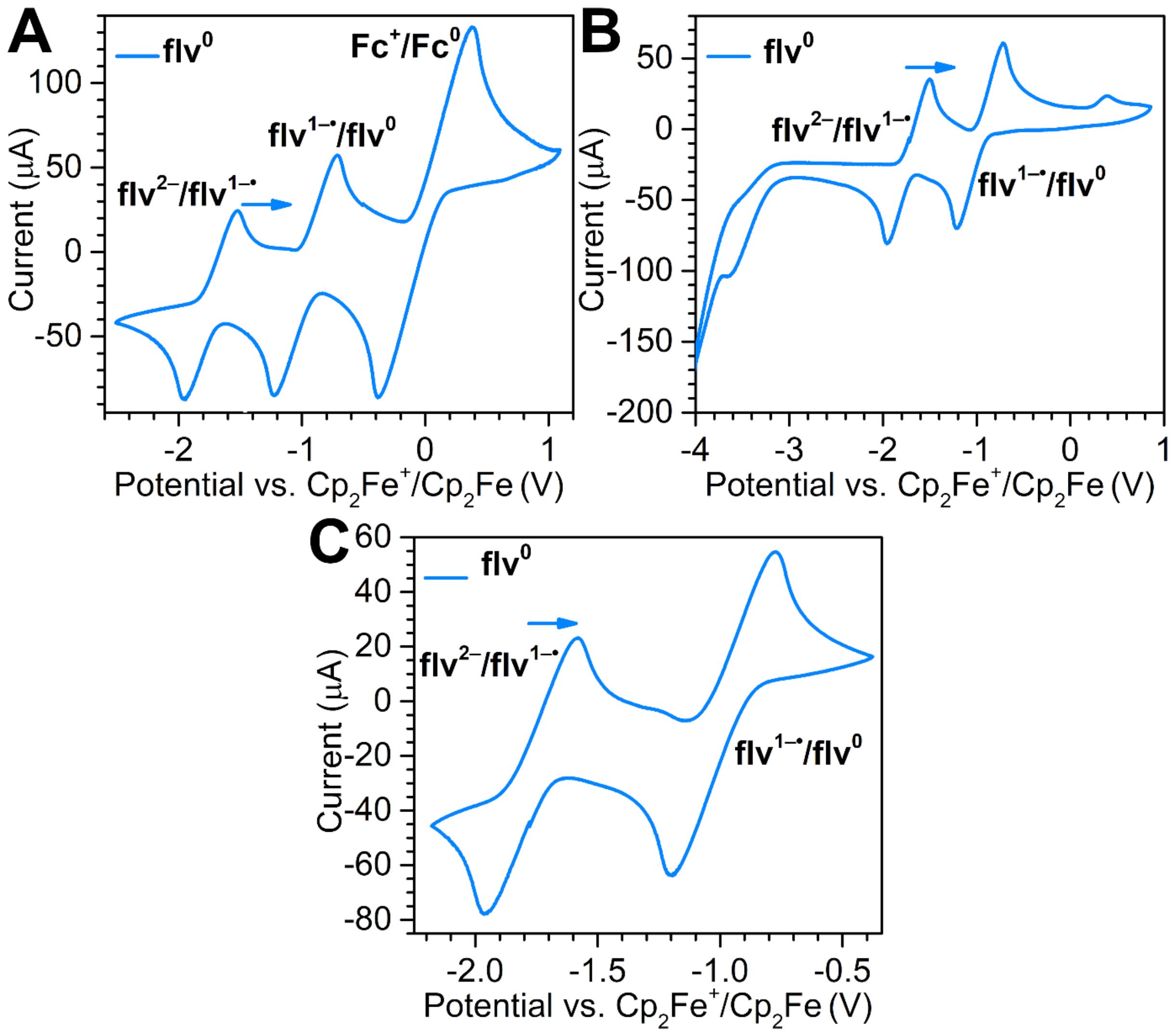

Figure S24. Full cyclic voltammograms (**A** and **B**) and magnification (**C**) of the reversible processes of flv^0, measured in THF. To elucidate the redox chemistry of flv^0, cyclic voltammetry (CV) was performed in THF solution using (^{n}Bu$_4$N)PF$_6$ as the supporting electrolyte. In accordance to previously reported CV data,[4,42] two quasi reversible reduction features were observed at −1.73(3) V and −0.96(2) V vs. ferrocene/ferrocenium redox couple. These potentials are more negative relative to the reported values of −0.80 V and −1.34 V,[4] likely originating from different electrolyte concentration and the use of acetonitrile as solvent.

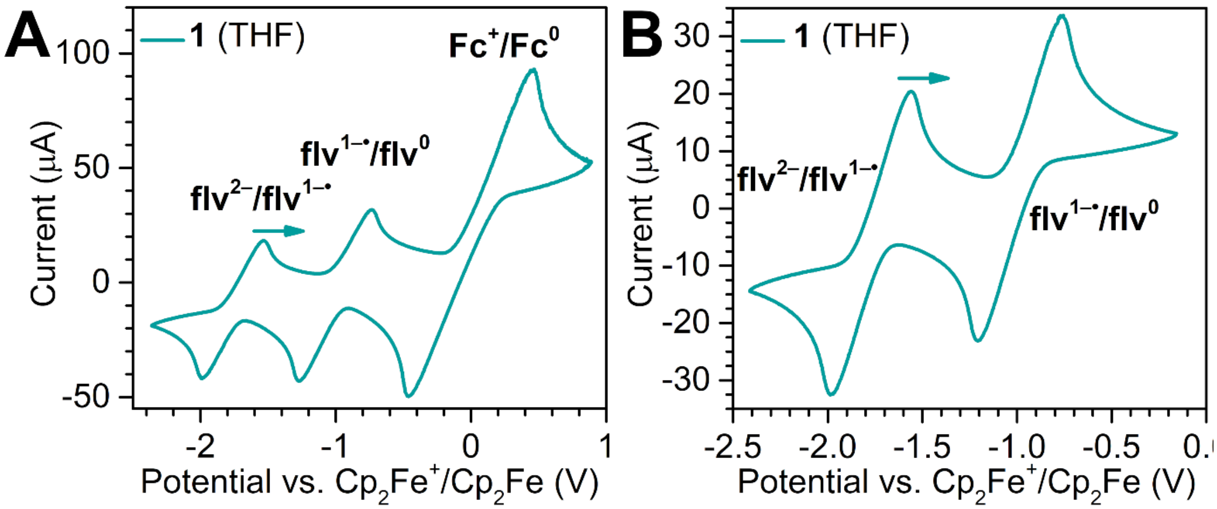

Figure S25. (**A**) Full cyclic voltammogram of [K(crypt-222)](flv•) (**1**), measured in THF. (**B**) Magnification of the reversible redox features. Notably, these redox features align perfectly with the features measured for flv^0 in Figure S24.

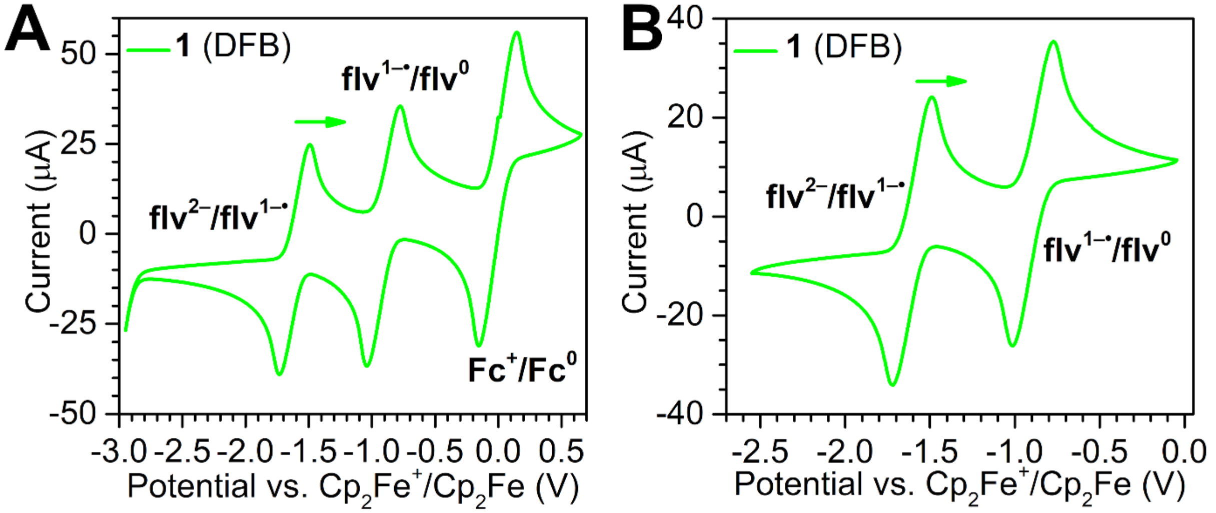

Figure S26. (**A**) Full cyclic voltammogram of [K(crypt-222)](flv•) (**1**), measured in 1,2-difluorobenzene. (**B**) The magnification of the reversible redox features is also part of main text Figure 4.

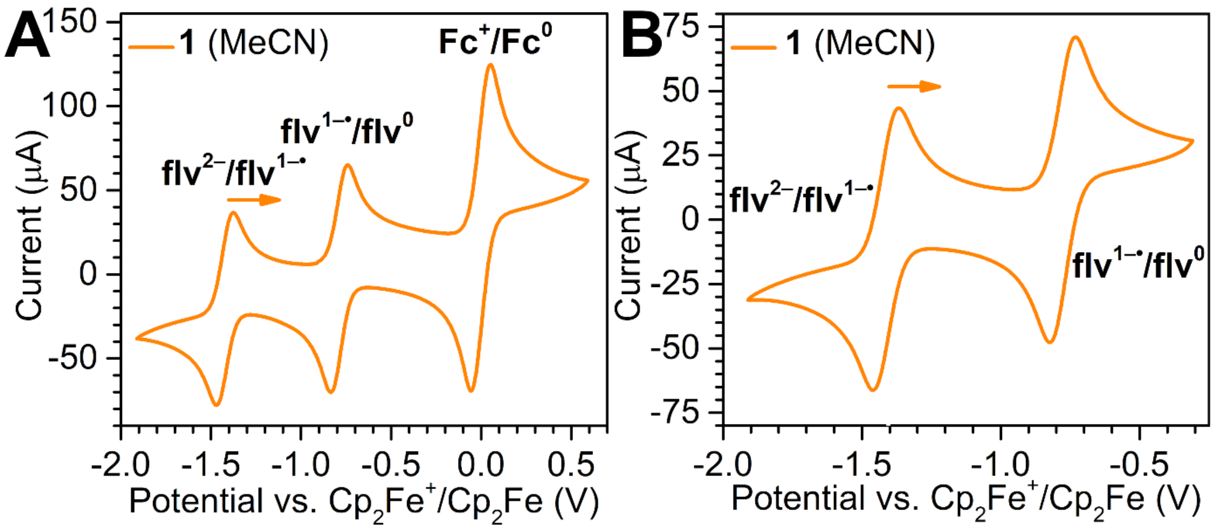

Figure S27. (**A**) Full cyclic voltammogram of [K(crypt-222)](flv•) (**1**), measured in acetonitrile. (**B**) Magnification of the reversible redox features.

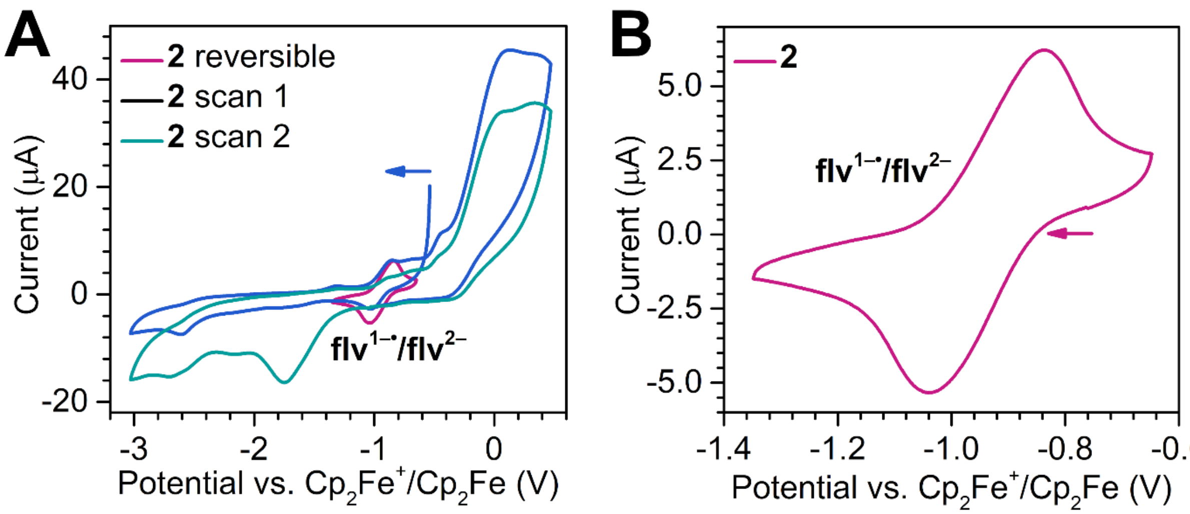

Figure S28. (**A**) Full cyclic voltammogram of [(Cp*$_2$Y)$_2$(μ-flv•)][Al(OC{CF$_3$}$_3$)$_4$] (**2**), measured in 1,2-difluorobenzene. (**B**) Magnification of the reversible redox feature is also depicted in Figure 4 in the main text. Notably, an initial reduction feature at ~ −1.0 V is observed, which is recovered at ~ −0.87 V following a scan towards −3.0 V. Upon scanning towards positive potentials, a strongly irreversible redox wave emerges at ~ −0.09 V. The subsequent sweep towards negative potentials shows no reduction feature between −0.5 and −1.5 V that could be correlated with the initial reduction process, indicating the irreversible oxidation and decomposition of **2**. Narrow potential measurements (in purple) around the initial redox feature reveal a pseudo reversible feature associated with the flv$^{1-•}$/flv^{2-} process, which is reproducible across repeated scans.

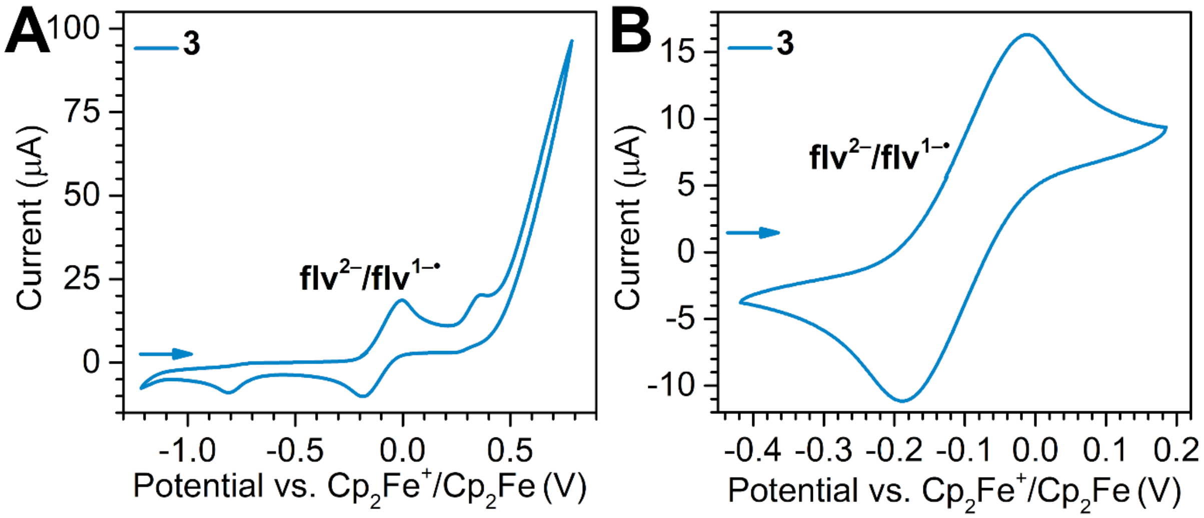

Figure S29. (**A**) Full cyclic voltammogram of [(Cp*$_2$Y)$_2$(μ-flv)] (**3**), measured in 1,2-difluorobenzene. (**B**) The magnification of the reversible redox feature is also part of main text Figure 4.

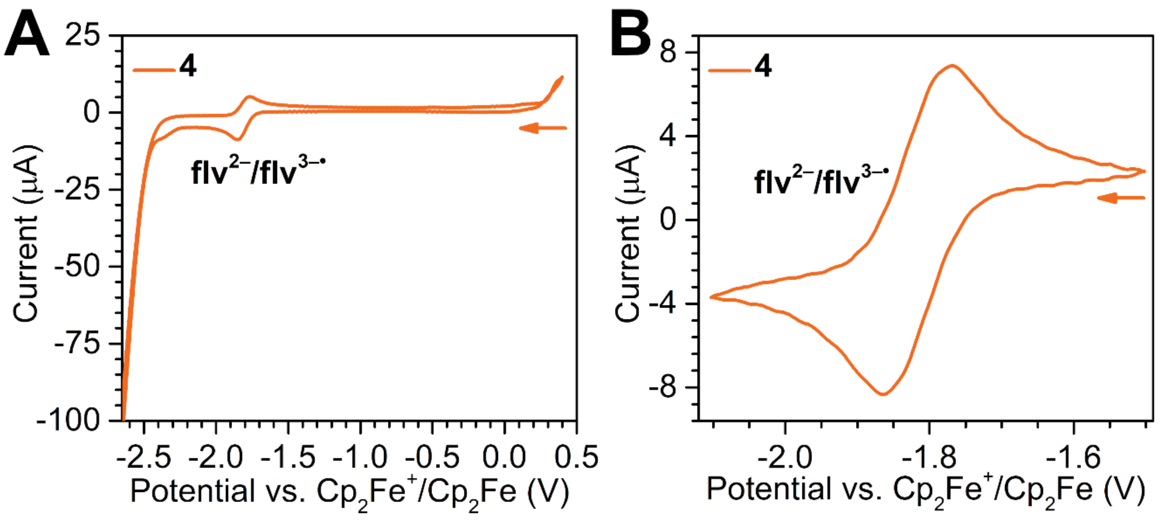

Figure S30. (**A**) Full cyclic voltammogram of [K(crypt-222)][(Cp*$_2$Y)$_2$(μ-flv•)] (**4**), measured in THF. (**B**) Magnification of the reversible redox feature is also depicted in Figure 4 in the main text.

Table S4. Summary of the cyclic voltammetry measurements on compounds **1-4** and the free ligand flv^0 versus the Fc^+/Fc^0 couple.

**Compound**	***E*$_{1/2}$(1) (V)** flv^0/flv$^{1-\bullet}$	***E*$_{1/2}$(2) (V)** flv$^{1-\bullet}$/flv^{2-}	***E*$_{1/2}$(3) (V)** flv^{2-}/flv$^{3-\bullet}$
**1** (THF)*	−0.999(3)	−1.756(3)	-
**1** (DFB)*	−0.902(4)	−1.608(3)	-
**1** (MeCN)*	−0.782(1)	−1.418(1)	-
**2** (DFB)^{O}	-	−0.935(2)	-
**3** (DFB)^{O}	-	−0.095(7)	-
**4** (THF)^{O}	-	-	−1.817(6)
flv^0 (THF)*	−0.96(1)	−1.73(1)	-
flv^0 (MeCN)^{R}	−0.80	−1.34	-

*: Referenced internally against ferrocene. ^{O}: Referenced externally against ferrocene under the same conditions as the analyte measurements. R: Literature values found in refence ([4])

1.8 Magnetic Data

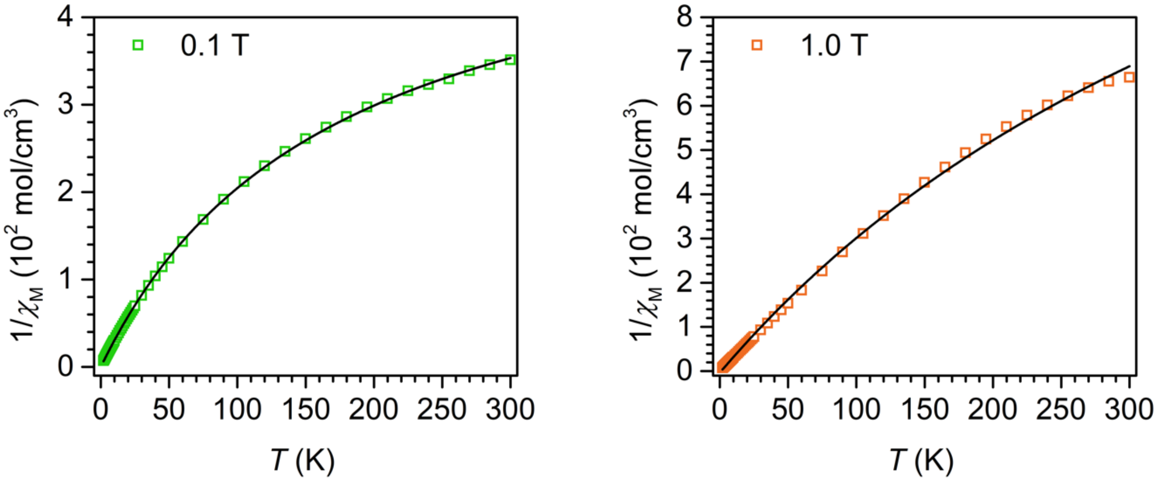

Figure S31. Curie-Weiss plots ($1/\chi_M$ vs. T) for $[(Cp^*_2Y)_2(\mu\text{-flv}^\bullet)][Al(OC\{CF_3\}_3)_4]$ (**2**) under 0.1 T (left) and 1.0 T (right) applied dc fields. The solid black lines are fits to an extended Curie-Weiss law $\frac{1}{\chi_M} = \frac{T-\theta_{CW}}{\chi_0\cdot(T-\theta_{CW})+C}$, where θ_{CW} is the Curie-Weiss temperature, C is the Curie constant, and χ_0 is a temperature-independent contribution to the susceptibility.[43]

Table S5. Parameters used to fit Curie-Weiss plots of $[(Cp^*_2Y)_2(\mu\text{-flv}^\bullet)][Al(OC\{CF_3\}_3)_4]$ (**2**).

H **(T)**	***Θ*** **(K)**	**χ_0 (10^{-3} cm^3 mol^{-1})**	***C*** **(cm^3 K mol^{-1})**
0.1	0.01(5)	1.8(1)	0.309(1)
1.0	0.9(3)	0.52(2)	0.277(4)

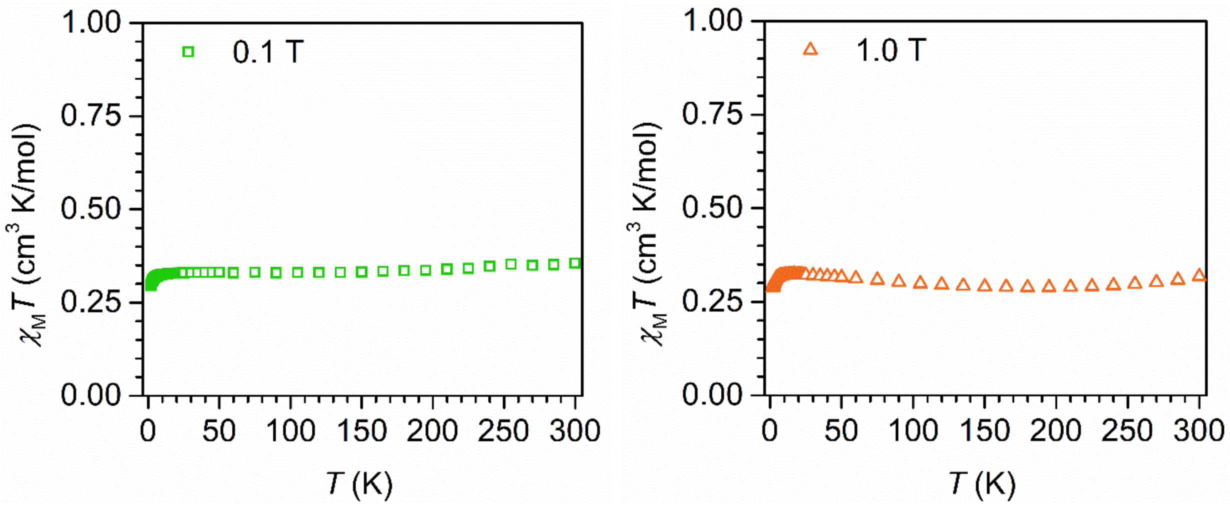

Figure S32. Variable-temperature magnetic susceptibility data for a polycrystalline restrained sample of [(Cp*$_2$Y)$_2$(μ-flv•)][Al(OC{CF_3}$_3$)$_4$] (**2**) under 0.1 T (left) and 1.0 T (right) applied dc fields, corrected for the diamagnetic moment of a blank sample and a small ferromagnetic impurity as determined via Curie-Weiss plots.

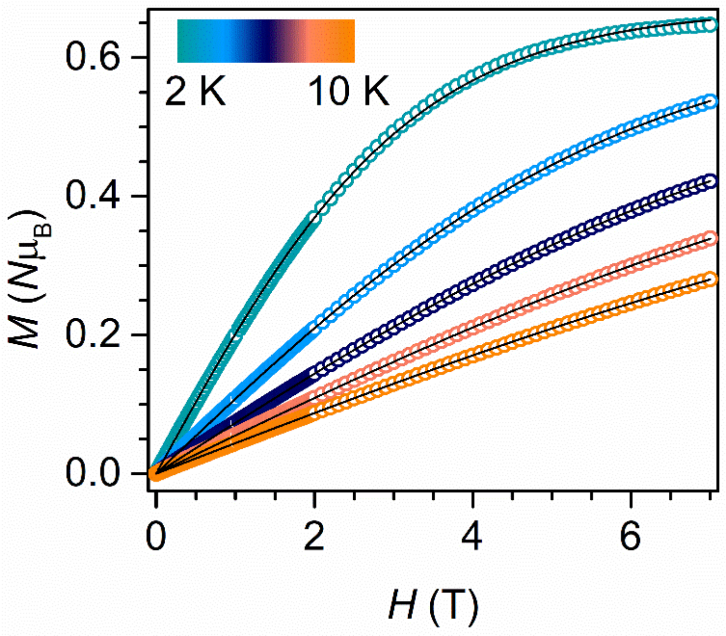

Figure S33. Variable-temperature variable-field magnetization data for [(Cp*$_2$Y)$_2$(μ-flv•)][Al(OC{CF_3}$_3$)$_4$] (**2**), recorded at 2 K, 4 K, 6 K, 8 K, and 10 K. Solid black lines represent fits to Brillouin functions for an S = ½ spin system.

Table S6. Parameters used to fit variable-temperature variable-field magnetization data of [(Cp*$_2$Y)$_2$(μ-flv•)][Al(OC{CF_3}$_3$)$_4$] (**2**) to a Brillouin function for a S = ½ spin system with a fixed g value to 2.0023.

***T* (K)**	**2 K**	**4 K**	**6 K**	**8 K**	**10 K**
N	0.647(1)	0.6478(1)	0.6474(3)	0.6460(4)	0.6443(4)

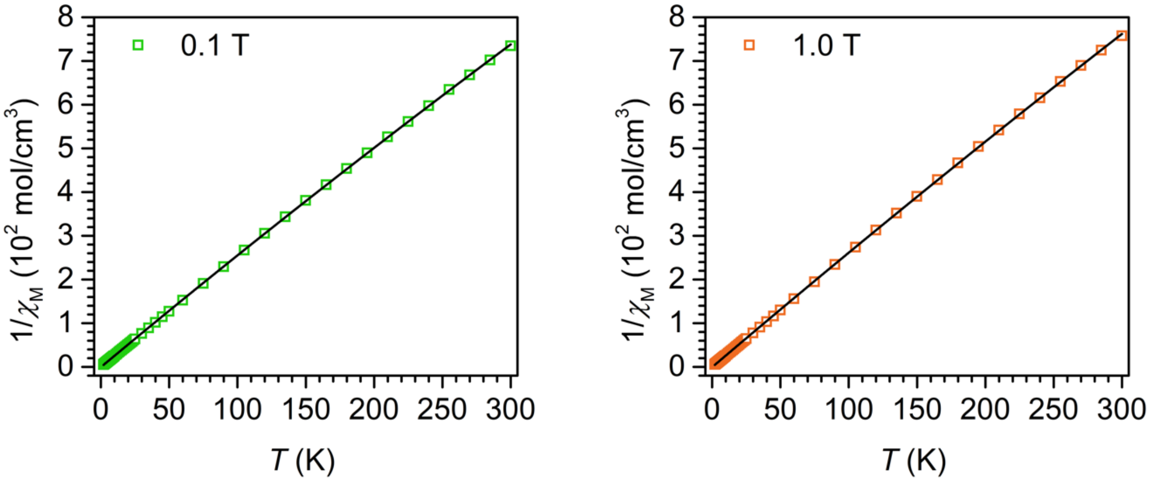

Figure S34. Curie-Weiss plots ($1/\chi_M$ vs. T) for [K(crypt-222)][(Cp*$_2$Y)$_2$(μ-flv•)] (**4**) under 0.1 T (left) and 1.0 T (right) applied dc fields. The solid black lines are fits to an extended Curie-Weiss law that accounts for a small paramagnetic impurity.

Table S7. Parameters used to fit Curie-Weiss plots of [K(crypt-222)][(Cp*$_2$Y)$_2$(μ-flv•)] (**4**).

H **(T)**	***Θ*** **(K)**	χ_0 **(10^{-5} cm^3 mol^{-1})**	***C*** **(cm^3 K mol^{-1})**
0.1	0.25(4)	7.6(3)	0.3835(7)
1.0	0.28(5)	5.8(6)	0.3760(8)

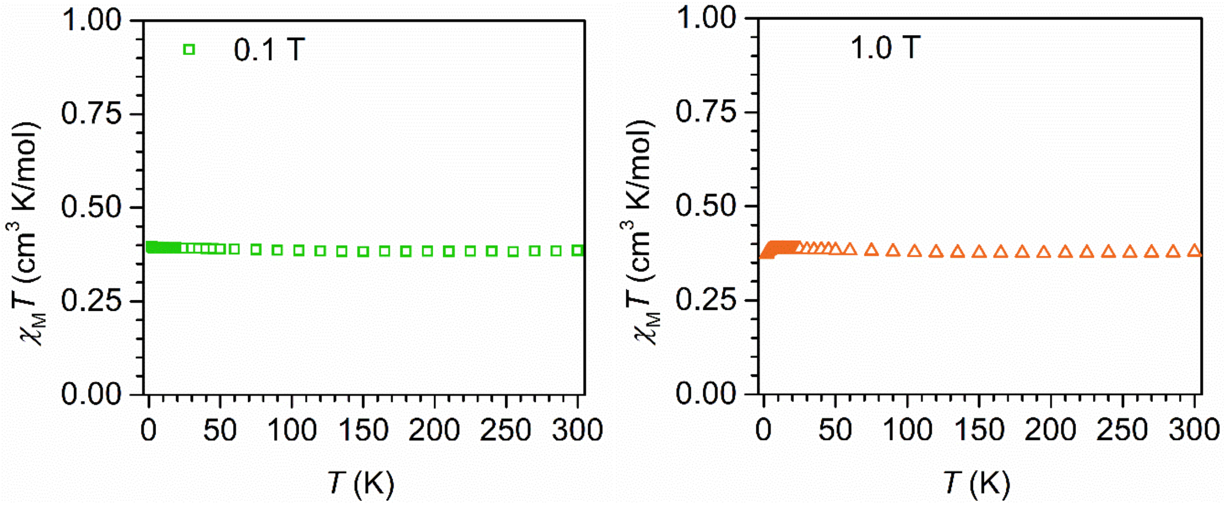

Figure S35. Variable-temperature magnetic susceptibility data for a polycrystalline restrained sample of [K(crypt-222)][(Cp*$_2$Y)$_2$(μ-flv•)] (**4**) under 0.1 T (left) and 1.0 T (right) applied dc fields, corrected for the diamagnetic moment of a blank sample and a small ferromagnetic impurity as determined via Curie-Weiss plots.

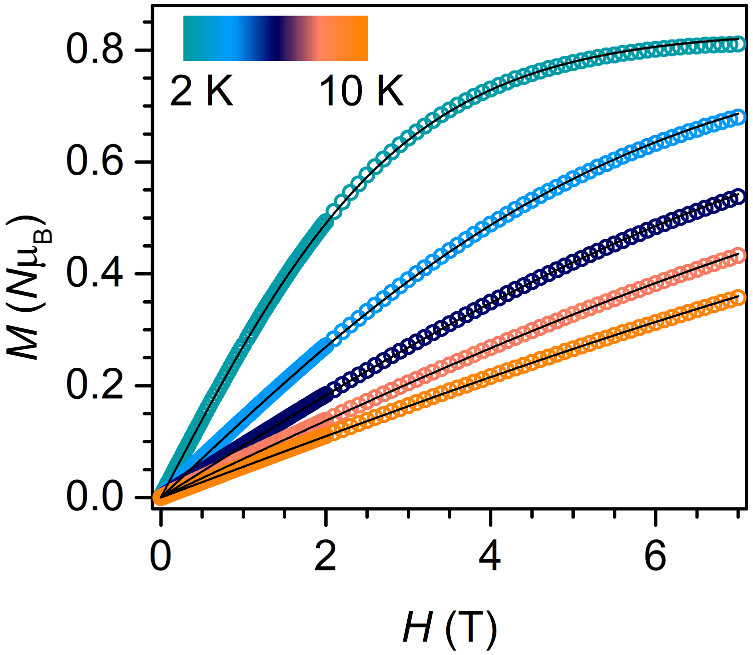

Figure S36. Variable-temperature variable-field magnetization data for [K(crypt-222)][(Cp^*_2Y)$_2$(μ-flv•)] (**4**), recorded at 2 K, 4 K, 6 K, 8 K, and 10 K. Solid black lines represent fits to Brillouin functions for an S = ½ spin system.

Table S8. Parameters used to fit variable-temperature, variable-field magnetization data of [K(crypt-222)][(Cp^*_2Y)$_2$(μ-flv•)] (**4**) to a Brillouin function for an S = ½ spin system with a fixed g value to 2.0023.

***T* (K)**	**2 K**	**4 K**	**6 K**	**8 K**	**10 K**
N	0.8339(4)	0.8293(4)	0.8262(4)	0.8227(3)	0.8187(3)

1.9 DFT Calculations

1.9.1 Structure Optimizations

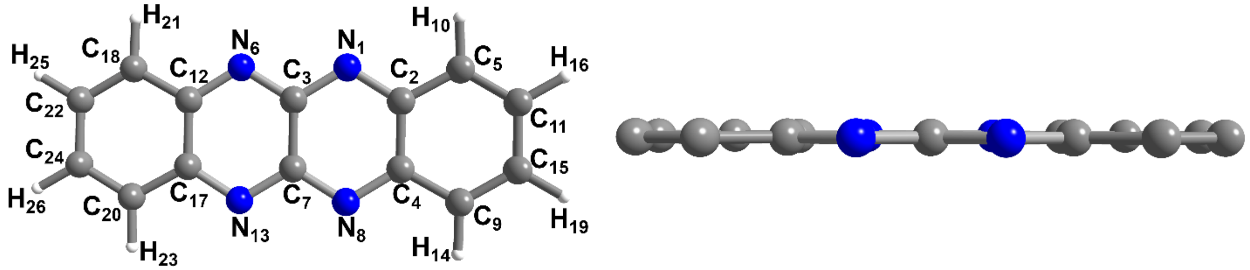

Figure S37. Optimized structure of the flv$^{1-\bullet}$ anion in [K(crypt-222)](flv•) (**1**), with atom labels for all N and C atoms as used below (left). Side view of the optimized structure to highlight the planarity of the flv$^{1-\bullet}$ anion, with the H atoms omitted for clarity (right).

Table S9. Structural parameters of the optimized geometries of the flv$^{1-\bullet}$ anion in [K(crypt-222)](flv•) (**1**) in comparison to the unoptimized values of **1** from single-crystal XRD. Negative signs represent elongation and positive values shortening of the calculated versus the experimental values.

Atoms	Calculated Distance (Å)	Experimental Distance (Å)	Difference
N6–C3	1.3428	1.342(2)	0.000
C3–N1	1.3428	1.348(3)	-0.005
**Avg. C3–N**	**1.3428**	**1.345(3)**	**-0.0022**
C7–N13	1.3428	1.348(3)	-0.005
C7–N8	1.3428	1.343(2)	0.000
**Avg. C17–N**	**1.3428**	**1.345(3)**	**-0.0024**
C12–N6	1.3504	1.357(3)	-0.007
C2–N1	1.3504	1.358(3)	-0.008
C4–N8	1.3504	1.358(3)	-0.008
C17–N13	1.3504	1.357(3)	-0.007
**Avg. outer C–N**	**1.3504**	**1.358(3)**	**-0.0073**
C17–C20	1.4161	1.415(3)	0.002
C20–C24	1.3785	1.371(3)	0.008
C24–C22	1.4105	1.409(3)	0.002
C22–C18	1.3786	1.373(3)	0.006
C18–C12	1.4161	1.415(3)	0.001
**C12–C17**	**1.4380**	**1.431(2)**	**0.007**
C2–C5	1.4161	1.414(3)	0.002
C5–C11	1.3785	1.371(3)	0.008
C11–C15	1.4105	1.409(3)	0.002
C15–C9	1.3786	1.373(3)	0.006
C9–C4	1.4161	1.415(3)	0.001
**C4–C2**	**1.4380**	**1.431(2)**	**0.007**
**Avg. C–C**	**1.406**	**1.402(3)**	**0.004**
**C3–C7**	**1.4698**	**1.468(3)**	**0.002**

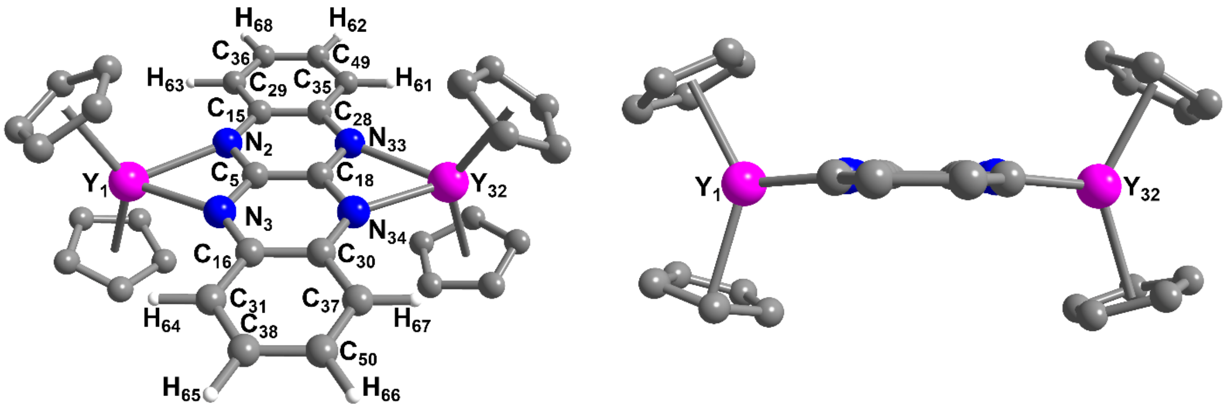

Figure S38. Optimized structure of [(Cp$_2$Y)$_2$(μ-flv•)]$^+$ (**2'**) with atom labels for all Y, N, and C atoms of the flv ligand as used below (left). The H atoms of the Cp ligands are omitted for clarity. Side view of the optimized structure to highlight the planarity of the flv$^{1-•}$ anion, with the H atoms omitted for clarity (right).

Table S10. Structural parameters of the optimized geometries of **2'** in comparison to the unoptimized values of [(Cp*$_2$Y)$_2$(μ-flv•)][Al(OC{CF$_3$}$_3$)$_4$], (**2**) from single-crystal XRD. Negative signs represent elongation and positive values shortening of the calculated versus the experimental values.

Atoms	Calculated Distance (Å)	Experimental Distance (Å)	Difference
Y1–N2	2.4286	2.439(3)	-0.010
Y1–N3	2.4304	2.431(3)	-0.001
Y32–N33	2.4354	2.431(3)	0.004
Y32–N34	2.4268	2.439(3)	-0.012
**Avg. Y–N**	**2.4303**	**2.435(3)**	**-0.005**
N2–C5	1.3395	1.349(5)	-0.010
C5–N3	1.3400	1.345(5)	-0.005
**Avg. C5–N**	**1.3398**	**1.3470(5)**	**-0.007**
C18–N33	1.3398	1.345(5)	-0.005
C18–N34	1.3399	1.349(5)	-0.009
**Avg. C18–N**	**1.3399**	**1.3470(5)**	**-0.007**
C15−N2	1.3614	1.366(5)	-0.005
C16−N3	1.3617	1.379(5)	-0.017
C28−N33	1.3616	1.379(5)	-0.017
C30−N34	1.3614	1.366(5)	-0.005
**Avg. outer C–N**	**1.3507**	**1.360(5)**	-0.009
C15–C29	1.4073	1.417(5)	-0.010
C29–C36	1.3755	1.368(6)	0.007
**C36–C49**	**1.4110**	**1.403(6)**	**0.008**
C49–C35	1.3755	1.372(6)	0.003
C35–C28	1.4073	1.409(5)	-0.002
**C28–C15**	**1.4359**	**1.424(6)**	**0.012**

C30–C37	1.4072	1.417(5)	-0.010
C37–C50	1.3754	1.368(6)	0.007
**C50–C38**	**1.4110**	**1.403(6)**	**0.008**
C38–C31	1.3755	1.372(6)	0.003
C31–C16	1.4073	1.409(5)	-0.002
**C16–C30**	**1.4358**	**1.424(6)**	**0.012**
**Avg. C–C**	**1.402**	**1.399(6)**	**0.003**
**C5–C18**	**1.4284**	**1.429(7)**	**-0.001**
**Y–Y**	**7.0977**	**7.114(6)**	**-0.016**

Table S11. Selected angles of the optimized geometries of **2'** in comparison to the unoptimized values of $[(Cp^*{}_2Y)_2(\mu\text{-flv}^\bullet)][Al(OC\{CF_3\}_3)_4]$, (**2**) from single-crystal XRD. Negative signs indicate enlargement of the calculated angles compared to the experimental angles, whereas positive signs indicate contraction.

**Experimental Values**		**Calculated**	
**Atoms**	**Angle (°)**	**Value (°)**	**Difference**
**Angles vs Ligand Plane (C38, C50, C36, C49)**			
Y1–N2–N3 Plane	4.931	3.1850	1.746
Y32–N33–N34 Plane	4.388	3.1850	1.203
**Bending Angle Metallocene Moiety**			
Cnt–Y1–Cnt	136.101	141.8270	-5.726
Cnt–Y32–Cnt	135.952	141.8270	-5.875

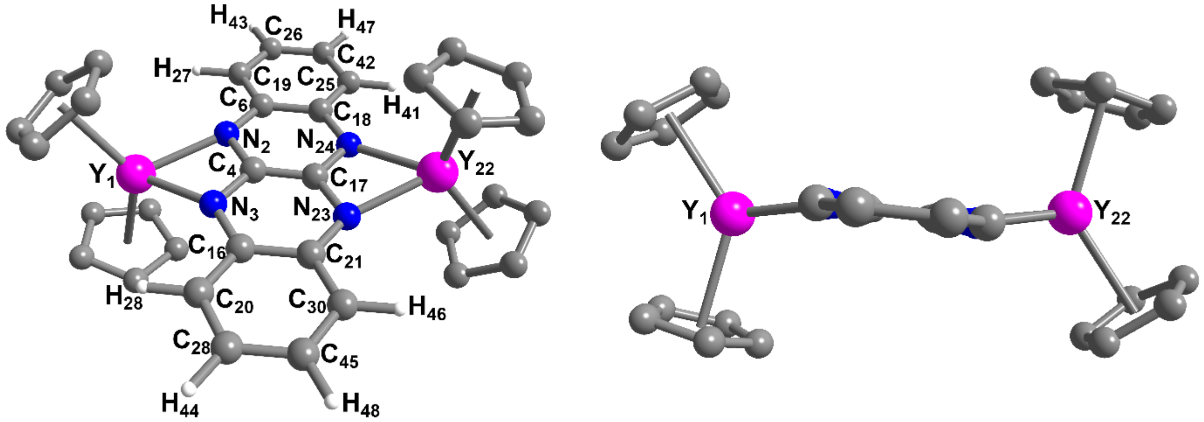

Figure S39. Optimized structure of $[(Cp_2Y)_2(\mu\text{-flv}^\bullet)]^-$ (**4'**) with atom labels for all Y, N, and C atoms of the flv ligand as used below (left). The H atoms of the Cp ligands are omitted for clarity. Side view of the optimized structure to highlight the planarity of the $flv^{3-\bullet}$ anion, with the H atoms omitted for clarity (right).

Table S12. Structural parameters of the optimized geometries of **4'** in comparison to the unoptimized values of [K(crypt-222)][($Cp^*_2Y)_2$(μ-flv•)] (**4**), from single-crystal XRD. Negative signs represent elongation and positive values shortening of the calculated versus the experimental values.

Atoms	Calculated Distance (Å)	Experimental Distance (Å)	Difference
Y1–N2	2.2880	2.316(3)	−0.028
Y1–N3	2.2900	2.322(3)	−0.032
Y22–N23	2.2900	2.316(3)	−0.026
Y22–N24	2.2880	2.322(3)	−0.034
**Avg. Y–N**	**2.2890**	**2.319(3)**	**−0.030**
N2–C4	1.3770	1.380(5)	−0.003
C4–N3	1.3810	1.383(5)	−0.013
**Avg. C4–N**	**1.3780**	**1.391(5)**	**−0.005**
C17–N23	1.3770	1.380(5)	−0.003
C17–N23	1.3780	1.391(5)	−0.013
**Avg. C17–N**	**1.3795**	**1.385(5)**	**−0.005**
C6–N2	1.3810	1.383(5)	−0.002
C16–N3	1.3820	1.385(5)	−0.003
C18–N24	1.3820	1.385(5)	−0.003
C21–N23	1.3810	1.383(5)	−0.002
**Avg. outer C–N**	**1.3795**	**1.385(5)**	−0.005
C6–C19	1.3980	1.395(5)	0.003
C19–C26	1.3970	1.391(5)	0.006
C26–C42	1.3890	1.379(6)	0.010
C42–C25	1.3960	1.388(5)	0.008
C25–C18	1.3980	1.396(5)	0.002
C18–C6	1.4310	1.434(5)	−0.003
C16–C20	1.3980	1.396(5)	0.002
C20–C29	1.3960	1.388(5)	0.008
C29–C45	1.3890	1.379(6)	0.010
C45–C30	1.3970	1.391(5)	0.006
C30–C21	1.3980	1.395(5)	0.003
C16–C21	1.4310	1.434(5)	−0.003
**Avg. C–C**	**1.402**	**1.397(5)**	**0.004**
**C4–C17**	**1.3810**	**1.373(7)**	**0.008**
**Y–Y**	**6.7550**	**6.877(9)**	**−0.122**

Table S13. Selected angles of the optimized geometries of [($Cp_2Y)_2$(μ-flv•)]$^-$ (**4'**) in comparison to the unoptimized values of [K(crypt-222)][($Cp^*_2Y)_2$(μ-flv•)] (**4**) from single-crystal XRD. Negative signs indicate enlargement of the calculated angles compared to the experimental angles, whereas positive signs indicate contraction.

Experimental Values		Calculated	
**Atoms**	**Angle (°)**	**Value (°)**	**Difference**
**Angles vs Ligand Plane (C26, C42, C29, C45)**			
Y1–N2–N3 Plane	12.3	8.8	3.5
Y22–N23–N24 plane	12.3	8.8	3.5
**Bending Angle Metallocene Moiety**			
Cnt–Y1–Cnt	131.1	137.3	−6.2
Cnt–Y22–Cnt	131.1	137.3	−6.2

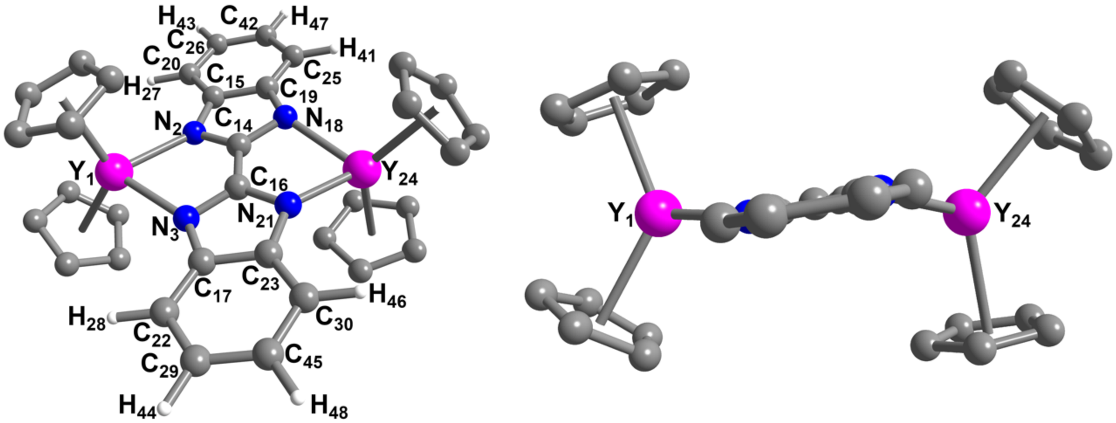

Figure S40. Optimized structure of [($Cp_2Y)_2$(μ-Bbim•)]$^-$ with atom labels for all Y, N and C atoms of the flv ligand as used below (left) . The H atoms of the Cp ligands are omitted for clarity. Side view of the optimized structure to highlight the planarity of the $Bbim^{3-•}$ anion, with the H atoms omitted for clarity (right).

Table S14. Structural parameters of the optimized geometries of [($Cp_2Y)_2$(μ-Bbim•)]$^-$ in comparison to the unoptimized values of [K(crypt-222)][($Cp^*_2Y)_2$(μ-Bbim•)] from single-crystal XRD.[34] Negative signs represent elongation and positive values shortening of the calculated versus the experimental values.

Atoms	Calculated Distance (Å)	Experimental Distance (Å)	Difference
Y1–N2	2.318	2.355(2)	−0.037
Y1–N3	2.324	2.364(2)	−0.040
Y24–N18	2.322	2.374(2)	−0.052
Y24–N21	2.324	2.359(2)	−0.035
**Avg. Y–N**	**2.322**	**2.363(2)**	**−0.041**
C14–N2	1.379	1.384(2)	−0.005
C16–N3	1.380	1.384(3)	−0.004

C14–N18	1.379	1.381(3)	−0.002
C16–N21	1.379	1.381(2)	−0,.002
**Avg. central C–N**	**1.379**	**1.383(3)**	**−0.003**
N2–C15	1.375	1.377(2)	−0.002
N3–C17	1.376	1.382(2)	−0.006
N18–C19	1.376	1.385(2)	−0.009
N21–C23	1.376	1.380(2)	−0.004
**Avg. outer C–N**	**1.375**	**1.381(2)**	**−0.006**
C15–C20	1.394	1.401(3)	−0.007
C20–C26	1.400	1.399(3)	0.001
C26–C42	1.396	1.389(3)	0.007
C42–C25	1.400	1.402(3)	−0.002
C25–C19	1.394	1.394(3)	0.000
C17–C22	1.394	1.391(3)	0.003
C22–C29	1.400	1.401(3)	−0.001
C29–C45	1.396	1.392(3)	0.004
C45–C30	1.400	1.401(3)	−0.001
C30–C23	1.394	1.394(3)	0.000
**Avg. C–C**	**1.397**	**1.396(3)**	**0.000**
C15–C19	1.428	1.428(3)	0.000
C17–C23	1.428	1.428(3)	0.000
**Avg. Imidazole C–C**	**1.428**	**1.428(3)**	**0.000**
**C14–C16**	**1.403**	**1.402(3)**	**0.001**
**Y–Y**	**5.843**	**6.030(5)**	**−0.187**

Table S15. Selected angles of the optimized geometries of [($Cp_2Y)_2$(μ-Bbim•)] in comparison to the unoptimized values of [K(crypt-222)][($Cp^*_2Y)_2$(μ-Bbim•)] from single-crystal XRD.[34] Negative signs indicate enlargement of the calculated angles compared to the experimental angles, whereas positive signs indicate contraction.

Experimental Values		Calculated	
**Atoms**	**Angle (°)**	**Value (°)**	**Difference**
**Angles vs Ligand Plane (C26,C42,C29,C45)**			
Y1–N2–N3 Plane	12.046	7.934	4.112
Y22–N23–N24 plane	22.339	13.044	9.295
**Bending Angle Metallocene Moiety**			
Cnt–Y1–Cnt	130.441	135.35(2)	−4.909
Cnt–Y22–Cnt	130.728	136.17(2)	−5.442

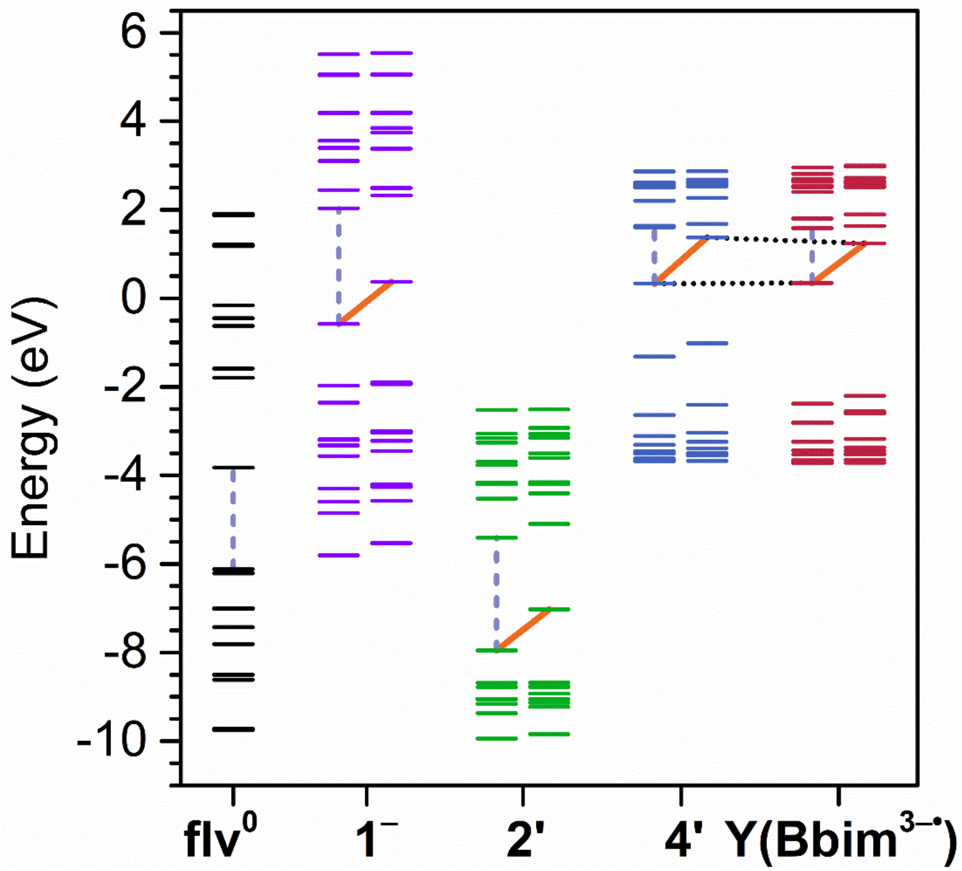

Figure S41. Frontier orbital energies for all calculated compounds in eV, where α-spin manifolds are depicted left and β-spin manifolds right for each compound. Orange diagonal lines represent differences between singly occupied α-spin molecular orbital (SOMO) and associated SOMO* of the β-spin manifold, which can be used as an estimation for the spin polarization within a given system. Energy difference in each system: 0.95 eV **1**$^{-}$, 0.86 eV **2'**, 1.05 eV **4'**, 0.89 eV Y(Bbim$^{3-•}$). Spin polarization difference of Y(flv$^{1-•}$) vs. Y(flv$^{3-•}$): +17.4%. Spin polarization difference of Y(flv$^{3-•}$) vs. Y(Bbim$^{3-•}$): −17.2%. Gray lines represent the gap between highest occupied (HOMO) and lowest unoccupied (LUMO) of the α-spin manifolds. Dotted lines connect the β SOMO* in isomeric [(Cp$_2$Y)$_2$(μ-flv•)]$^{-}$ and [(Cp$_2$Y)$_2$(μ-Bbim•)]$^{-}$ complexes to emphasize changes in spin polarization due to geometry changes. HOMO-LUMO gap α manifold: 2.61 eV **1**$^{-}$, 2.18 eV **2'**, 1.28 eV **4'**, 1.24 eV Y(Bbim$^{3-•}$). Energy difference of corresponding β orbitals: 1.95 eV **1**$^{-}$, 1.65 eV **2'**, 0.30 eV **4'**, 0.39 eV Y(Bbim$^{3-•}$).

1.9.2 Natural Localized Molecular Orbital Analysis

Table S16. Results of the hybridization/polarization analysis of [(Cp$_2$Y)$_2$(μ-flv•)]$^{+}$ (**2'**) NLMOs, given as the % contributions of parent NBOs from uTPSSh calculations for α manifold.

NLMO No.	Description	NLMO contributions α manifold			Hybrid Character Y			Hybrid Character N		
		Y1	Y32	N	s	p	d	s	p	d
47	LP1 N2	5.0	-	91.7	28.8	-	70.9	30.6	69.4	-
48	LP1 N3	5.0	-	91.7	33.6	.	66.1	30.4	69.6	-
53	LP1 N33	-	5.0	91.7	31.6	-	68.1	30.4	69.6	-

54	LP1 N34	-	5.0	91.8	30.0	-	69.7	30.6	69.4	-

Table S17. Results of the hybridization/polarization analysis of $[(Cp_2Y)_2(\mu\text{-flv}\bullet)]^+$ (**2'**) NLMOs, given as the % contributions of parent NBOs from uTPSSh calculations for *β* manifold.

NLMO No.	Description	**NLMO contributions *β* manifold**			**Hybrid Character Y**			**Hybrid Character N**		
		Y1	Y32	N	s	p	d	s	p	d
47	LP1 N2	5.1	-	91.6	28.2	-	71.5	30.2	69.8	-
48	LP1 N3	5.1	-	91.5	33.0	-	66.7	29.9	70.0	-
52	LP1 N33	-	5.1	91.6	31.5	-	68.2	29.9	70.0	-
53	LP1 N34	-	5.1	91.6	29.8	-	69.9	30.2	69.8	-

Table S18. Results of the second order perturbation analysis for $[(Cp_2Y)_2(\mu\text{-flv}\bullet)]^+$ (**2'**). Only strong interactions >3 kcal/mol are listed, excluding interactions involving the Cp rings.

Donor NBO	Acceptor NBO	***E* (kcal/mol) *α***	Donor NBO	Acceptor NBO	***E* (kcal/mol) *β***
LP1 N2	LV2 Y1	8.32	LP1 N2	LV2 Y1	8.71
LP1 N2	LV4 Y1	10.92	LP1 N2	LV4 Y1	10.90
LP1 N2	LV5 Y1	4.96	LP1 N2	LV5 Y1	5.03
LP1 N3	LV2 Y1	9.30	LP1 N3	LV2 Y1	9.72
LP1 N3	LV4 Y1	7.98	LP1 N3	LV4 Y1	8.31
LP1 N3	LV5 Y1	5.96	LP1 N3	LV5 Y1	5.86
BD1 N3−C5	LV2 Y1	3.00	BD1 N3−C5	LV2 Y1	3.01
LP1 N2	BD*1 C5−C18	5.32	LP1 N2	BD*1 C5−C18	5.38
LP1 N2	BD*1 C15−C28	4.29	LP1 N2	BD*1 C15−C28	4.28
LP1 N3	BD*1 C5−C18	5.32	LP1 N3	BD*1 C5−C18	5.38
LP1 N3	BD*1 C16−C30	4.28	LP1 N3	BD*1 C16−C30	4.27
LP1 C15	BD*2 N2−C5	36.16	LP1 C18	BD*2 C28−N33	37.48
LP1 C15	BD*1 C28−N33	84.87	LP1 C18	BD*2 C30−N34	37.52
LP1 C15	BD*1 C29−C36	36.12	LP1 N33	BD*1 C5−C18	5.39
LP1 C30	BD*2 N3−C16	85.12	LP1 N33	BD*1 C15−C28	4.30
LP1 C30	BD*2 C18−N34	36.12	LP1 N34	BD*1 C5−C18	5.37
LP1 C30	BD*2 C37−C50	36.51	LP1 N34	BD*1 C16−C30	4.28
LP1 N33	BD*1 C5−C18	5.33	BD1 N2−C15	LV1 C5	43.58
LP1 N33	BD*1 C18−N34	4.31	BD2 N2−C15	BD*2 C28−C33	6.65
LP1 N34	BD*1 C5−C18	5.31	BD2 N2−C15	BD*2 C29−C36	5.15

LP1 N34	BD*1 C16−C30	4.29	BD2 N3−C16	LV1 C5	43.53
BD2 N2−C5	BD*2 C18−N34	5.36	BD2 N3−C16	BD*2 C30−N34	6.66
BD2 N3−C16	BD*2 N2−C5	21.90	BD2 N3−C16	BD*2 C31−C38	5.19
BD2 N3−C16	BD*2 C31−C38	3.56	BD2 C28−N33	BD*2 N2−C15	6.67
BD2 C18−N34	BD*2 N2−C5	5.34	BD2 C28−N33	BD*2 C35−C49	5.21
BD2 C28−N33	BD*2 C18−N34	21.92	BD2 C29−C36	BD*2 N2−C15	14.50
BD2 C28−N33	BD*2 C35−C49	3.58	BD2 C29−C36	BD*2 C35−C49	7.77
BD2 C29−C36	BD*2 C35−C49	8.41	BD2 C30−N34	BD*2 N3−C16	6.67
BD2 C31−C38	BD*2 N3−C16	17.20	BD2 C30−N34	BD*2 C37−C50	5.20
BD2 C31−C38	BD*2 C37−C50	8.23	BD2 C31−C38	BD*2 N3−C16	14.43
BD2 C35−C49	BD*2 C28−N33	17.00	BD2 C31−C38	BD*2 C37−C50	7.60
BD2 C35−C49	BD*2 C29−C36	8.49	BD2 C35−C49	BD*2 C28−N33	14.24
LP1 N33	LV2 Y32	9.47	BD2 C35−C49	BD*2 C29−C36	7.85
LP1 N33	LV4 Y32	9.40	BD2 C37−C50	BD*2 C30−N34	14.43
LP1 N33	LV5 Y32	4.83	BD2 C37−C50	BD*2 C31−C38	7.60
LP1 N34	LV2 Y32	7.70	LP1 N33	LV2 Y32	9.86
LP1 N34	LV4 Y32	9.15	LP1 N33	LV4 Y32	9.25
LP1 N34	LV5 Y32	5.82	LP1 N33	LV5 Y32	4.92
			LP1 N34	LV2 Y32	8.03
			LP1 N34	LV4 Y32	9.66
			LP1 N34	LV5 Y32	5.69

Table S19. Results of the hybridization/polarization analysis of $[(Cp_2Y)_2(\mu\text{-flv}\bullet)]^-$ (**4'**) NLMOs, given as the % contributions of parent NBOs from uTPSSh calculations for α manifold.

NLMO No.	Description	**NLMO contributions α manifold**			**Hybrid Character Y**			**Hybrid Character N**		
		Y1	Y22	N	s	p	d	s	p	d
47	LP1 N2	6.3	-	90.7	27.9	-	71.8	32.9	67.0	-
48	LP2 N2	2.0	-	80.7	1.7	0.9	96.0	-	99.9	0.1
49	LP1 N3	6.4	-	90.6	32.9	0.2	66.8	32.8	67.2	1.7
50	LP2 N3	1.9	-	81.4	1.5	0.9	96.2	-	99.9	0.1
53	LP1 N23	-	6.4	90.7	27.9	0.2	71.8	32.9	67.0	-
54	LP2 N23	-	2.0	80.7	1.4	0.9	96.3	-	99.9	0.1
55	LP1 N24		6.4	90.6	33.1	0.2	66.6	32.8	67.2	-
56	LP2 N24	-	1.9	81.4	1.3	0.7	96.5	-	99.9	0.1

Table S20. Results of the hybridization/polarization analysis of [(Cp$_2$Y)$_2$(μ-flv•)]$^-$ (**4'**) NLMOs, given as the % contributions of parent NBOs from uTPSSh calculations for *β* manifold.

NLMO No.	Description	NLMO contributions *β* manifold			Hybrid Character Y			Hybrid Character N		
		Y1	Y22	N	s	p	d	s	p	d
47	LP1 N2	6.5	-	90.6	27.5	0.2	72.2	32.3	67.6	-
48	LP1 N3	6.5	-	90.5	32.4	0.2	67.3	32.1	94.9	-
53	LP1 N23	-	6.5	90.5	27.5	0.2	72.2	32.3	67.6	-
54	LP1 N24	-	6.5	90.5	32.4	0.2	67.3	32.1	67.9	-

Table S21. Results of the second order perturbation analysis for [(Cp$_2$Y)$_2$(μ-flv•)]$^-$ (**4'**). Only strong interactions > 3 kcal/mol are listed, excluding interactions involving the Cp rings.

Donor NBO	Acceptor NBO	*E* (kcal/mol) α	Donor NBO	Acceptor NBO	*E* (kcal/mol) β
LP1 N2	LV1 Y1	12.74	LP1 N2	LV1 Y1	13.64
LP1 N2	LV3 Y1	10.46	LP1 N2	LV2 Y1	10.87
LP1 N2	LV5 Y1	5.27	LP1 N2	LV5 Y1	5.28
LP1 N3	LV1 Y1	13.37	LP1 N3	LV1 Y1	14.32
LP1 N3	LV2 Y1	9.87	LP1 N3	LV2 Y1	10.31
LP1 N3	LV5 Y1	5.46	LP1 N3	LV5 Y1	5.45
LP1 N2	BD*1 C4–C17	4.35	BD1 N2–C4	LV1 Y1	3.01
LP1 N2	BD*1 C6–C19	3.95	LP1 N3–C4	LV1 Y1	3.08
LP2 N2	BD*2 C4–C17	24.22	LP1 N2	BD*1 N3–C4	4.27
LP2 N2	BD*2 C6–C19	24.30	LP1 N2	BD*1 C6–C18	4.00
LP1 N3	BD*2 C4–C17	4.35	LP1 N3	BD*1 C4–C17	4.26
LP1 N3	BD*2 C16–C21	3.95	LP1 N3	BD*1 C16–C21	4.00
LP2 N3	BD*2 C4–C17	24.18	LP1 C6	BD*1 N2–C4	28.04
LP2 N3	BD*2 C16–C20	24.28	LP1 C6	BD*1 C18–C24	80.90
LP1 N23	BD*1 C4–C17	4.35	LP1 C6	BD*1 C19–C26	46.70
LP1 N23	BD*1 C16–C21	3.95	LP1 C21	BD*2 N3–C16	80.88
LP2 N23	BD*2 C4–C17	24.21	LP1 C21	BD*2 C17–CN23	28.05
LP2 N23	BD*2 C21–C30	24.30	LP1 C21	BD*2 C30–C45	46.69
LP1 N24	BD*1 C4–C17	4.35	LP1 N23	BD*1 C4–C27	4.27
LP1 N24	BD*1 C6–C18	3.95	LP1 N23	BD*1 C16–C21	4.01
LP2 N24	BD*2 C4–C17	24.17	LP1 N24	BD*1 C4–C17	4.26
LP2 N24	BD*2 C18–C25	24.28	LP1 N24	BD*1 C6–C18	4.00
BD2 C2–C19	BD*2 C18–C25	7.92	BD2 N3–C16	BD*2 N2–C4	19.39
BD2 C2–C19	BD*2 C26–C42	10.01	BD1 N3–C16	BD*2 C20–C29	4.67
BD2 C16–C20	BD*2 C21–C30	7.92	BD2 C17–N23	BD*2 N2–C4	6.49
BD2 C16–C20	BD*2 C29–C45	10.00	BD2 C17–N23	BD*2 C18–N24	3.09
BD2 C18–C25	BD*2 C6–C19	7.92	BD2 C18–N24	BD*2 C17–N23	19.9
BD2 C18–C25	BD*2 C26–C42	10.00,	BD2 C18–N24	BD*2 C25–C42	4.67
BD2 C21–C30	BD*2 C16–C20	7.92	BD2 C20–C29	BD*2 N3–C16	15.41
BD2 C21–C30	BD*2 C29–C45	10.01	BD2 C20–C29	BD*2 C30–C45	8.85
LP1 N23	LV1 Y22	12.72	BD2 C25–C42	BD*2 C18–N24	15.40
LP1 N23	LV3 Y22	10.46	BD2 C25–C42	BD*2 C19–C26	8.85
LP1 N23	LV5 Y22	5.28	LP1 N23	LV1 Y22	13.63
LP1 N24	LV1 Y22	13.39	LP1 N23	LV2 Y22	10.89

LP1 N23	LV3 Y22	9.90	LP1 N24	LV1 Y22	14.33
LP1 N23	LV5 Y22	5.46	LP1 N24	LV2 Y22	10.32
BD1 C17–N24	LV1 Y22	3.00	LP1 N24	LV5 Y22	5.45
			BD1 C17–N23	LV1 Y22	3.01
			BD1 C17–N24	LV1 Y22	3.08

Table S22. Results of the hybridization/polarization analysis for NLMOs of $[(Cp_2Y)_2(\mu\text{-Bbim}\bullet)]^-$, a contracted model of $[K(crypt\text{-}222)][(Cp^*{}_2Y)_2(\mu\text{-Bbim}\bullet)]$, given as the % contributions of parent NBOs from uTPSSh calculations for α manifold.

NLMO No.	Description	**NLMO contributions *α* manifold** Y1	Y24	N	**Hybrid Character Y** s	p	d	**Hybrid Character N** s	p	d
47	LP1 N2	6.1	-	91.4	28.6	0.2	71.1	35.0	65.0	-
48	LP2 N2	1.4	-	78.4	1.1	0.6	96.8	-	99.9	-
49	LP1 N3	6.1	-	91.4	33.3	0.2	66.5	31.4	67.3	1.3
50	LP2 N3	1.6	-	78.6	1.1	0.7	96.9	-	99.9	-
53	LP1 N18	-	6.0	91.4	28.0	0.2	71.7	35.2	64.8	-
54	LP2 N18	-	1.8	77.3	4.0	0.5	94.4	-	99.9	-
55	LP1 N21		6.0	91.4	32.1	0.2	67.6	35.2	64.8	-
56	LP2 N21		2.1	77.5	4.2	0.6	94.3	-	99.9	-

Table S23. Results of the hybridization/polarization analysis for NLMOs of $[(Cp_2Y)_2(\mu\text{-Bbim}\bullet)]^-$, a contracted model of $[K(crypt\text{-}222)][(Cp^*{}_2Y)_2(\mu\text{-Bbim}\bullet)]$, given as the % contributions of parent NBOs from uTPSSh calculations for β manifold.

NLMO No.	Description	**NLMO contributions *β* manifold** Y1	Y24	N	**Hybrid Character Y** s	p	d	**Hybrid Character N** s	p	d
47	LP1 N2	6.3	-	91.2	28.5	0.2	71.3	34.5	65.5	-
48	LP1 N3	6.2	-	91.3	33.1	0.2	66.6	34.6	65.4	-
51	LP1 N18	-	6.2	91.2	27.8	0.2	71.9	34.8	65.1	-
53	LP1 N21	-	6.2	91.3	32.0	0.2	67.7	34.8	65.1	-

Table S24. Results of the second order perturbation analysis for $[(Cp_2Y)_2(\mu\text{-Bbim}\bullet)]$, a contracted model of $[K(crypt\text{-}222)][(Cp^*{}_2Y)_2(\mu\text{-Bbim}\bullet)]$. Only strong interactions >3 kcal/mol are listed, excluding interactions involving the Cp rings.

Donor NBO	Acceptor NBO	***E* (kcal/mol) *α***	Donor NBO	Acceptor NBO	***E* (kcal/mol) *β***
LP1 N2	LV1 Y1	11.77	LP1 N2	LV1 Y1	12.15
LP1 N2	LV2 Y1	13.11	LP1 N2	LV2 Y1	13.11
LP1 N2	LV5 Y1	6.72	LP1 N2	LV5 Y1	6.72
LP2 N2	LV3 Y1	3.77	LP1 N3	LV1 Y1	14.12
LP1 N3	LV1 Y1	13.98	LP1 N3	LV2 Y1	11.17
LP1 N3	LV2 Y1	10.82	LP1 N3	LV5 Y1	7.02
LP1 N3	LV5 Y1	7.03	LP1 N2	BD*1 C14–C16	4.10

LP1 N2	BD*1 C14–N18	4.10	LP1 N3	BD*1 C16–N21	4.10
LP2 N2	BD*2 C14–C16	24.04	LP1 N18	BD*1 N2–C14	4.14
LP2 N2	BD*2 C15–C20	27.36	LP1 C19	BD*2 N2–C15	70.51
LP1 N3	BD*1 C16–N21	4.10	LP1 C19	BD*2 C14–N18	27.53
LP2 N3	BD*2 C14–C16	23.95	LP1 C19	BD*2 C25–C42	46.09
LP2 N3	BD*2 C17–C22	27.29	LP1 N21	BD*1 N3–C16	4.15
LP1 N18	BD*1 N2–C14	4.14	LP1 C23	BD*2 N3–C17	70.74
LP2 N18	BD*2 C14–C16	24.34	LP1 C23	BD*2 C16–N21	27.37
LP2 N18	BD*2 C19–C25	27.24	LP1 C23	BD*2 C30–C45	46.13
LP1 N21	BD*1 N3–C16	4.16	BD2 N2–C15	BD*2 C14–N18	19.14
LP2 N21	BD*2 C14–C16	24.29	BD2 N2–C15	BD*2 C20–C26	4.79
LP2 N21	BD*2 C23–C30	27.23	BD2 N3–C17	BD*2 C16–N21	19.12
BD2 C15–C20	BD*2 C19–C25	7.52	BD2 N3–C17	BD*2 C22–C29	4.78
BD2 C15–C20	BD*2 C26–C42	10.38	BD2 C14–N18	BD*2 N2–C15	3.14
BD2 C17–C22	BD*2 C23–C30	7.54	BD2 C14–N18	BD*2 C16–N21	6.32
BD2 C17–C22	BD*2 C29–C45	10.37	BD2 C16–N21	BD*2 C14–N18	6.34
BD2 C19–C25	BD*2 C26–C42	10.46	BD2 C20–C26	BD*2 N2–C15	15.80
BD2 C23–C30	BD*2 C29–C45	10.47	BD2 C20–C26	BD*2 C25–C42	8.80
BD2 C26–C42	BD*2 C15–C20	6.71	BD2 C22–C29	BD*2 N3–C17	15.80
BD2 C26–C42	BD*2 C19–C25	6.68	BD2 C22–C29	BD*2 C30–C45	8.78
BD2 C29–C45	BD*2 C17–C22	6.73	BD2 C25–C42	BD*2 C20–C26	8.91
BD2 C 29–C 45	BD*2 C23–C30	6.69	LP1 N18	LV1 Y24	11.82
LP1 N 18	LV1 Y24	12.22	LP1 N18	LV2 Y24	12.59
LP1 N 18	LV2 Y24	12.17	LP1 N18	LV5 Y24	6.71
LP1 N 18	LV5 Y24	6.25	LP1 N21	LV1 Y24	12.92
LP1 N 21	LV1 Y24	13.03	LP1 N21	LV2 Y24	11.63
LP1 N 21	LV2 Y24	11.60	LP1 N21	LV5 Y24	6.85
LP1 N 21	LV5 Y24	6.42			
LP2 N 21	LV4 Y24	3.72			

1.9.3 EPR Parameters

Table S25. Calculated EPR parameters and electron densities for the flv$^{1-\bullet}$ anion in [K(crypt-222)](flv•) (**1**), given for SARC and DKH approaches. The CPCM solvent model for THF was used for these calculations.

	**Isotropic Hyperfine Coupling Constants (MHz)**		**Electron Density at Nucleus**	
Atom No.	SARC	DKH	SARC	DKH
N1	103.872	108.981	201.68	210.86
C2	-26.372	-27.398	123.63	127.84
C3	-62.791	-65.170	123.64	127.85
C4	-26.381	-27.408	123.63	127.84
C5	-2.387	-2.490	123.65	127.86
N6	103.896	109.007	201.68	210.86
C7	-62.791	-65.169	123.64	127.85
N8	103.896	109.005	201.68	210.86
C9	-2.349	-2.451	123.65	127.86
H10	-2.548	-2.548	0.43	0.43
C11	4.479	4.631	123.64	127.85
C12	-26.382	-27.408	123.63	127.84

N13	103.874	108.982	201.68	210.86
H14	-2.554	-2.555	0.43	0.43
C15	4.427	4.578	123.64	127.85
H16	-3.891	-3.891	0.43	0.43
C17	-26.373	-27.399	123.63	127.84
C18	-2.349	-2.451	123.65	127.86
H19	-3.884	-3.884	0.43	0.43
C20	-2.387	-2.490	123.65	127.86
H21	-2.554	-2.555	0.43	0.43
C22	4.427	4.578	123.64	127.85
H23	-2.548	-2.548	0.43	0.43
C24	4.479	4.631	123.64	127.85
H25	-3.884	-3.884	0.43	0.43
H26	-3.891	-3.891	0.43	0.43

Color-coded are positions of the C and H atoms of the free flv$^{1-•}$ ligand. Green: Inner position, Blue: Terminal position, Yellow: Central C–C bond.

Table S26. Calculated EPR parameters and electron densities for [(Cp$_2$Y)$_2$(μ-flv•)]$^+$ (**2'**), a contracted model of [(Cp*$_2$Y)$_2$(μ-flv•)][Al(OC{CF$_3$}$_3$)$_4$] (**2**), given for SARC and DKH approaches. The CPCM solvent model for difluorobenzene was used for these calculations.

	**Isotropic Hyperfine Coupling Constants (MHz)**		**Electron Density at Nucleus**	
Atom No.	SARC	DKH	SARC	DKH
Y1	3.375	5.890	60233.34	96674.60
Y32	3.389	5.914	60233.33	96674.59
N2	7.556	7.931	201.51	210.67
N3	7.534	7.898	201.51	210.67
N33	7.501	7.872	201.51	210.67
N34	7.591	7.967	201.51	210.67
C5	-54.297	-56.363	123.63	127.84
C15	-9.685	-10.096	123.62	127.83
C16	-9.301	-9.704	123.62	127.83
C18	-54.351	-56.411	123.63	127.84
C28	-9.253	-9.646	123.62	127.83
C29	-16.182	-16.796	123.65	127.86
C30	-9.716	-10.127	123.62	127.83
C31	-16.264	-16.879	123.65	127.86
C35	-16.464	-17.098	123.65	127.86
C36	9.301	9.629	123.66	127.87
C37	-16.198	-16.818	123.65	127.86
C38	9.904	10.252	123.66	127.87
C49	37.873	38.361	115.38	116.57
C50	9.892	10.251	123.66	127.87
H61	-0.716	-0.716	0.427	0.427
H62	-4.560	-4.561	0.428	0.428
H63	-0.717	-0.717	0.426	0.426

H64	-0.729	-0.730	0.426	0.426
H65	-4.640	-4.640	0.429	0.429
H66	-4.637	-4.638	0.429	0.429
H67	-0.734	-0.734	0.426	0.426
H68	-4.623	-4.624	0.429	0.429

Color-coded are positions of the C and H atoms of the bridging flv ligand. Green: Inner position, Blue: Terminal position, Yellow: Central C–C bond.

Table S27. Calculated EPR parameters and electron densities for $[(Cp_2Y)_2(\mu\text{-flv}\bullet)]^-$ (**4'**), a contracted model of [K(crypt-222)]$[(Cp^*_2Y)_2(\mu\text{-flv}\bullet)]$ (**4**), given for SARC and DKH approaches. The CPCM solvent model for THF was used for these calculations.

	**Isotropic Hyperfine Coupling Constants (MHz)**		**Electron Density at Nucleus**	
Atom No.	SARC	DKH	SARC	DKH
Y1	1.040	1.809	60234	96675
Y22	1.095	1.903	60234	96675
N2	5.377	5.643	201.36	210.51
N3	5.301	5.564	201.36	210.51
N23	5.375	5.642	201.36	210.51
N24	5.301	5.564	201.36	210.51
C4	0.518	0.517	123.57	127.78
C6	-6.141	-6.370	123.60	127.81
C16	-6.016	-6.242	123.60	127.81
C17	0.509	0.507	123.57	127.78
C18	-6.014	-6.240	123.60	127.81
C19	2.360	2.447	123.62	127.83
C20	2.302	2.387	123.62	127.83
C21	-6.138	-6.368	123.60	127.81
C25	2.301	2.386	123.62	127.83
C26	-0.698	-0.726	123.62	127.83
C29	-0.642	-0.667	123.62	127.83
C30	2.358	2.445	123.62	127.83
C42	-0.641	-0.665	123.62	127.83
C45	-0.696	-0.723	123.62	127.83
H27	-2.306	-2.306	0.43	0.43
H28	-2.281	-2.281	0.43	0.43
H41	-2.281	-2.280	0.43	0.43
H43	-0.972	-0.973	0.43	0.43
H44	-1.004	-1.006	0.43	0.43
H46	-2.306	-2.305	0.43	0.43
H47	-1.005	-1.007	0.43	0.43
H48	-0.973	-0.975	0.43	0.43

Color-coded are positions of the C and H atoms of the bridging Bbim ligand. Green: Inner position, Blue: Terminal position, Yellow: Central C–C bond.

Table S28. Calculated EPR parameters and electron densities for $[(Cp_2Y)_2(\mu\text{-Bbim}\bullet)]^-$, a contracted model of $[K(crypt\text{-}222)][(Cp^*_2Y)_2(\mu\text{-Bbim}\bullet)]$, given for ZORA and DKH approaches.

	**Isotropic Hyperfine Coupling Constants (MHz)**		**Electron Density at Nucleus**	
Atom No.	ZORA	DKH	ZORA	DKH
Y1	3.344	5.824	60234	96675
Y24	0.619	1.086	60234	96675
N2	4.913	5.154	201.43	210.59
N3	4.854	5.093	201.43	210.59
N18	4.574	4.798	201.44	210.59
N21	4.442	4.660	201.44	210.59
C14	-1.532	-1.609	123.60	127.81
C15	-8.282	-8.595	123.64	127.85
C16	-1.377	-1.449	123.60	127.81
C17	-8.180	-8.488	123.64	127.85
C18	-8.160	-8.466	123.64	127.85
C20	5.853	6.067	123.60	127.81
C22	5.722	5.931	123.60	127.81
C23	-8.033	-8.336	123.64	127.85
C25	6.031	6.253	123.60	127.81
C26	-1.114	-1.157	123.61	127.82
C29	-1.049	-1.089	123.61	127.82
C30	5.971	6.191	123.60	127.80
C42	-1.321	-1.372	123.61	127.82
C45	-1.361	-1.413	123.61	127.82
H27	-5.074	-5.073	0.43	0.43
H28	-4.967	-4.967	0.43	0.43
H41	-5.108	-5.107	0.43	0.43
H43	-2.130	-2.131	0.43	0.43
H44	-2.122	-2.124	0.43	0.43
H46	-5.048	-5.047	0.43	0.43
H47	-2.015	-2.015	0.43	0.43
H48	-1.952	-1.953	0.43	0.43

Color-coded are positions of the C and H atoms of the bridging Bbim ligand. Green: Inner position, Blue: Terminal position, Yellow: Central C–C bond.

1.9.4 TDDFT

Table S29. TDDFT-calculated transitions for flv$^{1-•}$ (**1**$^{-}$) on the ZORA-def2-TZVP(flv) level using the PBE0 functional with dispersion correction D3BJ and implicit solvent model CPCM for THF. The coordinates used for this calculation were obtained through optimization of the crystal coordinates on the def2-SVP(Cp)/def2-TZVP(Y,flv) level of theory with the TPSSh functional and dispersion correction and solvent model as mentioned above. The calculated excitation energies were empirically red-shifted by 0.3 eV. HOMO: 59a, SOMO: 60a, LUMO: 61a. Print threshold for individual transitions: 10%. Isovalue for orbital depictions: 0.03.

λ (nm)	$\tilde{\nu}$ (cm^{-1})	Oscillator strength	Dominant Contributions (>10%) Occupied	Dominant Contributions (>10%) Virtual	Weight	Assignment
714.5	13996.2	0.032	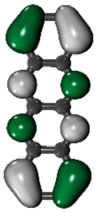 59b	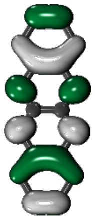 60b	0.94	
581.6	17193.7	0.133	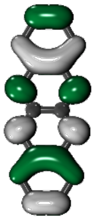 60a	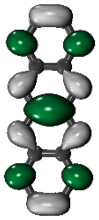 61a	0.83	HOMO → LUMO
			57b	60b	0.14	
423.0	23641.5	0.737	60a	61a	0.11	
			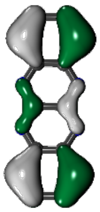 57b	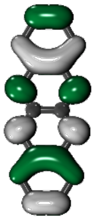 60b	0.78	HOMO−3 → HOMO
320.5	31205.0	0.230	59a	62a	0.29	
			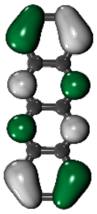 59b	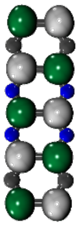 62b	0.64	

264.4	37824.8	2.531	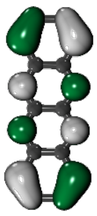<br>59a	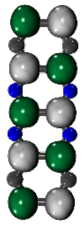<br>62a	0.61	HOMO−1 → LUMO
			59b	62b	0.27	
210.5	47515.0	0.438	56a	62a	0.38	
			57b	62b	0.34	
189.3	52830.8	0.266	57a	64a	0.37	
			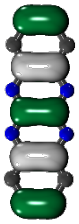<br>56b	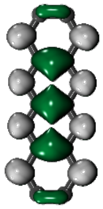<br>64b	0.50	
177.2	56422.2	0.318	53a	62a	0.42	
			57a	65a	0.23	
			56b	65b	0.13	
166.2	60185.3	0.189	53a	62a	0.13	
			56b	65b	0.16	
			59b	72b	0.23	

Table S30. TDDFT-calculated transitions for $[(Cp_2Y)_2(\mu\text{-flv}\cdot)]^+$ (**2'**) on the ZORA-def2-SVP(Cp)/SARC-ZORA-def2-TZVP(Y)/ZORA-def2-TZVP (flv) level using the PBE0 functional with dispersion correction D3BJ and implicit solvent model CPCM for DCM. The coordinates used for this calculation were obtained through optimization of the crystal coordinates on the def2-SVP(Cp)/def2-TZVP(Y,flv) level of theory with the TPSSh functional and dispersion correction and solvent model as mentioned above. The calculated excitation energies were empirically red-shifted by 0.3 eV. HOMO: 167, SOMO: 168a, LUMO: 169. Oscillator strength cutoff value >350 nm: 0.005, <350 nm: 0.1. Print threshold for individual transitions: 10%. Donor/acceptor orbitals are visualized for the dominant transitions with weights >50%. Isovalue for orbital depictions: 0.03.

**λ (nm)**	**$\tilde{\nu}$ (cm^{-1})**	**Oscillator strength**	**Dominant Contributions (>10%)**			**Assignment**
			**Occupied**	**Virtual**	**Weight**	
834.6	11981.6	0.045867476	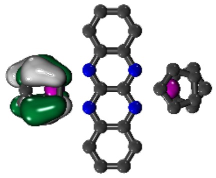<br>166b	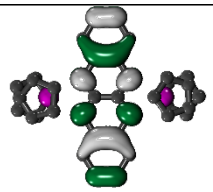<br>168b	0.90	MLCT

629.5	15884.4	0.014409832	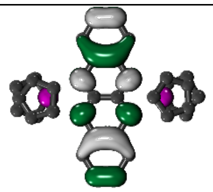<br>168a<br>158b	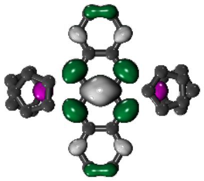<br>169a<br>168b	0.76<br>0.22	SOMO → LUMO<br><br>π →π*
561.0	17824.1	0.018045993	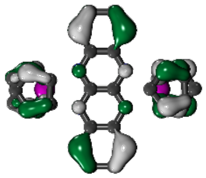<br>159b<br>160b	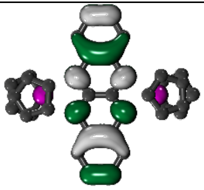<br>168b<br>168b	0.84<br>0.13	L/M π → L π*
478.0	20921.8	0.318216751	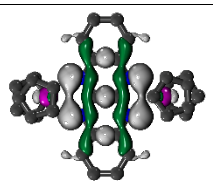<br>156b<br>158b	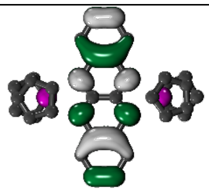<br>168b<br>168b	0.50<br>0.36	π →π*
471.8	21194.7	0.367006186	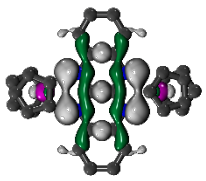<br>156b<br>158b	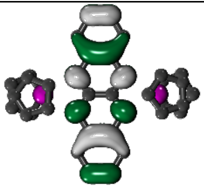<br>168b<br>168b	0.48<br>0.37	π →π*
365.9	27331.9	0.056785753	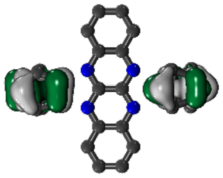<br>167a	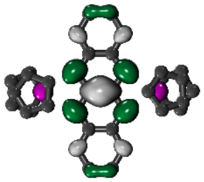<br>169a	0.94	HOMO → LUMO<br><br>MLCT
267.1	37433.0	1.012837223	163a<br>166b	170a<br>170b	0.24<br>0.21	

Table S31. TDDFT-calculated transitions for $[(Cp_2Y)_2(\mu\text{-flv}^\bullet)]^-$ (**4'**) on the ZORA-def2-SVP(Cp)/SARC-ZORA-def2-TZVP(Y)/ZORA-def2-TZVP (flv) level using the PBE0 functional with dispersion correction D3BJ and implicit solvent model CPCM for THF. The coordinates used for this calculation were obtained through optimization of the crystal coordinates on the def2-SVP(Cp)/def2-TZVP(Y,flv) level of theory with the PBE0 functional and dispersion correction and solvent model as mentioned above. The calculated excitation energies were empirically red-shifted by 0.3 eV. HOMO: 168, SOMO: 169a, LUMO: 170. Oscillator strength cutoff value >350 nm: 0.005, <350 nm: 0.1. Print threshold for individual transitions: 10%. Donor/acceptor orbitals are visualized for the dominant transitions with weights >50%. Isovalue for orbital depictions: 0.03.

λ (nm)	$\tilde{\nu}$ (cm^{-1})	Oscillator strength	Dominant Contributions (>10%) Occupied	Virtual	Weight	Assignment
949.7	10529.8	0.013113025	169a 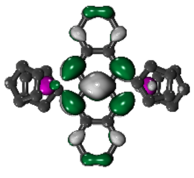	171a 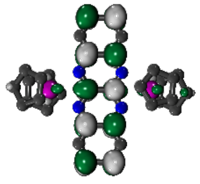	0.14<br>0.84	π →π*
682.4	14654.4	0.059730746	169a 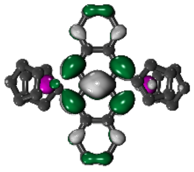	172a 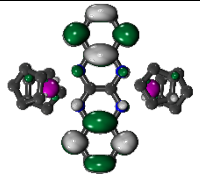	0.93	π →π*
629.1	15894.5	0.241768208	169a 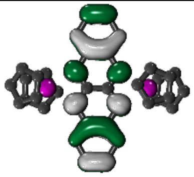	174a 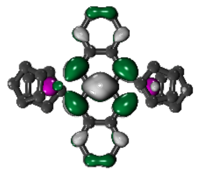	0.95	HOMO → SOMO<br>π →π*
498.5	20060.3	0.027431220	168b 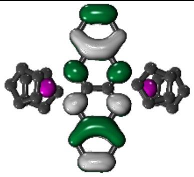	169b 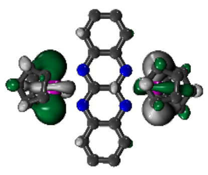	0.97	HOMO → LUMO<br>LMCT
431.6	23167.7	0.006220268	168b 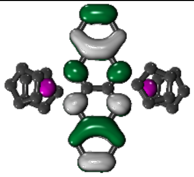	170b 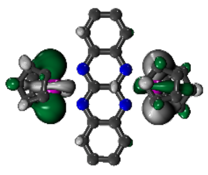	0.93	HOMO → LUMO<br>LMCT
408.3	24493.3	0.025869060	168a 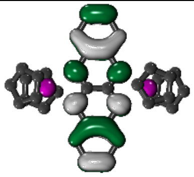	170a 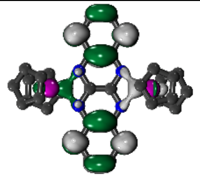	0.57	π →π*

			168b	173b		
			168a	173a	0.21	
			169a	184a	0.23	
391.0	25575.0	0.103612905	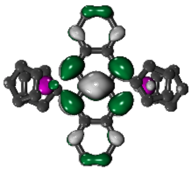	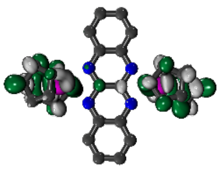	0.57	LMCT
			169a	185a		
390.9	25579.2	0.014242208	168a	172a	0.32	
			169a	183a	0.37	
347.5	28773.5	0.182147465	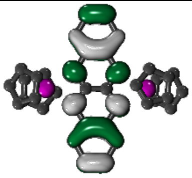	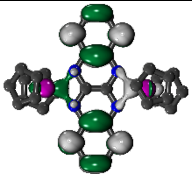	0.67	π →π*
			168a	173a		
			168b	173b	0.24	
284.7	35125.1	0.173426537	166a	170a	0.14	
			168a	177a	0.20	
			168b	177b	0.16	
279.8	35745.8	0.125644249	166a	170a	0.10	
			169a	197a	0.22	
			166b	170b	0.15	
264.1	37857.6	0.576696177	167a	172a	0.31	
			164b	169b	0.20	
			167b	172b	0.15	
			168b	187b	0.12	
260.9	38335.0	0.394281128	169a	203a	0.18	
			168b	184b	0.15	
260.5	38381.3	0.392606911	169a	203a	0.10	
			168b	187b	0.32	
237.0	42201.7	0.118773615	156a	170a	0.33	
			162a	170a	0.13	
			165a	172a	0.14	

1.9.5 Quantum Theory of Atoms in Molecules (QTAIM) Analysis

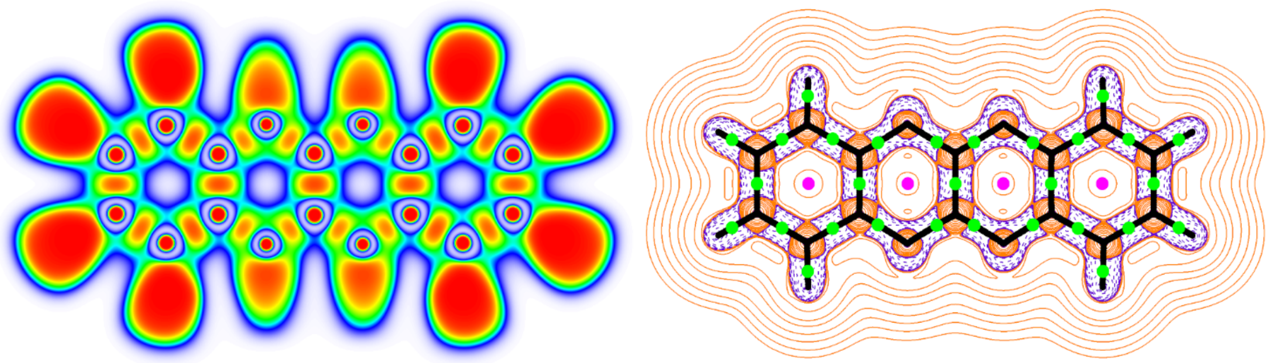

Figure S42. Electron localization function (ELF, left) and Laplacian of the electron density (right) maps of flv$^{1-\bullet}$ (**1**$^{-}$).

Table S32. Real space values of critical points as obtained from QTAIM analysis on flv$^{1-\bullet}$ (**1**$^{-}$).

Bond Critical Points (BCP, (3,−1))										
No	A−B	*ρ*(r) (E−01)	*G*(r) (E−01)	*K*(r) (E−01)	*V*(r) (E−01)	*H*(r) (E−01)	∇^2 (E−01)	*H*/*ρ*	*ε*(r) (E−01)	MBO
42	N^{13}–C^{7}	3.48	2.19	5.14	-7.32	-5.14	-11.8	-1.47	1.59	1.46
39	C^{17}–N^{13}	3.40	2.11	4.91	-7.02	-4.91	-11.2	-1.44	1.22	1.36
36	C^{20}–C^{17}	3.07	0.936	3.18	-4.12	-3.18	-8.98	-1.04	1.92	1.25
31	C^{24}–C^{20}	3.24	1.14	3.54	-4.68	-3.54	-9.58	-1.09	2.48	1.48
29	C^{22}–C^{24}	3.06	0.975	3.18	-4.15	-3.18	-8.81	-1.04	1.86	1.27
30	C^{22}–C^{18}	3.24	1.14	3.54	-4.68	-3.54	-9.58	-1.09	2.49	1.48
35	C^{18}–C^{12}	3.07	0.936	3.18	-4.12	-3.18	-8.98	-1.04	1.92	1.25
37	C^{12}–C^{17}	2.97	0.861	2.98	-3.84	-2.98	-8.49	-1.00	1.68	1.16
38	C^{12}–N^{6}	3.40	2.11	4.91	-7.02	-4.91	-11.2	-1.44	1.22	1.36
41	N^{6}–C^{3}	3.48	2.18	5.13	-7.32	-5.13	-11.8	-1.47	1.59	1.46
43	C^{3}–C^{7}	2.82	0.754	2.70	-3.45	-2.70	-7.78	-0.96	1.52	1.13
44	C^{3}–N^{1}	3.48	2.19	5.14	-7.32	-5.14	-11.8	-1.47	1.59	1.46
47	N^{1}–C^{2}	3.40	2.11	4.91	-7.02	-4.91	-11.2	-1.44	1.22	1.36
49	C^{2}–C^{4}	2.97	0.861	2.98	-3.84	-2.98	-8.49	-1.00	1.68	1.16
50	C^{2}–C^{5}	3.07	0.936	3.18	-4.12	-3.18	-8.98	-1.04	1.92	1.25
55	C^{5}–C^{11}	3.24	1.14	3.54	-4.68	-3.54	-9.58	-1.09	2.48	1.48
57	C^{11}–C^{15}	3.06	0.975	3.18	-4.15	-3.18	-8.81	-1.04	1.86	1.27
56	C^{9}–C^{15}	3.24	1.14	3.54	-4.68	-3.54	-9.58	-1.09	2.49	1.48
51	C^{4}–C^{9}	3.07	0.936	3.18	-4.12	-3.18	-8.98	-1.04	1.92	1.25
48	N^{8}–C^{4}	3.40	2.11	4.91	-7.02	-4.91	-11.2	-1.44	1.22	1.36
45	C^{7}–N^{8}	3.48	2.18	5.13	-7.32	-5.13	-11.8	-1.47	1.59	1.46

Ring Critical Points (RCP, (3,+1))											
	ρ(r) (E−02)	*G*(r) (E−02)	*K*(r) (E−03)	*V*(r) (E−02)	*H*(r) (E−03)	∇^2 (E−01)	*H*/*ρ* (E−01)	*ε*(r)	ELF (E−02)	$\lambda_{\pi 3}$ (E−02)	*SAI* (E−04)
33	2.31	3.00	-4.05	-2.60	4.05	1.36	1.75	-1.21	3.11	-1.56	5.08

40	2.73	3.60	-3.22	-3.28	3.22	1.57	1.18	-1.26	3.72	-2.24	33.1
46	2.73	3.60	-3.22	-3.28	3.22	1.57	1.18	-1.26	3.72	-2.24	33.1
53	2.31	3.00	-4.05	-2.60	4.05	1.36	1.75	-1.21	3.11	-1.56	5.08

ρ(r): Density of all electrons; *G*(r): Lagrangian kinetic energy; *K*(r): Hamiltonian kinetic energy; *V*(r): Potential energy; *H*(r): Energy density; ∇^2: Laplacian of electron density; *ε*: Ellipticity of electron density; ELF: Electron localization function; MBO: Mayer Bond Order; $\lambda_{\pi 3}$: Curvature of the electron density perpendicular to RCP; SAI: Shannon aromaticity index. Color−coded are the lateral in-plane C–C bonds of the flv^{1-}• molecule. Yellow: Central, Green: Inner, Blue: Terminal.

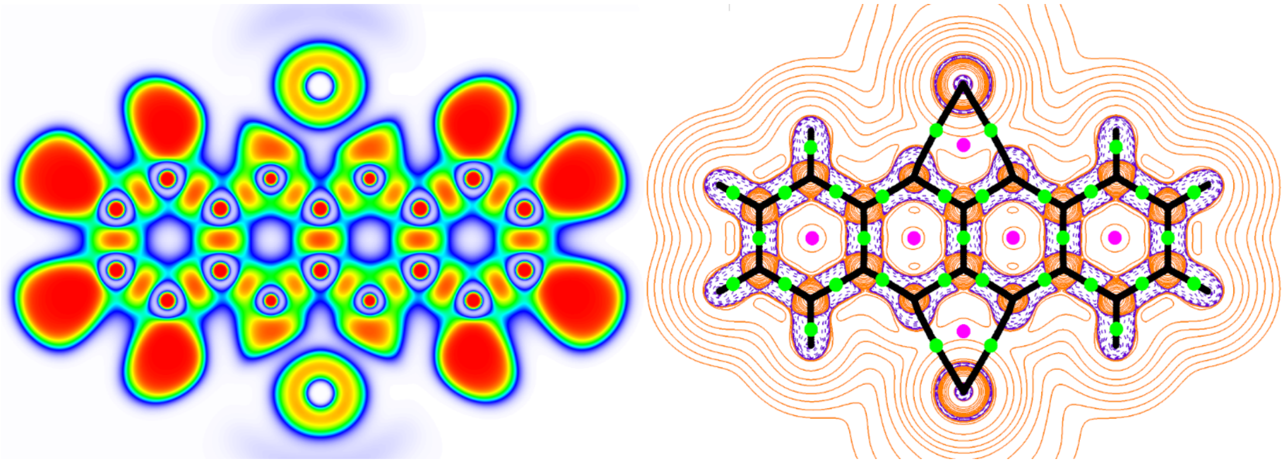

Figure S43. Electron localization function (ELF, left) and Laplacian of the electron density (right) maps of the $[(Cp_2Y)_2(\mu\text{-flv}^\bullet)]^+$ cation (**2'**).

Table S33. Real space values of critical points as obtained from QTAIM analysis on the $[(Cp_2Y)_2(\mu\text{-flv}^\bullet)]^+$ cation (**2'**).

Bond Critical Points (BCP, (3,−1))										
**No**	**A−B**	***ρ*(r) (E−01)**	***G*(r) (E−01)**	***K*(r) (E−01)**	***V*(r) (E−01)**	***H*(r) (E−01)**	**∇^2**	***H*/*ρ***	***ε*(r) (E−01)**	**MBO**
87	C^{16}–N^{3}	3.24	2.29	4.83	−7.12	−4.83	−1.01	−1.49	1.21	1.18
78	C^{31}–C^{16}	3.12	0.99	3.29	−4.28	−3.29	−0.92	−1.06	1.99	1.27
72	C^{38}–C^{31}	3.27	1.15	3.60	−4.75	−3.60	−0.98	−1.10	2.33	1.50
71	C^{38}–C^{50}	3.07	0.96	3.20	−4.16	−3.20	−0.90	−1.04	1.58	1.27
74	C^{50}–C^{37}	3.27	1.15	3.60	−4.75	−3.60	−0.98	−1.10	2.33	1.50
81	C^{37}–C^{30}	3.12	0.99	3.29	−4.28	−3.29	−0.92	−1.06	1.99	1.28
84	C^{16}–C^{30}	2.98	0.87	3.00	−3.87	−3.00	−0.85	−1.01	1.85	1.28
92	C^{30}–N^{34}	3.24	2.30	4.84	−7.14	−4.84	−1.01	−1.49	1.21	1.19
117	N^{34}–C^{18}	3.49	2.32	5.25	−7.57	−5.25	−1.17	−1.51	1.81	1.10
124	C^{5}–C^{18}	3.06	0.86	3.14	−3.99	−3.14	−0.91	−1.03	1.70	1.28
133	C^{18}–N^{33}	3.49	2.32	5.26	−7.58	−5.26	−1.17	−1.51	1.81	1.10
156	N^{33}–C^{28}	3.24	2.30	4.83	−7.13	−4.83	−1.01	−1.49	1.21	1.18
157	C^{15}–C^{28}	2.98	0.87	3.00	−3.86	−3.00	−0.85	−1.01	1.85	1.28
163	C^{28}–C^{35}	3.12	0.99	3.29	−4.28	−3.29	−0.92	−1.06	1.99	1.27
168	C^{35}–C^{49}	3.27	1.15	3.60	−4.75	−3.60	−0.98	−1.10	2.33	1.50

169	C^{36}–C^{49}	3.07	0.96	3.20	−4.16	−3.20	−0.90	−1.04	1.58	1.27
166	C^{29}–C^{36}	3.27	1.15	3.60	−4.75	−3.60	−0.98	−1.10	2.33	1.50
159	C^{15}–C^{29}	3.12	0.99	3.29	−4.28	−3.29	−0.92	−1.06	1.99	1.27
149	N^{2}–N^{15}	3.24	2.30	4.84	−7.14	−4.84	−1.01	−1.49	1.21	1.19
128	C^{5}–N^{2}	3.49	2.33	5.27	−7.60	−5.27	−1.17	−1.51	1.81	1.09
110	N^{3}–C^{5}	3.49	2.32	5.25	−7.57	−5.25	−1.17	−1.51	1.81	1.09
123	N^{34}–Y^{32}	0.48	0.44	0.038	−0.48	−0.038	0.16	−0.078	0.59	0.41
145	Y^{32}–N^{33}	0.48	0.43	0.036	−0.47	−0.036	0.16	−0.075	0.50	0.40
98	N^{3}–Y^{1}	0.48	0.44	0.037	−0.47	−0.037	0.16	−0.076	0.52	0.41
121	Y^{1}–N^{2}	0.48	0.44	0.037	−0.48	−0.037	0.16	−0.077	0.57	0.41

Ring Critical Points (RCP, (3,+1))											
	ρ(r) (E−02)	*G*(r) (E−02)	*K*(r) (E−03)	*V*(r) (E−02)	*H*(r) (E−03)	∇^2 (E−01)	*H*/*ρ* (E−01)	*ε*(r)	ELF (E−02)	$\lambda_{\pi3}$ (E−02)	*SAI* (E−04)
76	2.34	3.07	−4.15	−2.65	4.15	1.39	1.78	−1.20	3.10	−1.57	5.6
102	2.65	3.58	−3.61	−3.22	3.61	1.58	1.36	−1.25	3.44	−2.18	17.6
143	2.65	3.58	−3.61	−3.22	3.61	1.58	1.36	−1.25	3.44	−2.18	17.7
164	2.34	3.07	−4.15	−2.65	4.15	1.39	1.78	−1.20	3.10	−1.57	0.56
130	2.63	3.12	−1.49	−2.97	1.49	1.31	5.65	−1.35	4.40	−2.43	323.84
111	2.64	3.13	−1.48	−2.98	1.48	1.31	5.60	−1.35	4.41	−2.43	324.48

ρ(r): Density of all electrons; *G*(r): Lagrangian kinetic energy; *K*(r): Hamiltonian kinetic energy; *V*(r): Potential energy; *H*(r): Energy density; ∇^2: Laplacian of electron density; *ε*: Ellipticity of electron density; ELF: Electron localization function; MBO: Mayer Bond Order; $\lambda_{\pi3}$: Curvature of the electron density perpendicular to RCP; SAI: Shannon aromaticity index. Color-coded are the lateral in-plane C–C bonds of the bridging flv^{1-}• ligand. Yellow: Central, Green: Inner, Blue: Terminal.

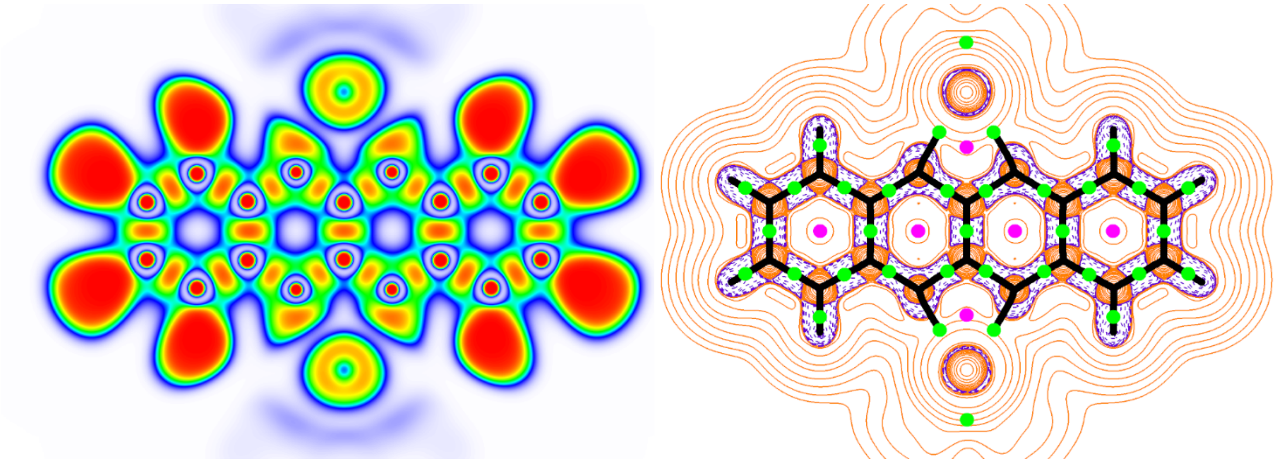

Figure S44. Electron localization function (ELF, left) and Laplacian of the electron density (right) maps of the $[(Cp_2Y)_2(\mu\text{-flv}\bullet)]^-$ anion (**4'**).

Table S34. Real space values of critical points as obtained from QTAIM analysis on the $[(Cp_2Y)_2(\mu\text{-flv}\bullet)]^-$ anion (**4'**).

Bond Critical Points (BCP, (3,−1))										
**No**	**A−B**	**ρ(r) (E−01)**	***G*(r) (E−01)**	***K*(r) (E−01)**	***V*(r) (E−01)**	***H*(r) (E−01)**	**∇² (E−01)**	***H*/*ρ***	***ε*(r) (E−01)**	**MBO**
115	N^3–C^4	3.21	1.73	4.20	−5.93	−4.20	−9.88	−1.31	1.67	0.96
109	C^{16}–N^3	3.13	1.78	4.21	−5.99	−4.21	−9.72	−1.34	1.09	1.06
103	C^{20}–C^{16}	3.16	1.05	3.36	−4.41	−3.36	−9.24	−1.06	2.45	1.36
91	C^{20}–C^{29}	3.13	1.05	3.32	−4.37	−3.32	−9.06	−1.06	2.22	1.35
102	C^{29}–C^{45}	3.18	1.09	3.41	−4.50	−3.41	−9.28	−1.07	2.33	1.40
113	C^{45}–C^{30}	3.13	1.05	3.31	−4.36	−3.31	−9.06	−1.06	2.22	1.35
116	C^{30}–C^{21}	3.16	1.05	3.36	−4.41	−3.36	−9.23	−1.06	2.45	1.36
114	C^{16}–C^{21}	2.99	0.90	3.03	−3.92	−3.03	−8.52	−1.01	2.05	1.31
119	C^{21}–N^{23}	3.13	1.78	4.21	−5.99	−4.21	−9.72	−1.34	1.09	1.07
124	N^{23}–C^{17}	3.21	1.74	4.22	−5.95	−4.22	−9.91	−1.31	1.67	0.96
121	C^4–C^{17}	3.28	1.14	3.59	−4.73	−3.59	−9.79	−1.10	3.13	1.46
127	C^{17}–N^{24}	3.21	1.73	4.20	−5.93	−4.20	−9.87	−1.31	1.67	0.96
133	N^{24}–C^{18}	3.13	1.78	4.21	−5.99	−4.21	−9.72	−1.34	1.09	1.06
139	C^{18}–C^{25}	3.16	1.05	3.36	−4.41	−3.36	−9.24	−1.06	2.45	1.36
150	C^{25}–C^{42}	3.13	1.05	3.32	−4.37	−3.32	−9.07	−1.06	2.22	1.35
140	C^{26}–C^{42}	3.18	3.18	1.09	3.41	−4.50	−3.41	−1.42	2.33	1.40
129	C^{19}–C^{26}	3.13	1.05	3.31	−4.36	−3.31	−9.06	−1.06	2.22	1.35
126	C^6–C^{19}	3.16	1.05	3.36	−4.41	−3.36	−9.24	−1.06	2.45	1.36
128	C^6–C^{18}	2.99	0.90	3.03	−3.92	−3.03	−8.52	−1.01	2.05	1.31
123	N^2–C^6	3.13	1.78	4.21	−5.99	−4.21	−9.72	−1.34	1.09	1.07
118	N^2–C^4	3.21	1.74	4.22	−5.96	−4.22	−9.91	−1.31	1.67	0.96
130	N^{23}–Y^{22}	0.66	0.63	0.090	−0.72	−0.090	2.17	−0.14	1.28	0.62
137	N^{24}–Y^{22}	0.66	0.63	0.090	−0.72	−0.090	2.16	−0.14	1.28	0.61
112	Y^1–N^2	0.66	0.63	0.090	−0.72	−0.090	2.16	−0.14	1.28	0.62
106	Y^1–N^3	0.66	0.63	0.090	−0.72	−0.090	2.16	−0.14	1.28	0.61

Ring Critical Points (RCP, (3,+1))											
	**ρ(r) (E−02)**	***G*(r) (E−02)**	***K*(r) (E−03)**	***V*(r) (E−02)**	***H*(r) (E−03)**	**∇² (E−01)**	***H*/*ρ* (E−01)**	***ε*(r)**	**ELF (E−02)**	**$\lambda_{\pi3}$ (E−02)**	***SAI* (E−04)**
134	2.33	3.10	−4.21	−2.63	4.21	1.39	1.81	−1.21	3.09	−1.50	1.91
125	2.53	3.38	−3.69	−3.01	3.69	1.50	1.46	−1.25	3.33	−2.00	4.04
117	2.53	3.38	−3.69	−3.01	3.69	1.50	1.46	−1.25	3.33	−2.00	4.04
108	2.33	3.05	−4.21	−2.63	4.21	1.39	1.81	−1.21	3.09	−1.50	1.91
111	3.22	4.25	−1.73	−4.0	1.73	1.77	0.54	−1.31	4.61	−2.68	2372
131	3.22	4.25	−1.73	−4.08	1.73	1.77	0.54	−1.31	4.61	−2.68	2371

ρ(r): Density of all electrons; *G*(r): Lagrangian kinetic energy; *K*(r): Hamiltonian kinetic energy; *V*(r): Potential energy; *H*(r): Energy density; ∇^2: Laplacian of electron density; *ε*: Ellipticity of electron density; ELF: Electron localization function; MBO: Mayer Bond Order; $\lambda_{\pi3}$: Curvature of the electron density perpendicular to RCP; SAI: Shannon aromaticity index. Color-coded are the lateral in-plane C–C bonds of the bridging $flv^{3-}\bullet$ ligand. Yellow: Central, Green: Inner, Blue: Terminal.

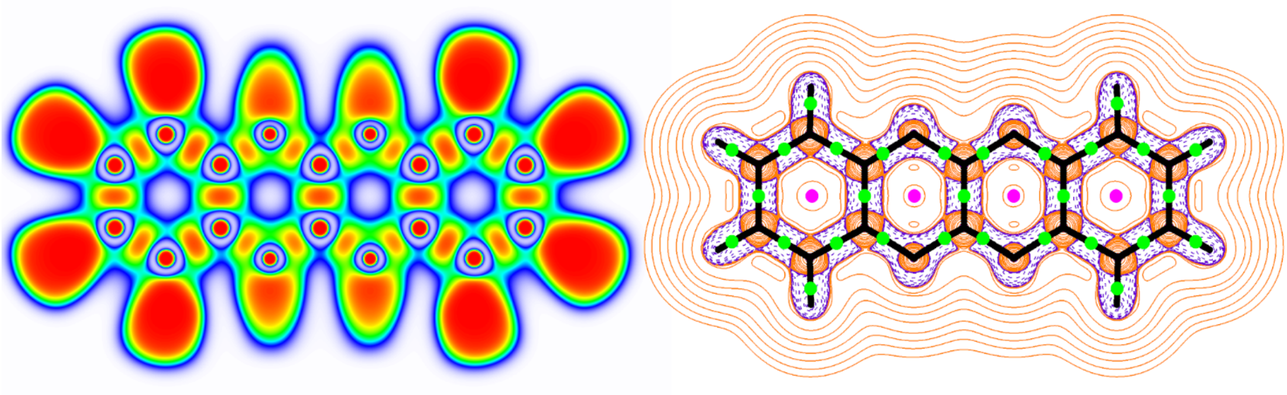

Figure S45. Electron localization function (ELF, left) and Laplacian of the electron density (right) maps of flv^{0}.

Table S35. Real space values of critical points as obtained from QTAIM analysis on flv^{0}.

Bond Critical Points (BCP, (3,−1))										
**No**	**A−B**	**ρ(r)**	**G(r) (E−01)**	**K(r) (E−01)**	**V(r) (E−01)**	**H(r) (E−01)**	**∇^2**	**H/ρ**	**ε(r)**	**MBO**
42	N^{13}–C^{7}	0.35	2.22	5.16	-7.39	-5.16	-1.17	-1.49	0.15	1.39
39	C^{17}–N^{13}	0.35	2.69	5.59	-8.27	-5.59	-1.16	-1.58	0.14	1.45
36	C^{20}–C^{17}	0.30	0.874	3.09	-3.97	-3.09	-0.89	-1.02	0.15	1.17
31	C^{24}–C^{20}	0.34	1.23	3.76	-4.99	-3.76	-1.01	-1.12	0.26	1.59
29	C^{22}–C^{24}	0.30	0.876	3.01	-3.89	-3.01	-0.85	-1.01	0.13	1.18
30	C^{22}–C^{18}	0.34	1.23	3.76	-4.99	-3.76	-1.01	-1.12	0.26	1.59
35	C^{18}–C^{12}	0.30	0.874	3.09	-3.97	-3.09	-0.89	-1.02	0.15	1.17
37	C^{12}–C^{17}	0.29	0.798	2.86	-3.65	-2.86	-0.82	-0.98	0.14	1.13
38	C^{12}–N^{6}	0.35	2.69	5.59	-8.28	-5.59	-1.16	-1.58	0.14	1.45
41	N^{6}–C^{3}	0.35	2.22	5.16	-7.38	-5.16	-1.17	-1.49	0.15	1.39
43	C^{3}–C^{7}	0.29	0.816	2.88	-3.70	-2.88	-0.83	-0.99	0.17	1.18
44	C^{3}–N^{1}	0.35	2.22	5.16	-7.39	-5.16	-1.17	-1.49	0.15	1.39
47	N^{1}–C^{2}	0.35	2.69	5.59	-8.27	-5.59	-1.16	-1.58	0.14	1.45
50	C^{2}–C^{5}	0.30	0.874	3.09	-3.97	-3.09	-0.89	-1.02	0.15	1.17
55	C^{5}–C^{11}	0.34	1.23	3.76	-4.99	-3.76	-1.01	-1.12	0.26	1.59
57	C^{11}–C^{15}	0.30	0.876	3.01	-3.89	-3.01	-0.85	-1.01	0.13	1.18
56	C^{9}–C^{15}	0.34	1.23	3.76	-4.99	-3.76	-1.01	-1.12	0.26	1.59
51	C^{4}–C^{9}	0.30	0.874	3.09	-3.97	-3.09	-0.89	-1.02	0.15	1.17
49	C^{2}–C^{4}	0.29	0.798	2.86	-3.65	-2.86	-0.82	-0.98	0.14	1.13
48	N^{8}–C^{4}	0.35	2.69	5.59	-8.28	-5.59	-1.16	-1.58	0.14	1.45
45	C^{7}–N^{8}	0.35	2.22	5.16	-7.38	-5.16	-1.17	-1.49	0.15	1.39

Ring Critical Points (RCP, (3,+1))											
	**ρ(r) (E−02)**	**G(r) (E−02)**	**K(r) (E−03)**	**V(r) (E−02)**	**H(r) (E−03)**	**∇^2**	**H/ρ**	**ε(r)**	**ELF (E−02)**	**$\lambda_{\pi3}$ (E−02)**	**SAI (E−03)**
33	2.29	2.98	-3.97	-2.58	3.97	0.13	0.17	-1.22	3.09	-1.60	1.66
40	2.80	3.73	-3.24	-3.41	3.24	0.16	0.12	-1.26	3.79	-2.35	3.62
46	2.80	3.73	-3.24	-3.41	3.24	0.16	0.12	-1.26	3.79	-2.35	3.62

53	2.29	2.98	-3.97	-2.58	3.97	0.13	0.17	-1.22	3.09	-1.60	1.66

ρ(r): Density of all electrons; *G*(r): Lagrangian kinetic energy; *K*(r): Hamiltonian kinetic energy; *V*(r): Potential energy; *H*(r): Energy density; ∇^2: Laplacian of electron density; *ε*: Ellipticity of electron density; ELF: Electron localization function; MBO: Mayer Bond Order; $\lambda_{\pi 3}$: Curvature of the electron density perpendicular to RCP; SAI: Shannon aromaticity index. Color−coded are the in−plane C–C bonds of the flv^0 molecule. Yellow: Central, Green: Inner, Blue: Terminal.

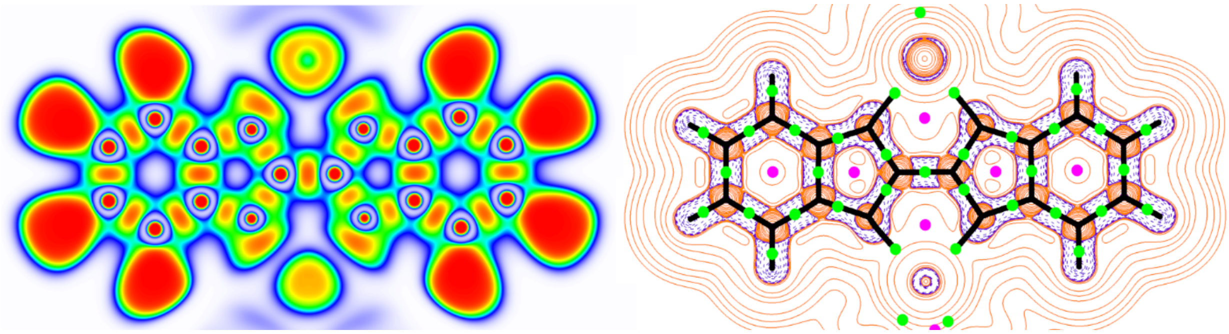

Figure S46. Electron localization function (ELF, left) and Laplacian of the electron density (right) maps of the $[(Cp_2Y)_2(\mu\text{-Bbim}\bullet)]^-$ anion.

Table S36. Real space values of critical points as obtained from QTAIM analysis on the $[(Cp_2Y)_2(\mu\text{-Bbim}\bullet)]^-$ anion.

**Bond Critical Points (BCP, (3,−1))**										
**No**	**A−B**	***ρ*(r) (E−01)**	***G*(r) (E−01)**	***K*(r) (E−01)**	***V*(r) (E−01)**	***H*(r) (E−01)**	**∇^2 (E−01)**	***H*/*ρ***	***ε*(r) (E−01)**	**MBO**
119	C^{16}–N^{21}	3.21	1.75	4.16	−5.91	−4.16	−9.66	−1.30	2.00	1.06
116	C^{23}–N^{21}	3.17	1.92	4.34	−6.26	−4.34	−9.69	−1.37	1.23	1.17
109	C^{30}–C^{23}	3.16	1.06	3.38	−4.44	−3.38	−9.27	−1.07	2.34	1.51
98	C^{45}–C^{30}	3.10	1.04	3.26	−4.30	−3.26	−8.88	−1.05	2.29	1.53
79	C^{29}–C^{45}	3.13	1.06	3.32	−4.38	−3.32	−9.04	−1.06	2.33	1.58
76	C^{29}–C^{22}	3.10	1.04	3.26	−4.30	−3.26	−8.87	−1.05	2.29	1.53
84	C^{22}–C^{17}	3.15	1.06	3.37	−4.43	−3.37	−9.26	−1.07	2.34	1.50
104	C^{17}–C^{23}	3.05	0.916	3.11	−4.03	−3.11	−8.78	−1.02	1.95	1.28
100	C^{17}–N^{3}	3.17	1.93	4.35	−6.28	−4.35	−9.68	−1.37	1.23	1.17
114	N^{3}–C^{16}	3.19	1.74	4.14	−5.88	−4.14	−9.60	−1.30	1.98	1.06
121	C^{16}–C^{14}	3.15	0.998	3.33	−4.33	−3.33	−9.32	−1.06	3.09	1.00
123	N^{2}–C^{14}	3.20	1.75	4.16	−5.91	−4.16	−9.64	−1.30	1.98	1.05
125	N^{2}–C^{15}	3.18	1.94	4.37	−6.31	−4.37	−9.71	−1.38	1.24	1.18
135	C^{15}–C^{20}	3.15	1.06	3.37	−4.43	−3.37	−9.26	−1.07	2.34	1.51
143	C^{20}–C^{26}	3.10	1.04	3.26	−4.30	−3.26	−8.87	−1.05	2.29	1.53
161	C^{26}–C^{42}	3.13	1.06	3.32	−4.38	−3.32	−9.04	−1.06	2.33	1.58
166	C^{25}–C^{42}	3.10	1.04	3.26	−4.30	−3.26	−8.87	−1.05	2.29	1.53
157	C^{19}–C^{25}	3.15	1.06	3.38	−4.43	−3.38	−9.26	−1.07	2.34	1.52
137	C^{15}–C^{19}	3.05	0.916	3.11	−4.03	−3.11	−8.78	−1.02	1.95	1.28
142	N^{18}–C^{19}	3.17	1.92	4.34	−6.26	−4.34	−9.68	−1.37	1.23	1.17

129	C^{14}–N^{18}	3.20	1.75	4.16	−5.90	−4.16	−9.64	−1.30	1.99	1.06
102	Y^{1}–N^{3}	0.627	0.577	0.0817	−0.659	−0.0817	1.99	−0.130	1.29	0.57
113	Y^{1}–N^{2}	0.636	0.584	0.0845	−0.669	−0.0845	2.00	−0.133	1.29	0.58
140	N^{18}–Y^{24}	0.625	0.577	0.0804	−0.658	−0.0804	1.99	−0.129	1.07	0.57
126	N^{21}–Y^{24}	0.622	0.575	0.0792	−0.654	−0.0792	1.99	−0.127	1.09	0.56

Ring Critical Points (RCP, (3,+1))											
	***ρ*(r) (E−02)**	***G*(r) (E−02)**	***K*(r) (E−03)**	***V*(r) (E−02)**	***H*(r) (E−03)**	**∇^2 (E−01)**	***H*/*ρ* (E−01)**	***ε*(r)**	**ELF (E−02)**	**$\lambda_{\pi 3}$ (E−02)**	***SAI* (E−04)**
93	2.36	3.07	−3.98	−2.67	3.98	1.39	1.69	−1.22	3.18	−1.58	0.750
110	5.65	8.59	−1.63	−8.42	1.63	3.50	0.289	−1.30	7.18	−5.72	1.67
131	5.65	8.59	−1.65	−8.43	1.65	3.50	0.291	−1.30	7.18	−5.72	1.71
151	2.36	3.07	−3.98	−2.67	3.98	1.39	1.69	−1.22	3.18	−1.58	0.754
111	1.63	1.81	−2.14	−1.60	2.14	0.811	1.31	−1.34	2.69	−1.08	1975
128	1.65	1.86	−2.24	−1.64	2.24	0.833	1.36	−1.26	2.64	−0.997	2000

ρ(r): Density of all electrons; *G*(r): Lagrangian kinetic energy; *K*(r): Hamiltonian kinetic energy; *V*(r): Potential energy; *H*(r): Energy density; ∇^2: Laplacian of electron density; *ε*: Ellipticity of electron density; ELF: Electron localization function; MBO: Mayer Bond Order; $\lambda_{\pi 3}$: Curvature of the electron density perpendicular to RCP; SAI: Shannon aromaticity index. Color−coded are the in−plane C–C bonds of the bridging $Bbim^{3-\bullet}$ ligand. Yellow: Central, Green: Inner, Blue: Terminal.

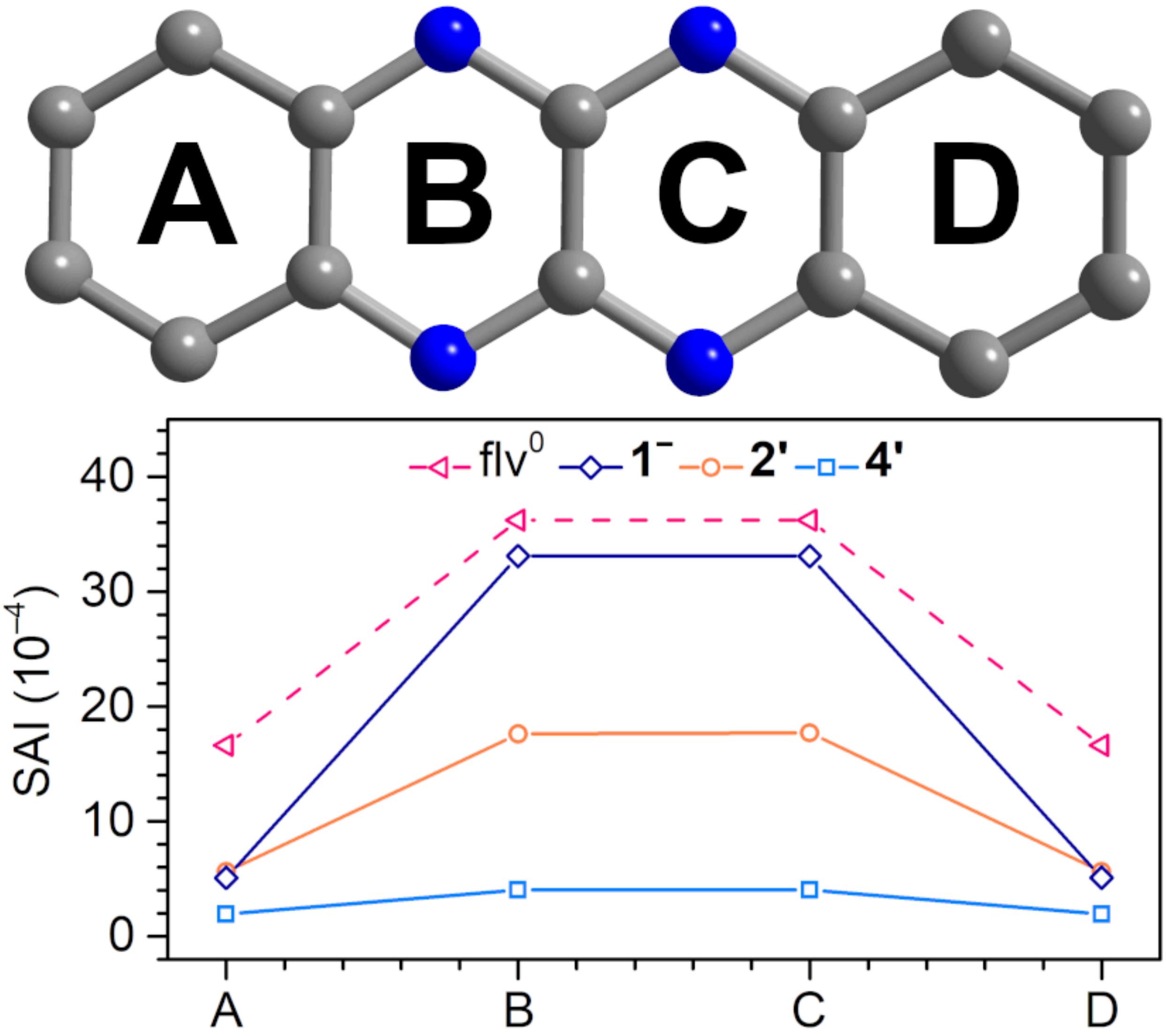

Figure S47. Graphical representation of the Shannon aromaticity index (SAI) as an aromaticity descriptor obtained via QTAIM analysis at BCP of flv^0 and model compounds **1**$^−$, **2'** and **4'**. Smaller SAIs indicate higher aromaticity, with a cutoff value for aromaticity/antiaromaticity of 0.003 < SAI < 0.005.

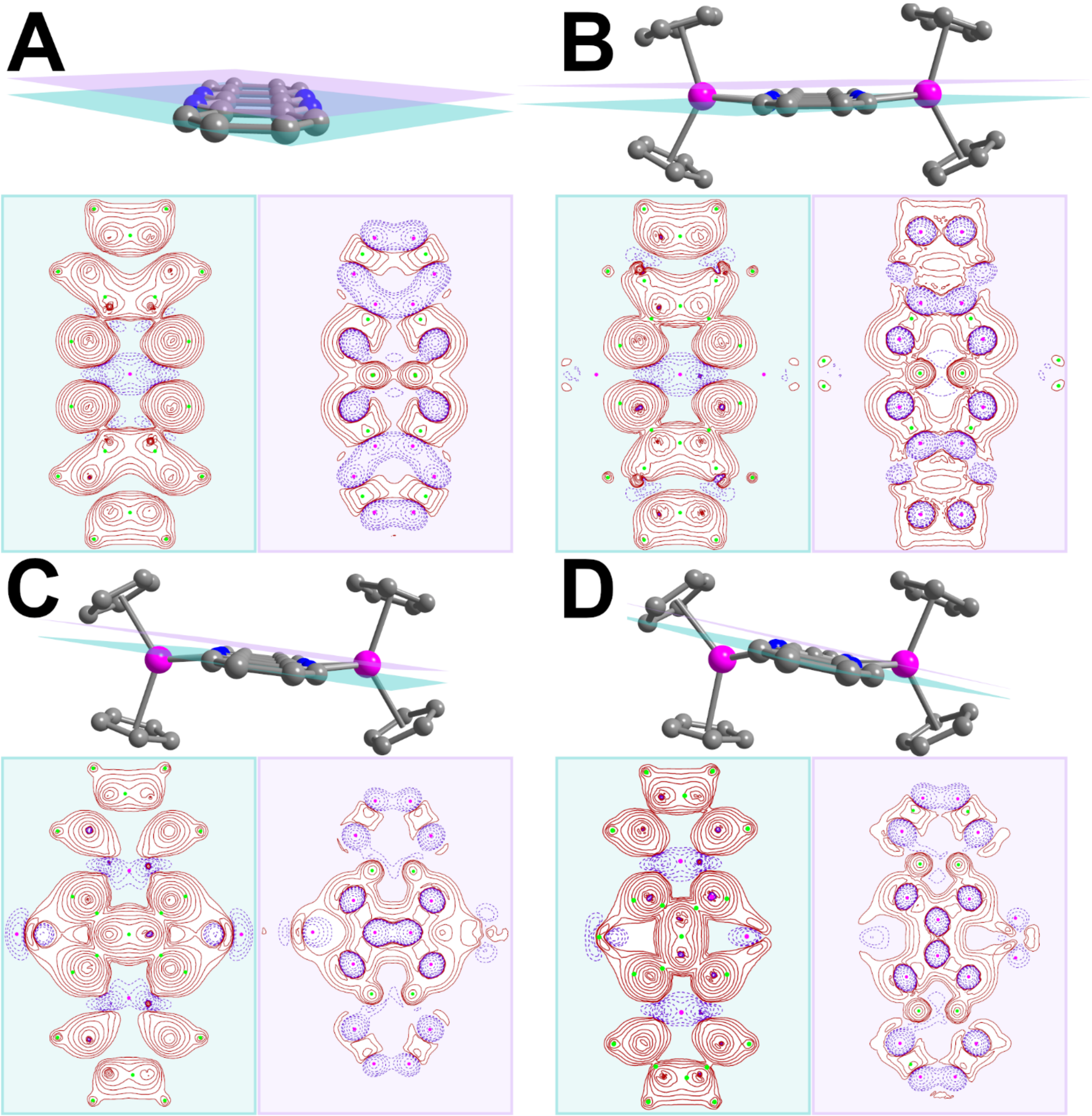

Figure S48. Plots of the Laplacian of the spin density $\nabla^2 s(\mathrm{r})$ for flv$^{1-\bullet}$ (**1**$^-$, **A**), $[(Cp_2Y)_2(\mu\text{-flv}^\bullet)]^+$ (**2'**, **B**), $[(Cp_2Y)_2(\mu\text{-flv}^\bullet)]^-$ (**4'**, **C**) and $[(Cp_2Y)_2(\mu\text{-Bbim}^\bullet)]^-$ (**D**). Pink and green spheres represent (3,−3) and (3,+3) critical points of the spin density, respectively. (3,−3) cps represent maxima of positive spin density, and (3,+3) cps minima of negative spin density. In-plane (green) and out-of-plane (purple) depictions were obtained by plotting $\nabla^2 s(\mathrm{r})$ through the N atoms and through the (3,+3) critical points above the N atoms, respectively.

Table S37. Real space values of critical points as obtained from spin density topology analysis on the flv$^{1-•}$ anion (**1**$^{-}$). SPI in the absence of spin polarization: 0.984.

**(3,−3) Critical Points**						**(3,+3) Critical Points**					
**No.**	ρ_α	ρ_β	$SPI(r)$	***s*(r) (E−02)**	**∇^2s(r)**	**No.**	ρ_α	ρ_β	$SPI(r)$	***s*(r) (E−03)**	**∇^2s(r) (E−02)**
N atoms						N atoms					
28	0.676	0.612	1.086	6.38	-1.63	4	0.138	0.139	0.978	-0.84	4.09
23	0.676	0.612	1.086	6.38	-1.81	8	0.138	0.139	0.978	-0.84	4.14
25	0.676	0.612	1.086	6.38	-1.75	77	0.138	0.139	0.978	-0.84	4.14
22	0.675	0.612	1.086	6.38	-1.72	73	0.138	0.139	0.978	-0.84	4.10
64	0.675	0.612	1.086	6.38	-1.78	CN outer					
62	0.676	0.612	1.086	6.38	-1.76	16	0.109	0.111	0.967	-1.89	2.72
60	0.676	0.612	1.086	6.38	-1.83	5	0.109	0.111	0.967	-1.9	2.65
53	0.676	0.612	1.086	6.38	-1.66	20	0.109	0.111	0.967	-1.9	2.67
Central C–C						9	0.109	0.111	0.967	-1.9	2.65
58	0.141	0.141	0.986	0.040	-0.014	66	0.109	0.111	0.967	-1.9	2.66
lateral C–C						57	0.109	0.111	0.967	-1.9	2.67
						54	0.109	0.111	0.967	-1.9	2.64
52	0.116	0.112	1.025	0.47	-0.056	43	0.109	0.111	0.967	-1.9	2.71
31	0.116	0.112	1.025	0.47	-0.057	Lateral C–C					
60	0.676	0.612	1.086	0.64	-1.83						
41	0.116	0.112	1.025	0.47	-0.054	40	0.182	0.182	0.979	-0.86	3.9
48	0.116	0.112	1.025	0.47	-0.057	71	0.182	0.182	0.979	-0.86	3.76
35	0.116	0.112	1.025	0.47	-0.056	45	0.182	0.182	0.979	-0.86	3.94
37	0.116	0.112	1.025	0.47	-0.054	29	0.182	0.182	0.979	-0.86	3.75
27	0.116	0.112	1.025	0.47	-0.056	Central C–C					
						61	0.273	0.281	0.953	-8.65	14.7
						38	0.272	0.281	0.953	-8.65	15.5
						7	0.272	0.281	0.953	-8.65	15.4
						3	0.273	0.281	0.953	-8.65	14.8

Table S38. Real space values of critical points as obtained from spin density topology analysis on the $[(Cp_2Y)_2(\mu\text{-flv}\bullet)]^+$ cation (**2'**). SPI in the absence of spin polarization: 0.993.

(3,−3) Critical Points

**No.**	ρ_α	ρ_β	$SPI(r)$	***s*(r) (E−02)**	**∇^2s(r)**
N atoms					
115	0.724	0.667	1.077	5.66	-1.69
89	0.672	0.614	1.086	5.73	-1.34
111	0.717	0.661	1.078	5.65	-1.31
88	0.677	0.620	1.084	5.70	-1.76
86	0.719	0.662	1.078	5.66	-1.30
59	0.676	0.619	1.085	5.72	-1.40
82	0.717	0.661	1.078	5.65	-1.68
56	0.677	0.620	1.084	5.70	-1.45
Central C–C					
102	0.153	0.153	0.995	0.040	-0.014
Lateral C–C					
117	0.261	0.247	1.049	1.39	-0.23
50	0.259	0.245	1.049	1.39	-0.24
91	0.257	0.243	1.050	1.40	-0.26
28	0.262	0.248	1.048	1.39	-0.24
99	0.258	0.244	1.050	1.39	-0.23
39	0.262	0.248	1.048	1.39	-0.26
75	0.256	0.243	1.050	1.39	-0.26
24	0.263	0.249	1.048	1.38	-0.23
Y–N					
73	0.191	0.191	0.993	0.0015	-0.00094
133	0.192	0.192	0.993	0.0016	-0.00096
Y atoms					
70	0.351	0.351	0.993	0.0014	-0.012
139	0.348	0.348	0.993	0.0015	-0.013

(3,+3) Critical Points

**No.**	ρ_α	ρ_β	$SPI(r)$	***s*(r) (E−03)**	**∇^2s(r) (E−02)**
N atoms					
103	0.104	0.105	0.987	-0.65	2.40
97	0.105	0.106	0.987	-0.65	2.50
55	0.105	0.105	0.987	-0.65	2.40
35	0.104	0.105	0.987	-0.65	2.39
C–N outer					
78	0.115	0.116	0.985	-0.98	2.38
54	0.161	0.162	0.987	-0.93	1.12
44	0.114	0.115	0.985	-0.95	2.39
92	0.115	0.116	0.985	-0.98	2.37
64	0.161	0.162	0.987	-0.93	0.99
52	0.114	0.115	0.985	-0.95	2.44
114	0.115	0.116	0.984	-0.99	2.33
112	0.160	0.161	0.987	-0.93	0.94
17	0.114	0.115	0.985	-0.95	2.37
109	0.115	0.116	0.985	-0.97	2.39
100	0.161	0.162	0.987	-0.93	1.19
13	0.114	0.115	0.985	-0.95	2.39
Central C–C					
58	0.287	0.295	0.966	-7.96	14.8
105	0.287	0.294	0.966	-8.00	12.9
61	0.277	0.285	0.965	-7.95	14.6
58	0.287	0.295	0.966	-8.00	14.8
Lateral C–C					
107	0.147	0.149	0.986	-1.05	3.49
95	0.147	0.148	0.986	-1.05	3.61
Y1					
69	0.147	0.148	0.986	-0.12	0.16
43	0.392	0.392	0.993	-0.12	43
36	0.275	0.275	0.992	-0.17	0.19
22	0.276	0.276	0.992	-0.18	0.19
Y2					
136	0.391	0.391	0.993	-0.12	0.14
120	0.388	0.388	0.993	-0.12	0.16
135	0.279	0.279	0.992	-0.17	0.19
130	0.277	0.277	0.992	-0.18	0.19

Table S39. Real space values of critical points as obtained from spin density topology analysis on the $[(Cp_2Y)_2(\mu\text{-flv}^\bullet)]^-$ anion (**4'**). SPI in the absence of spin polarization: 0.993.

(3,−3) Critical Points

**No.**	ρ_α	ρ_β	$SPI(r)$	***s*(r) (E−02)**	**∇²s(r)**
N atoms					
100	0.767	0.722	1.055	4.53	-1.27
43	0.696	0.650	1.063	4.60	-1.31
103	0.761	0.716	1.055	4.51	-0.94
45	0.700	0.654	1.062	4.57	-0.96
137	0.697	0.650	1.063	4.60	-1.25
71	0.766	0.721	1.055	4.53	-1.26
128	0.699	0.654	1.062	4.57	-1.13
65	0.762	0.717	1.055	4.51	-1.12
Central C–C					
126	0.400	0.363	1.096	3.75	-0.79
6	0.260	0.219	1.178	4.09	-0.66
127	0.260	0.219	1.178	4.09	-0.69
7	0.400	0.362	1.096	3.75	-0.64
Lateral C–C					
110	0.138	0.138	0.993	0.0028	-0.0020
129	0.138	0.138	0.993	0.0028	-0.0020
Y–N					
42	0.160	0.158	1.008	0.23	-0.021
114	0.160	0.157	1.008	0.23	-0.021
Y atoms					
135	0.287	0.283	1.006	0.37	-0.046
51	0.238	0.237	0.998	0.12	-0.015
82	0.238	0.237	0.998	0.12	-0.014
73	0.287	0.283	1.006	0.37	-0.049

(3,+3) Critical Points

**No.**	ρ_α	ρ_β	$SPI(r)$	***s*(r) (E−03)**	**∇²s(r) (E−02)**
N atoms					
69	0.161	0.162	0.988	-0.84	5.85
63	0.159	0.160	0.988	-0.84	5.26
96	0.159	0.159	0.988	-0.84	5.32
95	0.161	0.162	0.988	-0.84	5.37
C–N center					
28	0.200	0.201	0.988	-1.02	2.64
29	0.200	0.201	0.988	-1.01	2.65
101	0.200	0.201	0.988	-1.01	2.64
54	0.200	0.201	0.988	-1.02	2.42
Central C–C					
107	0.162	0.166	0.971	-3.70	13.2
lateral C–C					
78	0.186	0.188	0.981	-2.22	2.86
39	0.189	0.191	0.981	-2.28	3.02
116	0.184	0.186	0.981	-2.23	3.07
55	0.180	0.182	0.981	-2.15	2.69
89	0.180	0.182	0.981	-2.15	2.69
41	0.184	0.186	0.981	-2.23	2.89
131	0.189	0.191	0.981	-2.28	3.47
60	0.186	0.188	0.981	-2.22	3.12
Y1					
118	0.297	0.297	0.992	-0.28	1.15
119	0.298	0.298	0.992	-0.28	1.05
136	0.419	0.419	0.992	-0.27	0.86
124	0.541	0.541	0.993	-0.15	0.50
90	0.364	0.364	0.992	-0.29	1.35
Y2					
105	0.542	0.542	0.993	-0.15	0.48
117	0.364	0.364	0.992	-0.29	1.30
92	0.298	0.298	0.992	-0.28	1.14
85	0.297	0.297	0.992	-0.28	1.07
83	0.419	0.419	0.992	-0.27	0.94

1.9.6 Coordinates of Optimized Structures

1.9.6.1 Flv$^{1-•}$ (**1**$^{-}$) uTPSSh-D3BJ/def2-TZVP for frequency, EPR, TDDFT, QTAIM.

N	7.775445	7.129060	1.044938
C	7.659424	8.062373	0.075903
C	8.309140	5.949099	0.690015
C	8.086195	7.812118	-1.274291
C	7.102864	9.326083	0.389831
N	8.429752	5.012015	1.644224
C	8.744913	5.693113	-0.690223
N	8.624272	6.630187	-1.644440
C	7.934467	8.839279	-2.237222
H	6.788276	9.489918	1.414920
C	6.968928	10.303681	-0.572899
C	8.967809	3.830077	1.274071
N	9.278580	4.513142	-1.045153
H	8.265095	8.625602	-3.248014
C	7.388536	10.058499	-1.897033
H	6.539513	11.266265	-0.313627
C	9.394581	3.579822	-0.076123
C	9.119519	2.802908	2.236998
H	7.280117	10.834007	-2.648730
C	9.951125	2.316105	-0.390055
H	8.788890	3.016586	3.247789
C	9.665433	1.583682	1.896806
H	10.265714	2.152270	-1.415143
C	10.085042	1.338501	0.572671
H	9.773836	0.808168	2.648498
H	10.514444	0.375911	0.313397

1.9.6.2 Flv^{0} uTPSSh-D3BJ/def2-TZVP for energy and QTAIM.

N	7.77703677878951	7.12751632639024	1.04064818442025
C	7.66216873406660	8.04724449163409	0.08743009981571
C	8.31204386431566	5.94745882280852	0.68104752134619
C	8.09318313656939	7.79436250792894	-1.27663938589519
C	7.10071012773599	9.31945410581653	0.40881503104476
N	8.43124175659692	5.01196928297752	1.63960056495378
C	8.74194132000395	5.69472827708105	-0.68127154197591
N	8.62274655141281	6.63021889342355	-1.63982395930125
C	7.94238504337042	8.82653878124747	-2.25078008681745
H	6.78613168860408	9.48472763077578	1.43167573175660
C	6.97601066344079	10.27403172303228	-0.55539031403436
C	8.96080814800015	3.84782681501619	1.27641673317435
N	9.27695247700527	4.51467230318570	-1.04087117365987
H	8.27175065602735	8.61486981572634	-3.26038045928313
C	7.40103639952734	10.02564763568878	-1.89677601795229

H	6.54923727584234	11.23965430481769	-0.31071557287479
C	9.39182339400800	3.59494507944696	-0.08765254898184
C	9.11160728352752	2.81565096955762	2.25055757463554
H	7.28691034302866	10.80901876200766	-2.63688243832239
C	9.95328461488866	2.32273640699830	-0.40903705218632
H	8.78224127688939	3.02731958756501	3.26015792834342
C	9.65295942427549	1.61654345676065	1.89655425255658
H	10.26786356483361	2.15746333196578	-1.43189767365889
C	10.07798592820680	1.36815959326426	0.55516875770485
H	9.76708741945991	0.83317304030263	2.63666109905072
H	10.50476212957327	0.40253805458036	0.31049474614093

1.9.6.3 [(Cp$_2$Y)$_2$(μ-flv•)]$^+$ (**2'**) uTPSSh-D3BJ/def2-SVP(Cp)/def2-TZVP(Y,flv) for frequency, NLMO and QTAIM.

Y	8.52929993151780	4.11400613288850	17.26163390720311
N	7.79831206668162	6.17197990025815	18.32395543146468
N	6.59250917690473	4.26936844308745	18.72160336779555
C	8.82794163074923	3.40664990499395	14.77494252634325
C	6.78850835693120	5.57188613641116	18.96765998825614
C	6.71822918559784	4.10448253494912	15.37303904172191
C	7.53350057562703	2.95882064722439	15.15163630166851
C	10.74433984844120	4.39277483696214	18.63177612536571
C	9.59199885955588	2.43141488816270	18.96492280076018
C	10.33041137156874	2.30564803316266	17.75783110053049
C	7.51091229662204	5.25828502904661	15.13764256605483
C	11.04385111737464	3.51966061081925	17.55192956744840
C	9.84378849158433	3.72222975849599	19.50301854178323
C	8.81737588813068	4.82769956898276	14.77351402318506
C	8.02398928570742	7.49065545866751	18.57630225381897
C	5.58197001548412	3.63980237772286	19.38247918557592
H	9.67232982558774	2.77258011872350	14.50721862723047
C	5.97233485896278	6.28491031705835	19.89808634377373
H	5.66205407667021	4.09680552729549	15.64146105897137
H	7.21028587984547	1.91880673728439	15.21245262738459
H	11.15459929743651	5.39181313485127	18.78287395783659
H	8.96575727774329	1.66172828292374	19.41757168237866
H	10.37806036385897	1.42077269992040	17.12333744300172
H	7.17010521909093	6.29184268884183	15.19579493616825
H	11.72943097008549	3.72585214198191	16.73004914144052
H	9.44626783729542	4.11411947100311	20.43898935608833
H	9.65186115554928	5.47189757300171	14.49412774974529
C	7.20630436978096	8.20629235333388	19.51488144213463
C	9.07004147099700	8.16708212974686	17.92151926018255
C	4.76037480775948	4.35669263291139	20.31653100995998
C	5.33656322933733	2.27185773457487	19.16131933346045
Y	4.46276890339560	7.64568694105894	21.88420458723257
N	6.18927512714498	7.57919541201525	20.16778460593349

N	4.97676671824888	5.67871965182795	20.55895581553797
C	7.47315498362951	9.56589255728701	19.76123720670192
C	9.30484927165787	9.49714061300608	18.18169926366035
C	3.73226622869383	3.67340054884474	20.99202801691699
C	4.32294289918030	1.63154669863887	19.83550429538169
C	4.51238568572927	6.66918055144153	24.29795417821604
C	1.96746202801139	8.34850283465595	22.07676289505051
C	2.69804879539955	9.55472017541573	21.88657247019819
C	2.04842791056862	7.59342534067248	20.87598875493333
C	2.83203491766286	8.32791591827151	19.94542275119550
C	3.22957393904788	9.54143214869459	20.56892390975111
C	5.84716674428406	8.50184814242343	23.91918605031392
C	6.53973382615640	7.34935050885138	23.45177999944892
C	5.71623760766989	6.21750323966720	23.68855303068591
C	4.59826760723696	8.07980568965652	24.44752354536685
C	8.50334539994159	10.19971374386011	19.10636492924796
C	3.51773984887601	2.33552427272625	20.75586172718954
H	3.68937912564298	6.03998451497732	24.63864270601896
H	1.41090047349321	8.07357849923910	22.97267575556133
H	2.79636376383087	10.36320808837640	22.61088978672501
H	1.56914191442944	6.63214428082011	20.68809049887974
H	3.05717415315401	8.02989932996034	18.92179653642977
H	3.81210184467107	10.33739165181753	20.10359459913921
H	6.22731528646853	9.52422511551998	23.91724481656923
H	7.54095629697978	7.33582189173120	23.02170916666622
H	5.97256355423898	5.18116214413797	23.46966337493964
H	3.84949703609823	8.72057268697047	24.91166599149167
H	6.84955443579265	10.09562550266135	20.47147748329094
H	8.70278580679614	11.24630323165816	19.29899468311720
H	9.67778229105911	7.61836466757773	17.21186740765602
H	5.96096902217788	1.74206738038448	18.45179912179058
H	4.13891317312470	0.57900476007707	19.66091346766938
H	2.72519865039100	1.81537545848059	21.27889848561933
H	3.12057506430838	4.22389172058401	21.69681878592386
H	10.11043092632568	10.01248628072034	17.67404860080832

1.9.6.4 $[(Cp_2Y)_2(\mu\text{-flv}\bullet)]^+$ (**2'**) uTPSSh-D3BJ/def2-SVP(Cp)/def2-TZVP(Y,flv)/CPCM(THF) for EPR and TDDFT.

Y	8.51955889675553	4.11235804863934	17.27110355547341
N	7.79135211367002	6.17036162623452	18.31607209702921
N	6.57750800957996	4.27615877499160	18.70922610850358
C	8.83171424355118	3.39437380206344	14.78580178022668
C	6.77664108346046	5.57662098510206	18.95488250300870
C	6.72383758253332	4.11813938424326	15.36262370077687
C	7.52706340902545	2.96244222152867	15.14987828356049
C	10.73984499512368	4.42552070963281	18.63109481627971

C	9.57793020654034	2.48096552245828	19.03107423381578
C	10.31381131061527	2.31165723162371	17.82671950633710
C	7.53305381733848	5.26326584087610	15.13240297091272
C	11.03451739712515	3.51532139159383	17.58028536632777
C	9.83677178496519	3.78816987153285	19.52600807326662
C	8.83742648807896	4.81678849813613	14.78012366734426
C	8.02497645784164	7.48690181941094	18.57119905094520
C	5.56294829737590	3.65036433139458	19.36678749181377
H	9.67788013717314	2.74921990242384	14.54920074661930
C	5.96022427550199	6.29294189901645	19.88048115005741
H	5.67171641304493	4.12367668524278	15.64817618889252
H	7.19779934004310	1.92652607890329	15.24320471085285
H	11.13150341239179	5.43854326132212	18.73127255140055
H	8.92202829831354	1.74088682147115	19.49155888114955
H	10.33529740998923	1.41403314498420	17.20792673439757
H	7.21111187805659	6.30182559217900	15.21163992410888
H	11.70211622518632	3.70112911338687	16.73836384803865
H	9.41960100169062	4.22429696465240	20.43408882779211
H	9.68974697979833	5.45314567577297	14.53814979309183
C	7.20528419927337	8.20698722135421	19.50501790956263
C	9.08082693968328	8.15567512842085	17.92332992062814
C	4.74168737945133	4.37083066691876	20.29898740713004
C	5.31587910433466	2.28248212295241	19.14462684271741
Y	4.47430606543283	7.65158149606721	21.87275955546736
N	6.18017167852812	7.58523366592577	20.14965990731237
N	4.96298933475690	5.69194469657763	20.54057150190974
C	7.47618001238039	9.56526342981122	19.75663554867793
C	9.32180224010558	9.48471998160558	18.18806590429010
C	3.71008805352487	3.69258638762858	20.97522006043530
C	4.29762430637832	1.64704334570932	19.81835385364611
C	4.56900427205228	6.64143360904304	24.27872581976705
C	1.96565031152428	8.31860669760698	22.10186584173089
C	2.67759124632863	9.53510815528796	21.89708361874291
C	2.03891549237811	7.56059897467838	20.90197628004396
C	2.79775566194105	8.30404370889218	19.95652138505375
C	3.18845780266491	9.52537909301521	20.57042474322251
C	5.88318507873038	8.48648599852446	23.90394143067848
C	6.57482606746266	7.34488774907838	23.40961312356986
C	5.76015995725517	6.20413244285226	23.64374436332252
C	4.64300169284949	8.04844603879516	24.44403815412015
C	8.51733871418548	10.19414186496661	19.10893652385822
C	3.49266861438760	2.35450118185898	20.73761587263800
H	3.74531259762803	6.01028085292900	24.58389001907889
H	1.44715232273107	8.02605178931994	23.01545183741480
H	2.79742596020646	10.33753142949990	22.62574381145839
H	1.59607370238500	6.57784081487473	20.73625954654633

H	3.03446363527285	7.99365723816359	18.93856025642896
H	3.78122308376541	10.31439067627294	20.10558835806042
H	6.24494659667053	9.51539334614182	23.88050669807550
H	7.55859164591271	7.34564171287123	22.94024300349128
H	6.00366204554091	5.17456403244161	23.38364573970272
H	3.88441300688360	8.67757535422234	24.90849871404514
H	6.84754654805237	10.09167224061101	20.46532537345802
H	8.72313181443538	11.24167631408611	19.30518918731162
H	9.68928303888175	7.60012581636982	17.21942655133028
H	5.94349310712225	1.75329065558480	18.43717184571417
H	4.11100969250430	0.59436773425520	19.64480024460944
H	2.69777766837839	1.83669287813684	21.26030090348838
H	3.10253342943403	4.24848087519131	21.67959875547005
H	10.13594441381231	9.99304738263673	17.68593702375831

1.9.6.5 $[(Cp_2Y)_2(\mu\text{-flv}\cdot)]^-$ (**4'**) uTPSSh-D3BJ/def2-SVP(Cp)/def2-TZVP(Y,flv) for frequency, NLMO and QTAIM.

Y	1.53733998666895	-2.47527399596361	13.59996227448176
N	1.74415119988897	-0.22954985127707	13.20593977753965
N	-0.35839845376529	-1.21580088347976	13.34118177525163
C	0.38098661169172	-0.05469815216270	13.28866509211099
C	2.52015470970704	-4.65279456306116	12.38675715916872
C	2.51474961391314	0.91630011774462	13.17435120290500
C	2.80787749332549	-2.08341292112013	15.90028242147152
C	3.23077124695680	-3.54936881872388	11.84408902510435
C	1.16712373489326	-4.54883381786099	11.96803681475681
C	2.92971228975667	-3.47358233421923	15.63265528683701
C	0.71747963959642	-3.02004539213139	16.06602238612864
C	1.63511025150498	-4.05372905309679	15.73356653430245
C	1.44250739918143	-1.80506339857415	16.16841905117848
C	2.31957988663880	-2.77355727844694	11.07495111104599
C	1.04633851920821	-3.38996694898763	11.15127315866946
C	-1.72918257796766	-1.07248210302658	13.43300558999757
C	-0.19992521817139	1.19518412089449	13.35879579305993
C	1.91027045838241	2.21280340134880	13.21453117171544
C	3.90752099291052	0.82891012169599	13.09720372728730
C	-2.55470022237723	-2.19904646035728	13.48465046742898
C	-2.33369601318882	0.22405566864577	13.47258521932683
Y	-1.35631298590787	3.61557247887183	13.04689824721029
N	-1.56320009587205	1.36991783778355	13.44054282326611
N	0.53948426973617	2.35638233581755	13.30639607013292
C	2.73589937454195	3.33933728441638	13.16340840776915
C	4.71271252227376	1.96904218371491	13.05147193336387
H	4.35325406796834	-0.16122882143121	13.06491175044523
H	-2.08206414780271	-3.17654806082301	13.45086642067110
C	-3.94427779225149	-2.08712436319596	13.56497070423151
C	-3.72650751305899	0.31140601719464	13.54944279055459

C	-2.33421127944944	5.79508820581155	14.25985754469463
C	-2.63114162002644	3.22572477469228	10.74816177496539
C	-3.04933564647387	4.69350537518155	14.80036522568433
C	-0.98229107325335	5.68651940903821	14.68100428363792
C	-2.74903355091883	4.61630752381144	11.01520406824852
C	-0.53849803030520	4.15677234690362	10.57948158945695
C	-1.45303318107343	5.19301622181964	10.91259919036953
C	-1.26679322951415	2.94366005276007	10.47851552709640
C	-2.14202302515055	3.91426020996656	15.57067172294627
C	-0.86671092331051	4.52672703609839	15.49717238655342
H	2.26333070926492	4.31685114325099	13.19781744810142
C	4.12544385180702	3.22740949105373	13.08283381918851
H	5.79085332876768	1.86536021697063	12.99112102810049
H	-4.55590242399829	-2.98229282429625	13.60157874910800
C	-4.53159935406948	-0.82877938930998	13.59563474926293
H	-4.17228389614581	1.30153912285232	13.58125456122079
H	4.73710026710043	4.12257646483445	13.04670168663697
H	-5.60976619065939	-0.72515825729700	13.65567833671202
H	-2.37958978645638	2.99208537537157	16.09990940793664
H	-4.11278813530276	4.49070142224662	14.66500783436122
H	-2.74930316647302	6.58285244216373	13.63140796589439
H	-0.17749502136273	6.38246848437563	14.43938373180673
H	0.04742505207666	4.15871533238249	15.96182368638011
H	-3.67118473153057	5.14970283850078	11.24815598401709
H	-3.43995904324137	2.49426248901778	10.76043469448644
H	-0.84686041835032	1.96024243241163	10.26943671967637
H	0.53767847255749	4.26588010688540	10.44020914258578
H	-1.20850858423773	6.24686087840850	11.05097187511642
H	0.13023446882660	-3.02522261464977	10.68790923226067
H	2.55333707592601	-1.85103534985884	10.54463228072543
H	0.36500075492837	-5.24714358271541	12.21173661306316
H	2.93890341834497	-5.43876825678626	13.01502591783555
H	4.29382285403553	-3.34306840037518	11.97729942359167
H	1.39351643407831	-5.10812372145802	15.59425101813721
H	3.85353163042405	-4.00446329977053	15.40055132677930
H	3.61478171132900	-1.34981957675245	15.88925507865801
H	1.01969924650175	-0.82286932424719	16.37746112199074
H	-0.35857183304677	-3.13198349948185	16.20400378529679

1.9.6.6 $[(Cp_2Y)_2(\mu\text{-flv}\bullet)]^-$ (**4'**) uTPSSh-D3BJ/def2-SVP(Cp)/def2-TZVP(Y,flv)/CPCM(THF) for EPR and TDDFT.

Y	1.56371115949211	-2.49009775728172	13.58042829871645
N	1.74487526597668	-0.22328636992989	13.17464509137356
N	-0.34905547525320	-1.21896971775648	13.31812547786346
C	0.38266299399870	-0.05324114092443	13.27462090773435
C	2.45122410253858	-4.70787381811601	12.40267451171626
C	2.51045916034219	0.92724304222747	13.15503225010307

C	2.85154339397263	-2.08080462420407	15.86526600052821
C	3.25970587434992	-3.65303580201251	11.89922510907230
C	1.12915459415652	-4.51574647505872	11.91879562614802
C	2.94265575846854	-3.47711566953182	15.61576371119655
C	0.74216526189300	-2.97201297055898	16.05711321762203
C	1.63646476107200	-4.02891471089181	15.73213408210442
C	1.49273922128734	-1.76972127783895	16.13874639086425
C	2.43898916866113	-2.81758388078841	11.09070811802736
C	1.12401087853947	-3.34980915013705	11.10293831785381
C	-1.71987367001801	-1.08212563125469	13.42932684937479
C	-0.20238392163173	1.19297381907942	13.37140132727114
C	1.90009970108519	2.22191502480145	13.21669765196651
C	3.90443471045608	0.84670942615487	13.06636345698361
C	-2.53977356923170	-2.21465705994994	13.47694432167822
C	-2.33019467416647	0.21254884614881	13.49140922893858
Y	-1.38343957474970	3.63017646889280	13.06655312249641
N	-1.56459946617410	1.36305215405702	13.47097216192179
N	0.52927344048592	2.35870655197948	13.32757795960188
C	2.72002104953381	3.35445036279685	13.16956552923710
C	4.70427248606295	1.99297069763649	13.02699734545055
H	4.35515912101313	-0.14056980037118	13.02191372833435
H	-2.06267457075289	-3.18937194303744	13.43059473644519
C	-3.93060217081512	-2.10946030067237	13.57275617689008
C	-3.72412447040323	0.29308695684361	13.58080274015914
C	-2.26603846247402	5.84975608022818	14.24459746751441
C	-2.67077840783610	3.22190296893150	10.78127223831919
C	-3.07887514787595	4.79709535783729	14.74557227095871
C	-0.94552330743604	5.65295145321768	14.73083575088967
C	-2.76167481699685	4.61814488797082	11.03128699758999
C	-0.56121341297205	4.11279278130550	10.59010861657322
C	-1.45535951083377	5.16974083814003	10.91532427390031
C	-1.31199128757826	2.91067286386363	10.50790224509397
C	-2.26238948999928	3.95827445759504	15.55489302890034
C	-0.94567374370530	4.48626397143383	15.54562387447087
H	2.24293790091770	4.32915420676732	13.21628991314810
C	4.11096363452261	3.24924535396497	13.07542236353060
H	5.78227988098523	1.89522150205207	12.95800545463234
H	-4.53784184991454	-3.00750477409966	13.60646495490336
C	-4.52389817891813	-0.85318075276864	13.62125324003776
H	-4.17480012848353	1.28035922616244	13.62592044332647
H	4.71820665291656	4.14729138671996	13.04181630647400
H	-5.60185866291118	-0.75542898305394	13.69098278871713
H	-2.58891692233426	3.06164249726846	16.08208529752262
H	-4.14453407667322	4.66287769408985	14.55333559672244
H	-2.59672856130070	6.66255482705164	13.59792452354647
H	-0.08449276852304	6.29067703046525	14.52428101432796

H	-0.08349487840503	4.06752568195646	16.06482664623061
H	-3.67242672099742	5.17021586467205	11.26600258686531
H	-3.49705654650874	2.50991072739528	10.80395766244146
H	-0.91245196302266	1.91849811988674	10.29678235093124
H	0.51528244081195	4.20623547260226	10.43994107328911
H	-1.18921512948932	6.21982464088194	11.04240431794196
H	0.25950686934389	-2.93430146719392	10.58501013200287
H	2.76160401165430	-1.92033502161019	10.56217238054515
H	0.27059347070657	-5.15609023219481	12.12751155175041
H	2.78578373172714	-5.51906555757101	13.04937610414591
H	4.32528907662106	-3.51519908374444	12.08931575721941
H	1.37050096189006	-5.07909306851365	15.60543192911171
H	3.85347912855230	-4.02908864733742	15.38110611091968
H	3.67768754344344	-1.36867059336916	15.84218590928319
H	1.09304659230153	-0.77754228330187	16.34956622557750
H	-0.33429408139477	-3.06559902800336	16.20747187296917

1.9.6.7 [$(Cp_2Y)_2$(μ-Bbim•)]$^-$ uTPSSh-D3BJ/def2-SVP(Cp)/def2-TZVP(Y,Bbim) for frequency NLMO and QTAIM.

Y	9.15952292308172	3.84446788315917	15.55152737784050
N	9.57573801568324	5.66704692650748	14.18105456065975
N	11.47571481712694	3.83110378197356	15.36799642940858
C	9.38900293502887	5.58595168505662	17.54874166343163
C	8.02376414166542	5.62546282295100	17.15972072616533
C	7.43611003635977	4.37614246253816	17.49141014492756
C	8.44190183185756	3.56232415623577	18.08375380176864
C	9.64744742637169	4.31407943371737	18.12083579490927
C	7.13616568874343	2.69155207759604	14.27520941561960
C	7.55235635700118	1.74450211302743	15.24935951059389
C	8.84955464524782	1.29146281786991	14.89111402924792
C	9.22892260741716	1.94871690718427	13.68846616758580
C	8.17097148839755	2.81076597860853	13.30724542271368
C	10.93053139977042	5.92192510171823	14.22637874959190
C	9.04055860291167	6.76154591399724	13.54438866532473
C	11.85228560328421	5.02693387522392	14.79075659575919
C	12.65839928970594	3.26337463153893	15.78144070254561
N	11.28884626426641	7.11935735515960	13.64373638041623
C	10.10079302814072	7.65492524217519	13.20287146125152
C	7.71940543846487	7.06942559104857	13.22423411604001
N	13.21329310370401	5.24696544477270	14.80592645563069
C	12.90772808541901	2.06118304215674	16.44116194282636
C	13.73256067426970	4.13496156452906	15.42744840500698
Y	13.55606918064139	6.88949392488563	13.19780183311028
C	9.83577698654637	8.84079152090343	12.52013779148272
C	7.46744165141993	8.26786196668967	12.54544529199279
H	6.91145710865426	6.39317020802317	13.48642695322489
H	12.09336686849872	1.39768808732437	16.71609070245317

C	14.23725528005077	1.72863644552819	16.72776768130851
C	15.05179751317064	3.78775706957091	15.71277293964539
C	14.29709613000726	9.43361766465389	13.28653166262109
C	15.50519966340418	8.69822621540731	13.15209371705134
C	15.70045286292620	7.95683067181219	14.34772749798487
C	14.62067095278593	8.24662512273959	15.22650831758784
C	13.75570576710533	9.15819217152238	14.57256441226141
C	14.18059999373096	4.84445462336858	11.62808181411784
C	14.92601996034292	5.95134771654680	11.14012236934207
C	14.00820533881434	6.89009408096035	10.59104058015291
C	12.69841863371334	6.36241110917869	10.74296809403847
C	12.80563072161141	5.09957958205167	11.38363577021740
H	10.63991899904046	9.51835019352569	12.25011026627849
C	8.50489503143993	9.13461424392254	12.19887760246066
H	6.44608207268347	8.52704731680130	12.28400941740298
H	14.45397080664875	0.79523204287682	17.23814003547438
C	15.28816968833178	2.57406175577245	16.36887433129502
H	15.87062539905392	4.44511205275208	15.43730484594467
H	8.27659560994653	10.05388105955782	11.66823174412056
H	16.30844138389816	2.28445221409340	16.60102469156626
H	13.86832311143504	10.10410530395439	12.54048564866378
H	16.16620435037685	8.70429829893522	12.28564197313299
H	16.53877542474080	7.29236441741741	14.56117249976010
H	16.01107175961507	6.05716470821367	11.16850631993217
H	12.82192197622844	9.55253432878201	14.97182261114496
H	14.46933475884237	7.81646648236632	16.21576032382527
H	11.76907431494771	6.84475660679458	10.43864806700236
H	14.26676466262501	7.84331316208541	10.12868641280018
H	14.58709538726456	3.95902503123351	12.11756251961024
H	11.97501773556884	4.45337544068262	11.66622342973507
H	10.17833555273607	1.82878445434951	13.16787464711004
H	9.44802630864906	0.55899670444560	15.43450438884920
H	6.97689920553511	1.42273024811454	16.11714547039335
H	6.18294344725376	3.22181396448245	14.26363908637945
H	8.16492721660495	3.47181992265029	12.44115901516791
H	10.60878562710061	3.97112730962900	18.50421733776183
H	10.11860584567536	6.38132699743260	17.39925064288071
H	7.52344486282319	6.46605924711059	16.67771068624682
H	6.39648652298342	4.09148241657974	17.32675414067917
H	8.30625163060619	2.54732752552543	18.45911703652137

1.9.6.8 $[(Cp_2Y)_2(\mu\text{-Bbim}^\bullet)]^-$ uTPSSh-D3BJ/def2-SVP(Cp)/def2-TZVP(Y,Bbim)/CPCM(THF) for EPR.

Y	9.15752873471013	3.84442174119740	15.54921411997520
N	9.57510688955714	5.66756661903162	14.17941335243433
N	11.47351655941367	3.83103234475744	15.36361644517054
C	9.38834592918427	5.58833975027534	17.54222913862312

C	8.02153854294427	5.62349406689357	17.15824922712347
C	7.43846879212787	4.37325075431844	17.49459976990088
C	8.44858772023286	3.56327832936815	18.08459595172902
C	9.65232412509532	4.31824007595612	18.11561155984912
C	7.13064323708786	2.68629889733504	14.28133490695064
C	7.55204820161100	1.74088864940549	15.25470198099755
C	8.84878062170548	1.28990852012187	14.89232569971226
C	9.22284183018377	1.94710089378173	13.68801042430978
C	8.16202372709293	2.80691911588352	13.30984471487103
C	10.92968157393594	5.92303102604085	14.22522167398676
C	9.03971163745211	6.76223626743263	13.54279325035067
C	11.85082252312357	5.02811308160888	14.78960750701903
C	12.65582260025730	3.26292941657038	15.77847072534591
N	11.28812747219000	7.12037201655191	13.64242025037659
C	10.09992604382408	7.65546183066782	13.20078043809504
C	7.71845000928339	7.07020206575850	13.22291667038239
N	13.21175462860177	5.24829555805618	14.80681518731343
C	12.90461443171338	2.05962080966226	16.43640428216204
C	13.73041775286445	4.13497917015134	15.42683087195203
Y	13.55650683812565	6.89171909072802	13.20049918062887
C	9.83465445054537	8.84074068992807	12.51698244507154
C	7.46606833854396	8.26836900700644	12.54352614593648
H	6.91134876580712	6.39444121724800	13.48575419168574
H	12.09034871905314	1.39654221739811	16.70941559905690
C	14.23389399650769	1.72663096036692	16.72451524057537
C	15.04940073999074	3.78702985707442	15.71261285974541
C	14.30049334842929	9.43582461168203	13.28817102427508
C	15.50739570026012	8.69881642459293	13.15275692957249
C	15.70280639674648	7.95731212441435	14.34825734022392
C	14.62413704520690	8.24856397968211	15.22793016191791
C	13.75977178389705	9.16109586933351	14.57466288102217
C	14.18539544007210	4.84702741053614	11.63274514440386
C	14.92978306086431	5.95410329780634	11.14372268445976
C	14.01123452169715	6.89121769836565	10.59327365040185
C	12.70194768946248	6.36232099847259	10.74534590401744
C	12.81024846081352	5.10046744127478	11.38766829602999
H	10.63835678081991	9.51719725237061	12.24604706289259
C	8.50363718097482	9.13470670922013	12.19579268050581
H	6.44521933041360	8.52745060764837	12.28291062752508
H	14.44991459474848	0.79291147239283	17.23313913267614
C	15.28533597415084	2.57220707426896	16.36712731557094
H	15.86778788066286	4.44398044686783	15.43800821808410
H	8.27554303144426	10.05276682389760	11.66437303077937
H	16.30473931158039	2.28257346701984	16.59978075948305
H	13.87213417666078	10.10713163589674	12.54262181831110
H	16.16771674617066	8.70418328959131	12.28580209780695

H	16.54042109690358	7.29176931233746	14.56118383163371
H	16.01476110404338	6.06083045611147	11.17190635397676
H	12.82708162430577	9.55709971514888	14.97491002001222
H	14.47300214677445	7.81862409912106	16.21734264260649
H	11.77209952098233	6.84326718837695	10.44029595727931
H	14.26900243160791	7.84394855642825	10.12943151205457
H	14.59253243087181	3.96280023167177	12.12392205445647
H	11.98022165045396	4.45349991127061	11.67026110445561
H	10.17070392910645	1.82858110439601	13.16422458534104
H	9.45047789599782	0.55882801464292	15.43401671936565
H	6.98002477288541	1.41839062944514	16.12448477472938
H	6.17632100059490	3.21465348546182	14.27261217746238
H	8.15160151388306	3.46694359590246	12.44296507866061
H	10.61603732919159	3.97853254529654	18.49592973556335
H	10.11556408897525	6.38489205884015	17.38730458729887
H	7.51728354528227	6.46200018175279	16.67664273616995
H	6.39878673776251	4.08577439692851	17.33518069660601
H	8.31690100253371	2.54883028095520	18.46288600103481

1.10 References

(1) Evans, W. J.; Kozimor, S. A.; Ziller, J. W.; Kaltsoyannis, N. Structure, Reactivity, and Density Functional Theory Analysis of the Six-Electron Reductant, $[(C_5Me_5)_2U]_2(\mu\text{-}\eta^6\text{:}\ \eta^6\text{-}C_6H_6)$, Synthesized via a New Mode of $(C_5Me_5)_3M$ Reactivity. *J. Am. Chem. Soc.* **2004**, *126* (44), 14533–14547. https://doi.org/10.1021/ja0463886.

(2) Barker, B. J.; Sears, P. G. Conductance Behaviour of Some Ammonium and Partially Substituted Ammonium Tetraphenylborates in Oxazolidones. *J. Phys. Chem.* **1974**, *78* (26), 2687–2688.

(3) Demir, S.; Zadrozny, J. M.; Nippe, M.; Long, J. R. Exchange Coupling and Magnetic Blocking in Bipyrimidyl Radical-Bridged Dilanthanide Complexes. *J. Am. Chem. Soc.* **2012**, *134* (45), 18546–18549. https://doi.org/10.1021/ja308945d.

(4) Isoda, K.; Nakamura, M.; Tatenuma, T.; Ogata, H.; Sugaya, T.; Tadokoro, M. Synthesis and Characterization of Electron-Accepting Nonsubstituted Tetraazaacene Derivatives. *Chem. Lett.* **2012**, *41* (9), 937–939. https://doi.org/10.1246/cl.2012.937.

(5) Dütsch, L.; Fleischmann, M.; Welsch, S.; Balázs, G.; Kremer, W.; Scheer, M. Dicationic E4 Chains (E=P, As, Sb, Bi) Embedded in the Coordination Sphere of Transition Metals. *Angew. Chem. Int. Ed.* **2018**, *57* (12), 3256–3261. https://doi.org/10.1002/anie.201712884.

(6) Bergbreiter, D. E.; Killough, J. M. Reactions of Potassium-Graphite. *J. Am. Chem. Soc.* **1978**, *100* (7), 2126–2134. https://doi.org/10.1021/ja00475a025.

(7) Rigaku Corporation. CrysAlisPro Software System. Oxford 2020.

(8) Rigaku Corporation. SCALE3 ABSPACK: Empirical Absorption Correction, CrysAlis Pro - Software Package. Oxford 2020.

(9) Systems, B. A. X. COSMO V1.61, Software for the CCD Detector Systems for Determining Data Collection Parameters. Madison, Wisconsin 2009.

(10) Systems, B. A. X. APEX2 V2010.11-3. Software for the CCD Detector System. Madison, Wisconsin 2010.

(11) Systems, B. A. X. SAINT V 7.68A Software for the Integration of CCD Detector System. Madison, Wisconsin 2010.

(12) Blessing, R. H. SADABS V2.008/2 Program for Absorption Corrections Using Bruker-AXS CCD Based on the Method of Robert Blessing. *Acta Cryst.* **1995**, *A51*, 33–38. https://doi.org/10.1107/S0108767394005726.

(13) Sheldrick, G. M. SHELXT - Integrated Space-Group and Crystal-Structure Determination. *Acta Cryst.* **2015**, *A71*, 3–8. https://doi.org/10.1107/S2053273314026370.

(14) Sheldrick, G. M. Crystal Structure Refinement with SHELXL. *Acta Cryst.* **2015**, *C71*, 3–8. https://doi.org/10.1107/S2053229614024218.

(15) Dolomanov, O. V.; Bourhis, L. J.; Gildea, R. J.; Howard, J. A. K.; Puschmann, H. OLEX2: A Complete Structure Solution, Refinement and Analysis Program. *J. Appl. Crystallogr.* **2009**, *42* (2), 339–341. https://doi.org/10.1107/S0021889808042726.

(16) Stoll, S.; Schweiger, A. EasySpin, A Comprehensive Software Package for Spectral Simulation and Analysis in EPR. *J. Magn. Reson.* **2006**, *178* (1), 42–55. https://doi.org/10.1016/j.jmr.2005.08.013.

(17) Bain, G. A.; Berry, J. F. Diamagnetic Corrections and Pascal's Constants. *J. Chem. Edu.* **2008**, *85* (4), 532–536. https://doi.org/10.1021/ed085p532.

(18) Neese, F. The ORCA Program System. *Wiley Interdiscip. Rev. Comput. Mol. Sci.* **2012**, *2* (1), 73–78. https://doi.org/10.1002/wcms.81.

(19) Neese, F. Software Update: The ORCA Program System—Version 5.0. *Wiley Interdiscip. Rev. Comput. Mol. Sci.* **2022**, *12* (5), 1–15. https://doi.org/10.1002/wcms.1606.

(20) Staroverov, V. N.; Scuseria, G. E.; Tao, J.; Perdew, J. P. Erratum: Comparative Assessment of a New Nonempirical Density Functional: Molecules and Hydrogen-Bonded Complexes(Journal of Chemical Physics (**2003**) *119* (12129)). *J. Chem. Phys.* **2004**, *121* (22), 11507. https://doi.org/10.1063/1.1795692.

(21) S. Grimme; S. Ehrlich; L. Goerigk. Effect of the damping function in dispersion corrected density functional theory. *J. Comput. Chem.* **2011**, *32* (7), 1456–1465. https://doi.org/10.1002/jcc.21759.

(22) Neese, F.; Wennmohs, F.; Hansen, A.; Becker, U. Efficient, Approximate and Parallel Hartree–Fock and Hybrid DFT Calculations. A 'Chain-of-Spheres' Algorithm for the Hartree–Fock Exchange (RIJCOSX). *Chem. Phys.* **2009**, *356* (1-3), 98-109. https://doi.org/10.1016/j.chemphys.2008.10.036

(23) Izsák, R.; Neese, F. An Overlap Fitted Chain of Spheres Exchange Method. *J. Chem. Phys.* **2011**, *135* (14), 144105. https://doi.org/10.1063/1.3646921.

(24) Weigend, F. Accurate Coulomb-fitting basis sets for H to Rn. *Phys. Chem. Chem. Phys.* **2006**, *8* (9), 1057-1065.

(25) Peterson, K. A.; Figgen, D.; Dolg, M.; Stoll, H. Energy-Consistent Relativistic Pseudopotentials and Correlation Consistent Basis Sets for the 4d Elements Y–Pd. *J. Chem. Phys.* **2007**, *126* (12), 124101–124112.

(26) Reed, A. E.; Weinhold, F. Natural Localized Molecular Orbitals. *J. Chem. Phys.* **1985**, *83* (4), 1736–1740. https://doi.org/10.1063/1.449360.

(27) Lu, T.; Chen, F. Multiwfn: A Multifunctional Wavefunction Analyzer. *J. Comput. Chem.* **2012**, *33* (5), 580–592. https://doi.org/10.1002/jcc.22885.

(28) Weigend, F.; Ahlrichs, R. Balanced Basis Sets of Split Valence, Triple Zeta Valence and Quadruple Zeta Valence Quality for H to Rn: Design and Assessment of Accuracy. *Phys. Chem. Chem. Phys.* **2005**, *7* (18), 3297–3305. https://doi.org/10.1039/b508541a.

(29) Perdew, J. P.; Burke, K.; Ernzerhof, M. Generalized Gradient Approximation Made Simple. *Phys. Rev. Lett.* **1997**, *78* (7), 1396–1396. https://doi.org/S0031-9007(97)02302-8.

(30) Adamo, C.; Barone, V. Toward Reliable Density Functional Methods without Adjustable Parameters: The PBE0 Model. *J. Chem. Phys.* **1999**, *110* (13), 6158–6170. https://doi.org/10.1063/1.478522.

(31) van Wüllen, C. Molecular Density Functional Calculations in the Regular Relativistic Approximation: Method, Application to Coinage Metal Diatomics, Hydrides, Fluorides and Chlorides, and Comparison with First-Order Relativistic Calculations. *J. Chem. Phys.* **1998**, *109* (2), 392–399. https://doi.org/10.1063/1.476576.

(32) Hess, B. A. Applicability of the No-Pair Equation with Free-Particle Projection Operators to Atomic and Molecular Structure Calculations. *Phys. Rev. A* **1985**, *32* (2), 756–763. https://doi.org/10.1103/PhysRevA.32.756.

(33) Rolfes, J. D.; Neese, F.; Pantazis, D. A. All-Electron Scalar Relativistic Basis Sets for the Elements Rb–Xe. *J. Comput. Chem.* **2020**, *41* (20), 1842–1849. https://doi.org/10.1002/jcc.26355.

(34) Benner, F.; Demir, S. Isolation of the Elusive Bisbenzimidazole Bbim$^{3-\bullet}$ Radical Anion and Its Employment in a Metal Complex. *Chem. Sci.* **2022**, *13* (20), 5818–5829. https://doi.org/10.1039/D1SC07245E.

(35) Hanwell, M. D.; Curtis, D. E.; Lonie, D. C.; Vandermeersch, T.; Zurek, E.; Hutchison, G. R. Avogadro: An Advanced Semantic Chemical Editor, Visualization, and Analysis Platform. *J. Cheminform.* **2012**, *4* (17), 1-17. https://doi.org/10.1186/1758-2946-4-17.

(36) Xiang, Q.; Guo, J.; Xu, J.; Ding, S.; Li, Z.; Li, G.; Phan, H.; Gu, Y.; Dang, Y.; Xu, Z.; Gong, Z.; Hu, W.; Zeng, Z.; Wu, J.; Sun, Z. Stable Olympicenyl Radicals and Their π-Dimers. *J. Am. Chem. Soc.* **2020**, *142* (25), 11022–11031. https://doi.org/10.1021/jacs.0c02287.

(37) Small, D.; Zaitsev, V.; Jung, Y.; Rosokha, S. V.; Head-Gordon, M.; Kochi, J. K. Intermolecular π-to-π Bonding between Stacked Aromatic Dyads. Experimental and Theoretical Binding Energies and near-IR Optical Transitions for Phenalenyl Radical/Radical versus Radical/Cation Dimerizations. *J. Am. Chem. Soc.* **2004**, *126* (42), 13850–13858. https://doi.org/10.1021/ja046770i.

(38) Grampp, G.; Landgraf, S.; Rasmussen, K.; Strauss, S. Dimerization of Organic Free Radicals in Solution.: 1. Temperature Dependent Measurements. *Spectrochim. Acta Part A Mol. Biomol. Spectrosc.* **2002**, *58* (6), 1219–1226. https://doi.org/10.1016/S1386-1425(01)00712-0.

(39) Barluzzi, L.; Ogilvie, S. P.; Dalton, A. B.; Kaden, P.; Gericke, R.; Mansikkam, A.; Giblin, S. R.; Layfield, R. A. Triply Bonded Pancake π-Dimers Stabilized by Tetravalent Actinides. *J. Am. Chem. Soc.* **2024**, *146* (6), 4234-4241. https://doi.org/10.1021/jacs.3c13914.

(40) Eaton, G. R.; Eaton, S. S.; Barr, D. P.; Weber, R. T. Quantitative EPR, 1st ed.; Springer Vienna, 2010. https://doi.org/10.1007/978-3-211-92948-3.

(41) Wautelet, P.; Le Moigne, J.; Videva, V.; Turek, P. Spin Exchange Interaction through Phenylene-Ethynylene Bridge in Diradicals Based on Iminonitroxide and Nitronylnitroxide Radical Derivatives. 1. Experimental Investigation of the through-Bond Spin Exchange Coupling. *J. Org. Chem.* **2003**, *68* (21), 8025–8036. https://doi.org/10.1021/jo034723n.

(42) Aldenderfer, M.; Craig, N.; Speakman, R.; Popelka-Filcoff, R. High-Conductive Crystal of Copper (I) Coordination Polymers Bridged by TANC as New Organic. *Angew. Chem. Int. Ed.* **2006**, *45* (31), 5144-5147. https://doi.org/10.1002/anie.200600553.

(43) Mugiraneza, S.; Hallas, A. M. Tutorial: A Beginner's Guide to Interpreting Magnetic Susceptibility Data with the Curie-Weiss Law. *Commun. Phys.* **2022**, *5* (1). https://doi.org/10.1038/s42005-022-00853-y.
